# Myeloid cell-derived interleukin-6 induces vascular dysfunction and vascular and systemic inflammation

**DOI:** 10.1093/ehjopen/oeae046

**Published:** 2024-06-12

**Authors:** Tanja Knopp, Rebecca Jung, Johannes Wild, Magdalena L Bochenek, Panagiotis Efentakis, Annika Lehmann, Tabea Bieler, Venkata Garlapati, Cindy Richter, Michael Molitor, Katharina Perius, Stefanie Finger, Jérémy Lagrange, Iman Ghasemi, Konstantinos Zifkos, Katharina S Kommoss, Joumana Masri, Sonja Reißig, Voahanginirina Randriamboavonjy, Thomas Wunderlich, Nadine Hövelmeyer, Alexander N R Weber, Ilgiz A Mufazalov, Markus Bosmann, Ingo Bechmann, Ingrid Fleming, Matthias Oelze, Andreas Daiber, Thomas Münzel, Katrin Schäfer, Philip Wenzel, Ari Waisman, Susanne Karbach

**Affiliations:** Department of Cardiology—Cardiology I, University Medical Center of the Johannes Gutenberg-University Mainz, Langenbeckstr. 1, 55131 Mainz, Germany; Center for Thrombosis and Hemostasis (CTH), University Medical Center Mainz, Langenbeckstr. 1, 55131 Mainz, Germany; Department of Cardiology—Cardiology I, University Medical Center of the Johannes Gutenberg-University Mainz, Langenbeckstr. 1, 55131 Mainz, Germany; Center for Thrombosis and Hemostasis (CTH), University Medical Center Mainz, Langenbeckstr. 1, 55131 Mainz, Germany; Institute of Molecular Medicine, University Medical Center Mainz, Mainz, Germany; Department of Cardiology—Cardiology I, University Medical Center of the Johannes Gutenberg-University Mainz, Langenbeckstr. 1, 55131 Mainz, Germany; Center for Thrombosis and Hemostasis (CTH), University Medical Center Mainz, Langenbeckstr. 1, 55131 Mainz, Germany; German Center for Cardiovascular Research (DZHK), Partner site Rhine-Main, Germany; Department of Cardiology—Cardiology I, University Medical Center of the Johannes Gutenberg-University Mainz, Langenbeckstr. 1, 55131 Mainz, Germany; Center for Thrombosis and Hemostasis (CTH), University Medical Center Mainz, Langenbeckstr. 1, 55131 Mainz, Germany; German Center for Cardiovascular Research (DZHK), Partner site Rhine-Main, Germany; Laboratory of Pharmacology, Faculty of Pharmacy, National and Kapodistrian University of Athens, Athens, Greece; Department of Cardiology—Cardiology I, University Medical Center of the Johannes Gutenberg-University Mainz, Langenbeckstr. 1, 55131 Mainz, Germany; Center for Thrombosis and Hemostasis (CTH), University Medical Center Mainz, Langenbeckstr. 1, 55131 Mainz, Germany; Department of Cardiology—Cardiology I, University Medical Center of the Johannes Gutenberg-University Mainz, Langenbeckstr. 1, 55131 Mainz, Germany; Center for Thrombosis and Hemostasis (CTH), University Medical Center Mainz, Langenbeckstr. 1, 55131 Mainz, Germany; Center for Thrombosis and Hemostasis (CTH), University Medical Center Mainz, Langenbeckstr. 1, 55131 Mainz, Germany; Institute of Anatomy, University Medical Center Leipzig, Leipzig, Germany; Institute of Neuroradiology, University Medical Center, Leipzig, Germany; Department of Cardiology—Cardiology I, University Medical Center of the Johannes Gutenberg-University Mainz, Langenbeckstr. 1, 55131 Mainz, Germany; Center for Thrombosis and Hemostasis (CTH), University Medical Center Mainz, Langenbeckstr. 1, 55131 Mainz, Germany; German Center for Cardiovascular Research (DZHK), Partner site Rhine-Main, Germany; Center for Thrombosis and Hemostasis (CTH), University Medical Center Mainz, Langenbeckstr. 1, 55131 Mainz, Germany; Center for Thrombosis and Hemostasis (CTH), University Medical Center Mainz, Langenbeckstr. 1, 55131 Mainz, Germany; Center for Thrombosis and Hemostasis (CTH), University Medical Center Mainz, Langenbeckstr. 1, 55131 Mainz, Germany; Center for Thrombosis and Hemostasis (CTH), University Medical Center Mainz, Langenbeckstr. 1, 55131 Mainz, Germany; Center for Thrombosis and Hemostasis (CTH), University Medical Center Mainz, Langenbeckstr. 1, 55131 Mainz, Germany; Department of Dermatology, University Hospital Heidelberg, Heidelberg, Germany; Institute of Molecular Medicine, University Medical Center Mainz, Mainz, Germany; Institute of Molecular Medicine, University Medical Center Mainz, Mainz, Germany; Institute for Vascular Signalling, Centre for Molecular Medicine, Goethe University, Frankfurt am Main, Germany; Max Planck Institute for Metabolism Research Cologne, Cologne, Germany; Institute of Molecular Medicine, University Medical Center Mainz, Mainz, Germany; Research Center for Immunotherapy, University Medical Center of the Johannes Gutenberg-University Mainz, Mainz, Germany; Department of Innate Immunity, Eberhard Karls University Tübingen, Tübingen, Germany; Institute of Molecular Medicine, University Medical Center Mainz, Mainz, Germany; Center for Thrombosis and Hemostasis (CTH), University Medical Center Mainz, Langenbeckstr. 1, 55131 Mainz, Germany; Department of Medicine, Pulmonary Center, Boston University School of Medicine, Boston, MA 02118, USA; Institute of Anatomy, University Medical Center Leipzig, Leipzig, Germany; German Center for Cardiovascular Research (DZHK), Partner site Rhine-Main, Germany; Institute for Vascular Signalling, Centre for Molecular Medicine, Goethe University, Frankfurt am Main, Germany; Department of Cardiology—Cardiology I, University Medical Center of the Johannes Gutenberg-University Mainz, Langenbeckstr. 1, 55131 Mainz, Germany; Department of Cardiology—Cardiology I, University Medical Center of the Johannes Gutenberg-University Mainz, Langenbeckstr. 1, 55131 Mainz, Germany; Department of Cardiology—Cardiology I, University Medical Center of the Johannes Gutenberg-University Mainz, Langenbeckstr. 1, 55131 Mainz, Germany; Center for Thrombosis and Hemostasis (CTH), University Medical Center Mainz, Langenbeckstr. 1, 55131 Mainz, Germany; German Center for Cardiovascular Research (DZHK), Partner site Rhine-Main, Germany; Department of Cardiology—Cardiology I, University Medical Center of the Johannes Gutenberg-University Mainz, Langenbeckstr. 1, 55131 Mainz, Germany; German Center for Cardiovascular Research (DZHK), Partner site Rhine-Main, Germany; Department of Cardiology—Cardiology I, University Medical Center of the Johannes Gutenberg-University Mainz, Langenbeckstr. 1, 55131 Mainz, Germany; Center for Thrombosis and Hemostasis (CTH), University Medical Center Mainz, Langenbeckstr. 1, 55131 Mainz, Germany; German Center for Cardiovascular Research (DZHK), Partner site Rhine-Main, Germany; Institute of Molecular Medicine, University Medical Center Mainz, Mainz, Germany; Research Center for Immunotherapy, University Medical Center of the Johannes Gutenberg-University Mainz, Mainz, Germany; Department of Cardiology—Cardiology I, University Medical Center of the Johannes Gutenberg-University Mainz, Langenbeckstr. 1, 55131 Mainz, Germany; Center for Thrombosis and Hemostasis (CTH), University Medical Center Mainz, Langenbeckstr. 1, 55131 Mainz, Germany; German Center for Cardiovascular Research (DZHK), Partner site Rhine-Main, Germany

**Keywords:** Interleukin-6, Chronic inflammation, Myeloid cells, Endothelin-1, Vascular dysfunction

## Abstract

**Aims:**

The cytokine interleukin-6 (IL-6) plays a central role in the inflammation cascade as well as cardiovascular disease progression. Since myeloid cells are a primary source of IL-6 formation, we aimed to generate a mouse model to study the role of myeloid cell-derived IL-6 in vascular disease.

**Methods and results:**

Interleukin-6-overexpressing (IL-6^OE^) mice were generated and crossed with LysM-Cre mice, to generate mice (LysM-IL-6^OE^ mice) overexpressing the cytokine in myeloid cells. Eight- to 12-week-old LysM-IL-6^OE^ mice spontaneously developed inflammatory colitis and significantly impaired endothelium-dependent aortic relaxation, increased aortic reactive oxygen species (ROS) formation, and vascular dysfunction in resistance vessels. The latter phenotype was associated with decreased survival. Vascular dysfunction was accompanied by a significant accumulation of neutrophils, monocytes, and macrophages in the aorta, increased myeloid cell reactivity (elevated ROS production), and vascular fibrosis associated with phenotypic changes in vascular smooth muscle cells. In addition to elevated *Mcp1 and Cxcl1* mRNA levels, aortae from LysM-IL-6^OE^ mice expressed higher levels of inducible NO synthase and endothelin-1, thus partially accounting for vascular dysfunction, whereas systemic blood pressure alterations were not observed. Bone marrow (BM) transplantation experiments revealed that vascular dysfunction and ROS formation were driven by BM cell-derived IL-6 in a dose-dependent manner.

**Conclusion:**

Mice with conditional overexpression of IL-6 in myeloid cells show systemic and vascular inflammation as well as endothelial dysfunction. A decrease in circulating IL-6 levels by replacing IL-6-producing myeloid cells in the BM improved vascular dysfunction in this model, underpinning the relevant role of IL-6 in vascular disease.

## Graphical Abstract

**Graphical Abstract:**
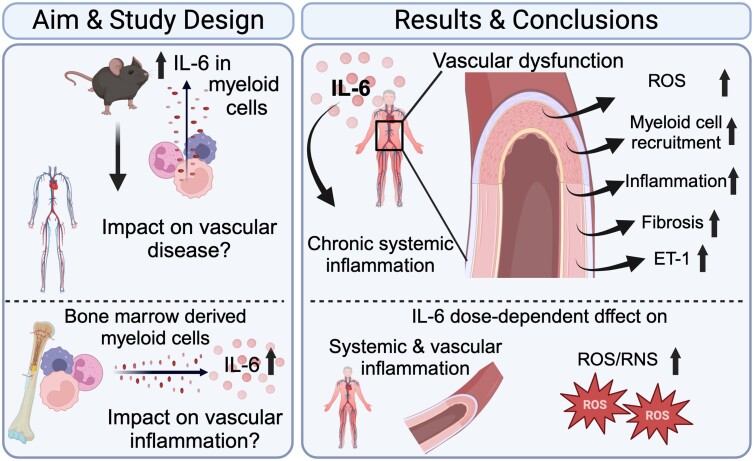
Myeloid cell-derived interleukin-6 (IL-6) overexpression was associated with systemic inflammation. Chronic myeloid cell-derived IL-6 evoked a significantly impaired endothelium-dependent aortic relaxation, increased aortic reactive oxygen species (ROS), and vascular dysfunction in resistance vessels. Vascular dysfunction was accompanied by a significant accumulation of myeloid cells in the aortic wall, an increased reactivity of myeloid cells, vascular fibrosis, and an altered vascular smooth muscle cell phenotype. This went in line with significantly elevated *Mcp1*, *Cxcl1*, and *Rorc* levels and an increased inducible NO synthase and endothelin-1 expression in LysM-IL-6^OE^ aortas. Bone marrow (BM) transplantation studies revealed that vascular dysfunction and ROS formation were driven by BM cell-derived IL-6 in a dose-dependent manner.

## Introduction

Cardiovascular disease (CVD) remains the leading cause of death worldwide. Vascular inflammation plays a crucial role in the development of CVD. Among the different mediators of the immune system, the cytokine interleukin-6 (IL-6) is of central relevance and has been repeatedly implicated in the pathogenesis of CVD. Interleukin-6 is a predictor of long-term cardiovascular mortality in patients with acute coronary syndrome,^1,2^ involved in heart failure,^3^ and has been suggested to predict the onset of coronary artery disease (CAD).^4^ It was described to be independently associated with the risk of major adverse cardiovascular events, cardiovascular and all-cause mortality, myocardial infarction, and heart failure in patients with stable coronary heart disease.^5^ Within the inflammation cascade, IL-6 has pivotal functions at the crossroads of the innate and adaptive immune responses. It is mainly produced by myeloid cells, stimulates the differentiation of pathogenic Th17 cells, and leads to activation and recruitment of neutrophil granulocytes, causing an amplification of the inflammatory response.^6–11^ Moreover, IL-6 plays a driving role in various autoimmune diseases such as psoriasis,^12^ rheumatoid arthritis,^13^ systemic lupus erythematosus,^14^ and autoimmune colitis,^15^ all of which have been shown to be associated with an increased cardiovascular risk.^16–19^ Thus, IL-6 represents a potential mediator driving increased cardiovascular comorbidities in patients with these chronic inflammatory disease states.^20^ Interleukin-6 deficiency was described to attenuate angiotensin II (AngII)-induced vascular dysfunction in mice.^21^ Nevertheless, the mechanisms and interactions of IL-6 in CVD are incompletely understood.^22^ As myeloid cells are one of the relevant physiological sources of IL-6,^23^ we generated mice with chronic myeloid IL-6 overexpression and analysed their vascular phenotype in order to determine the role of myeloid cell-derived IL-6 for systemic and vascular inflammation.

## Methods

### Mice

**Figure 1:**
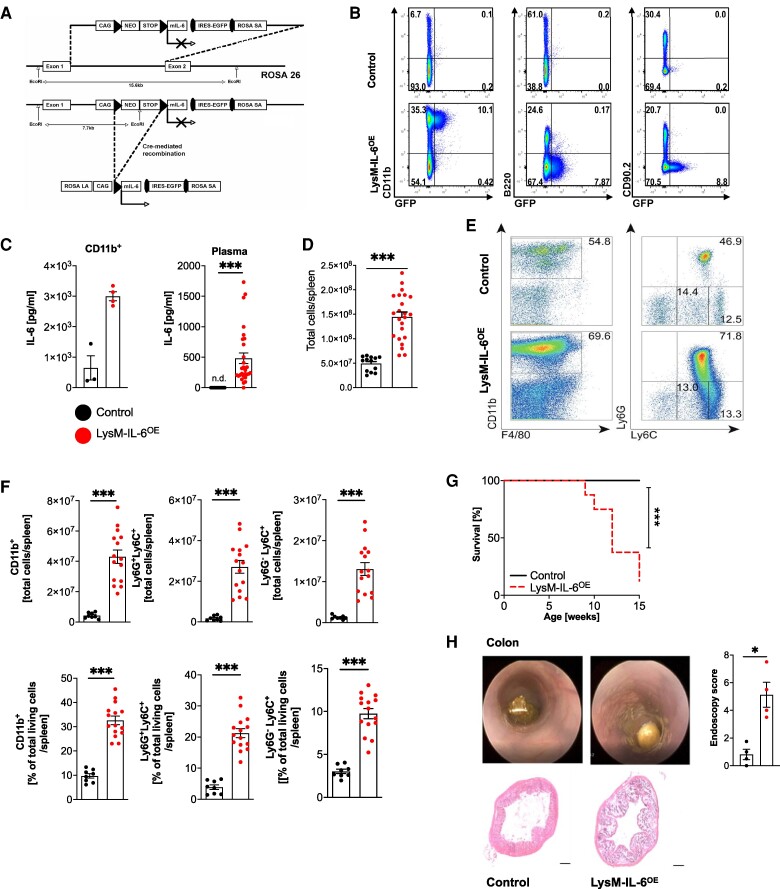
**Mice overexpressing interleukin-6 in myeloid cells have an increase in the splenic myeloid cell compartment and a reduced life expectancy.** (*A*) Generation of the IL-6^OE^ allele involved homologous recombination in embryonic stem cells (V6.5). The conditional ‘knock-in’ approach targeted the endogenous gt(ROSA)26Sor locus, introducing a lox-P-flanked transcriptional STOP cassette. Upon Cre-mediated recombination, the cassette was excised, enabling dual expression of interleukin-6 and enhanced green fluorescent protein under the control of the chicken β-actin (CAG) promotor. (*B*) Flow cytometric analysis of splenocytes from LysM-IL-6^OE^ and control mice. Cells were stained for CD11b, B220, and CD90.2 with gating based on green fluorescent protein signal. Representative plots of *n* = 4 mice are shown. (*C*) Interleukin-6 levels in splenocytes and plasma. Left panel: interleukin-6 level in sorted and cultured CD11b^+^ splenocytes of LysM-IL-6^OE^ mice and control mice. Splenocytes were cultured for 24 h. *n* = 3–4, Mann–Whitney test. Right panel: interleukin-6 plasma levels in 10-week-old LysM-IL-6^OE^ compared with control mice (n.d. = not detectable). *n* = 19–28, Mann–Whitney test. *P* < 0.0001. (*D*) Statistical analysis of the total living cells per spleen of LysM-IL-6^OE^ and control mice is shown (result of flow cytometric analysis), *n* = 13–23, Mann–Whitney test. *P* < 0.0001. (*E*) Flow cytometric analysis of LysM-IL-6^OE^ and control mice splenocyte subpopulations: after gating out B220^+^ and CD90.2^+^ cells, analysis focused on CD11b, F4/80, Ly6C, and Ly6G. Representative plots of *n* = 8–15 mice are shown. (*F*) Statistical analysis of the splenic CD11b^+^ myeloid cells, the Ly6G ^+^ Ly6C^+^ neutrophils, and the Ly6G^−^Ly6C^+^ monocytes/macrophages of LysM-IL-6^OE^ and control mice of the flow cytometric experiment above, *n* = 8–15 mice. Top row: total cells. Bottom row: percentage values of the total living cells, unpaired Student’s *t*-test. *P* < 0.0001. (*G*) Kaplan–Meier survival curve of LysM-IL-6^OE^ vs. control mice, *n* = 8–16 mice, log-rank (Mantel–Cox) test. (*H*) Representative image of colon endoscopy (left). Statistical analysis of endoscopy score (right), *n* = 4, Mann–Whitney test. Bottom part: representative haematoxylin and eosin stainings of the colon of LysM-IL-6^OE^ and control mice. Data are presented as mean ± SEM, and *P* values of <0.05 were considered significant and marked by asterisks (*P < 0.05; **P < 0.01; ***P < 0.001).

Interleukin-6-overexpressing mice were generated by gene targeting of embryonic stem (ES) cells. In brief, we have constructed a targeting vector by inserting the cDNA coding for IL-6 followed by an internal ribosome entry site and the cDNA coding for enhanced green fluorescent protein (eGFP) (as shown in *Figure 1A*). The conditional ‘knock-in’ approach targeted the endogenous gt(ROSA)26Sor locus, introducing a lox-P-flanked transcriptional STOP cassette (*Figure 1A*). Following homologous recombination into C57BL/6-V6.5 ES cells, we generated the IL-6^OE^ mice by injection of ES cells into blastocysts. This new mouse strain established on the C57BL/*6J* background allows cell-specific overexpression of IL-6 combined with eGFP expression (IL-6^OE^ mouse strain) upon Cre-mediated control. By crossing homozygous IL-6^OE/OE^ female mice with LysMCre^+^ male mice,^24^ we obtained **LysM-IL-6^OE^ mice** with heterozygous expression of LysMCre and heterozygous overexpression of IL-6 in LysM^+^ cells. Control mice were LysMCre negative and heterozygous for the IL-6^OE^ allele (**IL-6^OE^ mice**). At the age of 8–12 weeks, mice were used for experiments. Both female and male mice were examined, and the results were pooled as no sex-specific effect was observed.

Mice were euthanized by exsanguination under deep isoflurane anaesthesia (5% inhalant in room air) combined with tamasic (buprenorphine) as a single subcutaneous injection (0.075 mg/kg body weight) 30 min before the procedure to avoid any pain. Then, blood was collected by right ventricular puncture. Afterwards, organs, namely aorta, spleen, bone marrow (BM), heart, brain, colon, lungs, and kidneys, were harvested.

Housing, treatment, and euthanasia of animals were performed in accordance with the relevant laws and institutional guidelines of the Central Animal Facility of the UMC Mainz, Germany, and state authorities (G15-1-051, G15-1-101, G21-1-025, and G21-1-030). Experimental procedures including anaesthetic and analgesic treatments followed the guidelines from Directive 2010/63/EU of the European Parliament on the protection of animals used for scientific purposes. Experiments were conducted according to the ARRIVE guidelines.

### Vascular relaxation studies

Murine aortas were isolated and cut into 4 mm long segments, and the thoracic part of the aortas was utilized for aortic constriction and dilatation assays. They were mounted in organ chambers to measure endothelium or smooth muscle-dependent relaxation or contraction in response to acetylcholine (ACh), glyceryl trinitrate, potassium chloride (KCl), and prostaglandin F2α (PGF2α) as described previously.^25^

The ROS scavengers catalase (2000U/mL), superoxide dismutase (SOD) (2000U/mL), or the endothelin-1 (ET-1) antagonist bosentan (10 µM) were suspended in full medium (MV2 kit, PromoCell), and the isolated aorta segments were incubated in it for 90 min at 37°C and 5% CO_2_. Afterwards, relaxation studies were performed.

Besides the aortic analysis, the first and second branches of mesenteric arteries were isolated, cut into 2 mm long segments, and mounted in a myograph to measure relaxation in response to ACh or sodium nitroprusside or contraction in response to phenylephrine or KCl as described previously.^26,27^

### Echocardiography

Mouse echocardiography was performed using a VEVO-3100 ultrasound machine (FUJIFILM VisualSonics Inc., Toronto, Canada) equipped with a 38 MHz (MX400) linear array transducer. Standardized images in parasternal long axis (PLAX) and parasternal short axis as well as apical four-chamber view were recorded under isoflurane sedation with 1.5–2 vol%. In parallel, electrocardiogram and respiration rate were monitored and body temperature was kept stable using a heating system within the handling platform. Post-acquisition analysis was performed with the Vevo LAB software (VisualSonics, FujiFilm, Toronto, Canada) for left ventricular (LV) ejection fraction (PLAX B-mode), LV mass, stroke volume, and cardiac output (PLAX M-mode) and right heart function with right ventricular end-diastolic (mid-ventricular diameter) (4C and SAX) and pulmonary artery velocity time integral (SAX).

### Blood pressure measurement

Systolic blood pressure was measured using a tail–cuff non-invasive blood pressure system as previously described.^28,29^

### Analysis of reactive oxygen and nitrogen species in the blood and aortic tissue

Reactive oxygen species (ROS)/reactive nitrogen species (RNS) were measured in whole blood by L012-enhanced chemiluminescence after incubation with phorbol 12,13-dibutyrate (PDBu) (10 μmol/L) for 20 min:^30–32^ 200 IU of heparine was injected into the beating heart of the mouse and venous blood drawn from the right ventricle subsequently. Chemiluminescence was measured directly in the heparinized blood which was kept at room temperature (RT). L012-enhanced chemiluminescence signals were counted in 10 μL of samples with 100 μmol/L of L012 in the presence of PDBu using a Lumat LB 9507 from Berthold Technologies (Bad Wildbad, Germany). Chemiluminescence was expressed as counts per minute after incubation for 10 min.

Visualization of ROS in aortic cryosections was performed by staining with the superoxide-sensitive dye dihydroethidium (DHE, 1 µM) as previously described.^32^ Mouse aortas were rinsed, cleaned, and then cut into 4 mm sections which were then incubated in Krebs–Henseleit solution (containing 0.1 mg/mL of aprotinin, 0.2 mg/mL of pepstatin, and 0.5 mg/mL of leupeptin) for 10 min at 37°C. Then, the aortic sections were embedded in Tissue-Tek and frozen in liquid nitrogen. 8 µm aortic cryosections were cut and stained with DHE (incubation over 30 min at 37°C). Green autofluorescence from aortic lamina and red ethidium fluorescence inside the ROS-producing cells were detected by fluorescence light microscopy using a Zeiss Axiovert 40 CFL microscope, Zeiss lenses and Axiocam MRm camera (Zeiss, Oberkochen, Germany) and analysed with the AxioVision data acquisition software (Zeiss).

### *In vivo* high-resolution endoscopic analysis of the colon

A high-resolution video endoscopic system (Karl Storz SE & Co AG, Tuttlingen, Germany) was used for the analysis of the colitis-like phenotype. Colitis scores were determined on mice anaesthetized with a mixture of ketamine (Ketavest 100 mg/mL; Pfizer, New York, NY) and xylazine (Rompun 2%; Bayer Healthcare, Leverkusen, Germany) intraperitoneally. Endoscopic scoring of five parameters, namely translucency, granularity, fibrin, vascularity, and stool, was performed as previously described.^33^

### Bone marrow transfer

Recipient C57BL/6 Ly5.1 mice were irradiated with 9.5 Gy from Cs137 (OB58-BA; Buchler, Braunschweig, Germany). Mice were treated orally with Borgal antibiotic 2 weeks prior and 1 week after irradiation. Bone marrow cells were isolated from femurs and tibias of LysM-IL-6^OE^ or control mice, and 5*10^6^ BM cells were transplanted into recipient mice by injection into the tail vein. Different relative amounts of LysM-IL-6^OE^ BM (100% of control BM, 100% of LysM-IL-6^OE^ BM, 50% LysM-IL-6^OE^ BM, or 10% LysM-IL-6^OE^ BM) were transferred. Mice were analysed 10 weeks after BM transplantation.

### Flow cytometric analysis

First, aortic and splenic single-cell solutions were incubated with anti-CD16/anti-CD32 antibodies to prevent unspecific antibody bindings. Then, cells were stained with fluorophore-coupled monoclonal antibodies for CD11b, F4/80, Ly6G, Ly6C, B220, or CD90.2, together with a viability dye. Cells were acquired using either the FACSCanto™ II cytometer (BD, USA) or Attune™ NxT (Thermo Fisher).

### Flow cytometric reactive oxygen species analysis

For flow cytometric ROS analysis, whole blood was drawn by cardiac puncture and anticoagulated with ethylenediaminetetraacetic acid. Cells were treated with and without PDBu for 15 min at RT followed by incubation with CellROX® reagent (a fluorogenic indicator for the detection of ROS in cells, which is non-fluorescent but shows a strong fluorescence when oxidized; 5 μM, Thermo Fisher Scientific) for 30 min at 37°C, as previously described.^34^ Cells incubated in the absence of CellROX® reagent were used as a negative control. Afterwards, cells were stained [CD45—brilliant violet 510 (Clone: 30-F11), CD90.2—phycoerythrin (PE) (Clone: 53-2.1), CD11b—phycoerythrin–cyanine 7 (PE-Cy7) (Clone: M1/70), Ly6G—Super Bright 600 (Clone: 1A8), Ly6C—peridinin chlorophyll protein–Cy5.5 (Clone: KH1.4), and viability dye eFlour 708] and incubated for 20 min at RT. After dilution (final concentration 1:2000), cells were analysed using the Invitrogen™ Attune™ NxT flow cytometer (Thermo Fisher Scientific, Waltham, MA, USA) and the Invitrogen™ Attune™ No-Wash No-Lyse Filter Kit (Thermo Fisher Scientific, Waltham, MA, USA).

### Cytokine and chemokine quantification

Plasma IL-6 levels were measured with BD OptEIA™ mouse ELISA kit. Absorption was measured using the infinite M200 PRO NanoQuant plate reader (Tecan, Austria) or with the Bioplex Multiplex Immunoassay System ( BioRad, Germany), according to the manufacturer’s protocol.

### Cell culture

Human pulmonary artery endothelial cells (HPAECs, ATCC) were cultivated, as previously published.^35^ Cells were stimulated with 50 or 500 pg/mL of IL-6 (PeproTech, UK) with soluble IL-6 receptor (sIL-6R, PeproTech, UK).

### Isolation and cultivation of vascular smooth muscle cells

Vascular smooth muscle cells were isolated from the aortas of LysM-IL-6^OE^ mice and cultured as previously described^36^ In brief, aortas were carefully dissected and perfused with 1 × Phosphate buffered Saline (Gibco) to remove any blood. The aorta was kept in Dulbecco’s modified Eagle medium (DMEM; GlutaMAX-I; Gibco) with 10% fetal bovine serum (FBS; Gibco), 100 U/mL penicillin, and 100 μg/mL streptomycin (Gibco). After removing the perivascular fat under the microscope, aortas were cut into small pieces and digested in medium containing collagenase II (10 mg/mL) for 3 h in a thermo-shaker. Then, the medium was removed by centrifugation (400 g for 10 min), and cells were resuspended in DMEM containing 10% FBS, 100 U/mL penicillin, and 100 μg/mL streptomycin on 0.1% gelatin-coated six-well plates. Cells were analysed at passage 0.

### Histology

At the time of sacrifice, aorta, spleen, BM, heart, brain, colon, lungs, and kidneys were isolated from the experimental mice, fixed in 4% paraformaldehyde, paraffin-embedded, cut, and stained with haematoxylin and eosin according to standard protocols.^37^ Aortic sections were stained for collagen fibres using Sirius Red and analysed by polarized light microscopy.

### Immunohistochemistry

Aortic segments were fixed in 4% paraformaldehyde, paraffin-embedded, and stained with the primary antibody (anti-ET-1, 1:500, Meridian Life Science, USA) followed by incubation with the biotinylated secondary antibody after dilution. For immunochemical detection, ABC reagent (Vector) and then DAB reagent (peroxidase substrate Kit, Vector) as substrates were applied.^38^

### Immunocytochemistry

Vascular smooth muscle cells were fixed in 4% paraformaldehyde for 15 min at RT and stained with antibodies against smooth muscle alpha-actin (SMA; dilution 1:100; Sigma, A2547) and Ki67 (dilution 1:100, Abcam, ab15580). Phalloidin–rhodamine (dilution 1:300; Life Technology, R415) was used to visualize the cytoskeleton and 4′,6-Diamidin-2-phenylindol (DAPI) to visualize cell nuclei.

### RNA isolation and quantitative real-time PCR

Aortas and cells were lysed and homogenized in TRIzol® (Thermo Fisher) and processed following the manufacturer’s instructions. RNA concentration was measured using a NanoDrop spectrophotometer (Thermo Fisher). The TaqMan® Gene Expression Assay (Applied Biosystems™) was used with 0.5 μg of total RNA for quantitative real-time PCR for *Tatabox-binding protein* (*Tbp*) (Mm01277042_m1), *Mcp1* (Mm00441242_m1), *Vcam1* (Mm00449197_m1), *Cxcl1* (Mm04207460_m1), *Cxcl2* (Mm00436450_m1), *Tnf Mm00443260_g1*), *Il6* (Mm99999064_m1), *Rorc* (Mm01261022_m1), *ET-1* (Mm00438656_m1), *Il1beta* (Mm00434228_m1), *Stat3* (Mm01219775_m1), *Vegf-a* (Mm01281449_m1), *Nox2* (Mm00432775_m1), *Col1α1* (Mm00801666_g1), *Col1α2* (Mm00483888_m1), *MMP2* (Mm00439498_m1), and *MMP9* (Mm00442991_m1). For human material the following Primers were used: *Tbp* (Hs00427620_m1), *Il6* (Hs00174131_m1), *Et-1* (Hs00174961_m1). Besides, the following primers were used: *glyceraldehyde-3-phosphate dehydrogenase* (*Gapdh*) (forward: TAC CCC CAA TGT GTC CTG CTG G; reverse: CCT TCA GTG GGC CCT CAG ATG C)*; Col3α1* (forward: GAG GGC CAT AGC TGA ACT GA; reverse: TGA CTG TCC CAC GTA AGC AC), *Vimentin* (forward: CGG AAA GTG GAA TCC TTG CA; reverse: CAC ATC GAT CTG GAC ATG CTG T), and *αSma* (forward: GGACGTACAACTGGTATTGTGC; reverse: CGGCAGTAGTCACGAAGGAAT).

Quantification of mRNA expression as relative expression levels of the respective samples to *Tbp* or *Gapdh* as endogenous control (housekeeping genes) was calculated with the delta–delta threshold cycle method.^39^

### Western blot analysis

Isolated aortic tissue was homogenized, and protein was isolated using Radio-Immunoprecipitation assay buffer (Sigma-Aldrich) containing a protease/phosphatase inhibitor cocktail (Thermo Scientific). Aortic lysates were electrophorized on a 7–12% gradient gel. Proteins were transferred on a polyvinylidene difluoride membrane (pore size 0.45 μm). Immunoblotting was performed with the following primary antibodies: inducible NO synthase (iNOS) (BD Bioscience), ET-1 (Abcam), and alpha-/beta-actinin (Cell Signaling Technologies). For detection with automated imaging equipment (FUSION CCD Imager), either anti-mouse or anti-rabbit (Cell Signaling Technologies) secondary antibodies coupled to horseradish peroxidase in combination with ECL Western Blotting Substrate (Thermo Scientific) were used. Final quantification was performed by densitometric analysis (GelPro-analyzer).

### Statistical analysis

Data are displayed as mean ± SEM. Statistical calculation was done with GraphPad Prism software (version 9; GraphPad Software Inc.). Kolmogorov–Smirnov test was used for analysis of normal distribution. In the case of normal distribution, we performed the unpaired Student’s *t*-test to compare two experimental groups and the one-way analysis of variance (ANOVA) test with Tukey’s post hoc test for comparison of more than two groups. If non-normal distribution was given, Mann–Whitney test was applied for comparison of two groups and Kruskal–Wallis with Dunn’s multiple comparison test to compare more than two groups as indicated in the figure legends. Comparison of aortic relaxation curves and survival curves was made using a two-way ANOVA with Bonferroni’s post hoc test and log-rank (Mantel–Cox) test, respectively. *P* values of <0.05 were considered significant and marked by asterisks (*P < 0.05; **P < 0.01; ***P < 0.001).

## Results

### Myeloid cell-derived interleukin-6 evokes a chronic systemic inflammatory state with an expansion of the myeloid cell compartment paralleled by vascular dysfunction in mice

To understand the function of IL-6 in vascular disease, we generated mice with conditional IL-6 overexpression (IL-6^OE/OE^ mouse strain). Crossing the IL-6^OE/OE^ mouse strain with LysM-Cre^+^ mice^24^ resulted in mice in which myeloid cells overexpress IL-6 and eGFP (LysM-IL-6^OE^ mice, *Figure 1A*). We confirmed this by flow cytometry of splenocytes (*Figure 1B*) and IL-6 ELISA of the supernatants of isolated and *in vitro* cultured CD11b^+^ cells (*Figure 1C*, left). Myeloid IL-6 overexpression resulted in significantly elevated circulating IL-6 plasma levels compared with non-detectable concentrations in littermate controls (*Figure 1C*, right). Of note, the IL-6 levels in these mice were considerably lower than those reported during acute sepsis.^40^

The increase in systemic IL-6 was accompanied by an increase in splenic total cell numbers shown by flow cytometric analysis in the LysM-IL-6^OE^ mice (*Figure 1D*). We found an expansion of splenic CD11b^+^ myeloid cells, CD11b ^+^ Ly6G ^+^ Ly6C^+^ neutrophil granulocytes, and CD11b ^+^ Ly6G^−^Ly6C^+^ monocytes/macrophages (shown as total cell counts and the percentage of viable splenic cells, *Figure 1E* and *F*; gating strategy is shown in Supplementary material online, *Figure S1A*).

The CD11b^+^ myeloid cells and the CD11b ^+^ Ly6G ^+^ Ly6C^+^ neutrophil granulocytes were also increased in the blood, whereas the number of systemic CD11b ^+^ Ly6G^−^Ly6C^+^ monocytes/macrophages was not altered (see Supplementary material online, *Figure S1B* and *C*). There was no change in blood CD90.2^+^ T cells detectable in the LysM-IL-6^OE^ mice compared with controls (see Supplementary material online, *Figure S1D*). The total number of splenic T cells was not altered either, but a reduction in the percentage of splenic T cells of total living cells was apparent (see Supplementary material online, *Figure S1E*).

Chronic elevations of circulating IL-6 levels were associated with an increased mortality in mice. Starting premature death at 9 weeks, only 12.5% of LysM-IL-6^OE^ mice reached 15 weeks of age (*Figure 1G*). To better understand the reduced lifespan in the LysM-IL-6^OE^ mice, we cautiously monitored the mice and performed detailed (histological) analysis of 11–12-week-old LysM-IL-6^OE^ mice compared with control mice (*Figure 1H* and Supplementary material online, *Figure S2*). It was apparent that the LysM-IL-6^OE^ mice were prone to the development of intestinal prolapses. This was accompanied by a colitis-like phenotype shown by histological analysis and endoscopy (*Figure 1H*). Additionally, LysM-IL-6^OE^ mice revealed inflammatory changes in the lungs, liver, spleen, and kidney (see Supplementary material online, *Figure S2A–D*), all of which—in particular the pulmonary inflammation—could contribute to premature death. The brain was apparently histologically not affected (see Supplementary material online, *Figure S2E*). There were no macro- or microscopic signs of thrombosis or bleeding in all analysed organs (see Supplementary material online, *Figure S2*). In summary, LysM-IL-6^OE^ mice developed signs of systemic inflammation with an IL-6-driven colitis-like phenotype.

**Figure 2:**
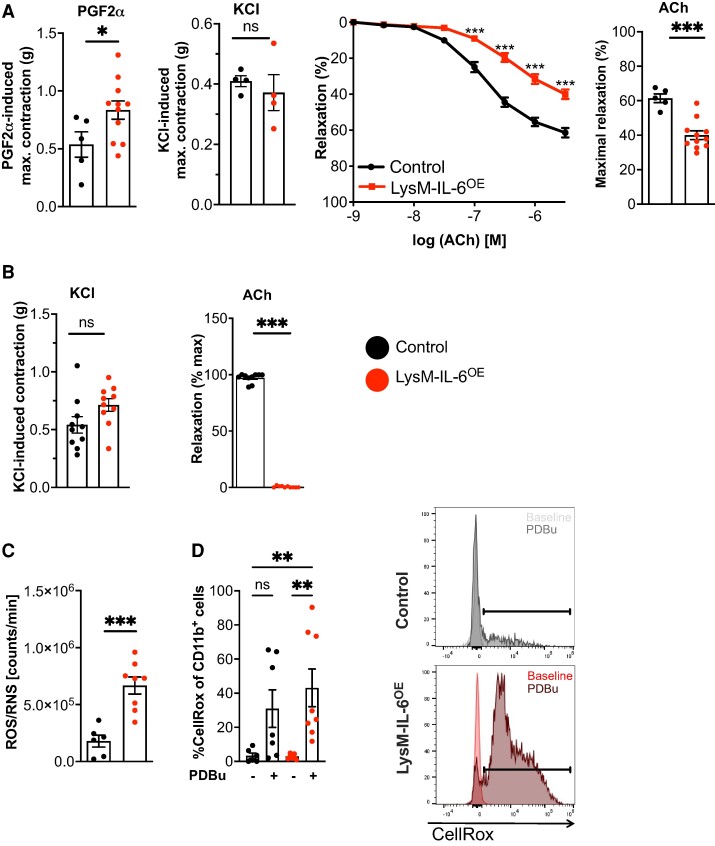
**Interleukin-6 overexpression in myeloid cells evokes significant vascular dysfunction, increased oxidative stress formation, and an increased reactivity of myeloid cells.** (*A*) Aortic constriction studies. Left panel: aortic constriction in response to prostaglandin F2α and potassium chloride, analysed by unpaired Student’s *t*-test. Middle panel: isometric tension studies of LysM-IL-6^OE^ and control aortas in response to acetylcholine, analysed by two-way ANOVA with Bonferroni’s multiple comparisons. Right panel: statistical analysis of acetylcholine -induced maximal relaxation with unpaired Student’s *t*-test. *n* = 5–11 mice. *P* = 0.0499 (prostaglandin F2α), *P* = 0.0002 (maximal relaxation acetylcholine). (*B*) Mesenteric artery contraction and relaxation. Left panel: contraction of mesenteric arteries in response to potassium chloride in LysM-IL-6^OE^ and control mice, analysed by unpaired Student’s *t*-test. Right panel: relaxation to acetylcholine of mesenteric arteries that have been precontracted with phenylephrine, Mann–Whitney test, *n* = 8. *P* < 0.0001 (maximal relaxation acetylcholine). (*C*) Reactive oxygen species/reactive nitrogen species levels in whole blood after 20 min stimulation with phorbol 12,13-dibutyrate in LysM-IL-6^OE^ vs. controls, *n* = 6–8, unpaired Student’s *t*-test. *P* = 0.003. (*D*) Flow cytometric analysis of reactive oxygen species levels detected by CellRox Deep Red staining in CD11b^+^ myeloid cells in blood of LysM-IL-6^OE^ mice compared with controls with and without phorbol 12,13-dibutyrate stimulation. Pre-gating on living and CD45.2^+^ cells. Kruskal–Wallis test with Dunn’s multiple comparison test. Quantification left panel: representative flow cytometry histograms; right panel. *n* = 7–8 mice. *P* = 0.0045 (control vs. LysM-IL-6^OE^ + phorbol 12,13-dibutyrate), *P* = 0.0035 (LysM-IL-6^OE^ vs. LysM-IL-6^OE^ + phorbol 12,13-dibutyrate). Data are presented as mean ± SEM, and *P* values of <0.05 were considered significant and marked by asterisks (*P < 0.05; **P < 0.01; ***P < 0.001).

We then focused on the analysis of the vascular system. Maximal constriction of aortic rings from LysM-IL-6^OE^ mice to PGF2α was greater than that of control mice, whereas responses to KCl did not differ between genotypes (*Figure 2A*). Vessels from LysM-IL-6^OE^ mice also demonstrated endothelial dysfunction evidenced by an impaired ACh-induced relaxation (*Figure 2A*). On the other hand, endothelium-independent vascular relaxation was comparable in segments from wild-type (WT) and LysM-IL-6^OE^ mice, as was blood pressure (see Supplementary material online, *Figure S3A* and *B*). In mesenteric arteries, contractile responses to KCl were also unaffected by the overexpression of IL-6 in myeloid cells (*Figure 2B*). Additionally, ACh-induced relaxation was abrogated in mesenteric arteries from LysM-IL-6^OE^ mice (*Figure 2B*). This indicated a clear link between a myeloid cell-derived cytokine and severe endothelial dysfunction that was independent of any change in VSMC reactivity or blood pressure (see Supplementary material online, *Figure S3C*). Endothelial dysfunction is linked to increased ROS production,^41^ and ROS/RNS levels were significantly increased in blood from LysM-IL-6^OE^ mice (*Figure 2C*). Furthermore, the CD11b^+^ myeloid cells demonstrated an increased ROS production following PDBu stimulation by trend, underpinning their increased reactivity (*Figure 2D*). We detected no differences in the heart size or function in the LysM-IL-6^OE^ mice compared with controls. There were no structural or functional differences in the heart, as assessed by histology and echocardiography (see Supplementary material online, *Figure S4*).

Taken together, these data demonstrate that the chronic systemic inflammatory state evoked by chronic myeloid IL-6 overexpression is associated with vascular dysfunction without significant cardiac alterations.

### Interleukin-6-induced vascular dysfunction is associated with vascular inflammation, increased reactive oxygen species formation and endothelin-1 expression, vascular smooth muscle cell phenotype alterations, and fibrosis in the vasculature

**Figure 3:**
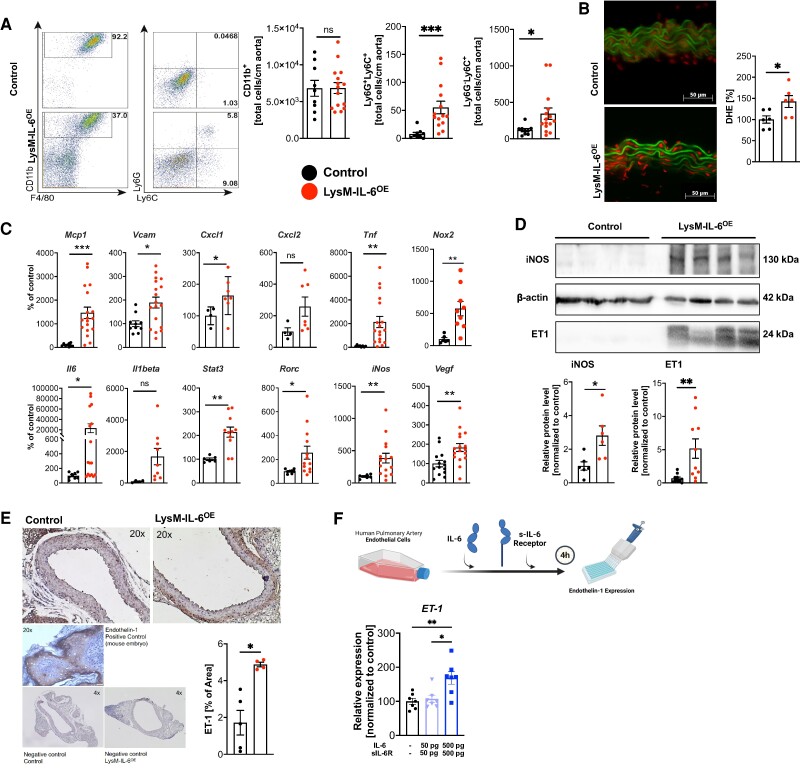
**Interleukin-6-induced vascular dysfunction is based on vascular inflammation combined with an increased endothelin-1 and inducible NO synthase expression.** (*A*) Myeloid cell infiltration in aortas. Representative flow cytometry plots and statistical analysis of myeloid surface staining of aortas from LysM-IL-6^OE^ mice compared with controls and quantification of aortic myeloid cell staining. Pre-gating on living, CD45.2^+^, and CD11b^+^ cells. Neutrophils and monocytes were further gated on Ly6G and Ly6C, *n* = 9–15 mice, either unpaired Student’s *t*-test or Mann–Whitney test. *P* = 0.001 (CD11b^+^ Ly6G^+^) and *P* = 0.0101 (CD11b^+^ Ly6G^+^). (*B*) Vascular superoxide formation oxidative fluorescence microtopography of aortic sections. Left: representative image of aortic sections showing lamina autofluorescence (green) and reactive oxygen species formation (red), scale bar = 50 µm. Right: densitometric analysis of vascular superoxide, normalized to control mice per experimental day. *n* = 6, unpaired Student’s *t*-test. *P* = 0.0275. (*C*) *Mcp1* (*P* < 0.001), *Vcam1* (*P* = 0.01), *Cxcl1* (*P* = 0.0), *Cxcl2* (ns), *Tnf* (*P*  *=*  *0.009*), *Nox2* (*P* = 0.005), *Il6* (*P*  *=*  *0.01*), *Il1β* (ns), *Stat3* (*P* = 0.002), *Rorc* (*P* = 0.02), *iNOS* (*P* = 0.005), and *Vegf-a* (*P* = 0.005) expression in the aorta of LysM-IL-6^OE^ normalized to control mice. Housekeeping gene: *Tbp*. *n* = 5–19, Mann–Whitney test or unpaired Student’s *t*-test. (*D*) Representative Western blot and statistical analyses for inducible NO synthase and endothelin-1 normalized to β-actin, *n* = 6–10, unpaired Student’s *t*-test. *P* = 0.0167 (inducible NO synthase) and *P* = 0.0071 (endothelin-1). (*E*) Immunohistochemical analysis of endothelin-1 in aortic sections. Left: representative images of endothelin-1 staining of aortic sections with positive and negative controls, scale bar = 50 µm. Right: quantification of the percentage of endothelin-1-positive area. The fatty tissue was excluded. *n* = 5–4, Mann–Whitney test. *P* = 0.02. (*F*) Endothelin-1 expression of human pulmonary arterial endothelial cell co-cultured with IL-6 and sIL-6R for 4 h (quantitative real-time PCR). *n* = 7, one-way ANOVA test. *P* = 0.045 (human pulmonary arterial endothelial cell vs. human pulmonary arterial endothelial cell co-cultured with IL-6 and sIL-6R 500pg) and *P* = 0.0102 (human pulmonary arterial endothelial cell co-cultured with interleukin-6 and sIL-6R 50pg vs. 500pg). Data are presented as mean ± SEM, and *P* values of <0.05 were considered significant and marked by asterisks (*P < 0.05; **P < 0.01; ***P < 0.001).

Flow cytometric analysis of CD11b^+^ cells in aorta showed that vascular dysfunction in LysM-IL-6^OE^ mice was accompanied by an increase in Ly6G^+^ Ly6C^+^ neutrophils and by Ly6G^−^ Lys6C^+^ monocytes/macrophages, but neither CX3CR1^+^ nor the CD115^+^ CD11b^+^ monocytes/macrophages, in the aortic vessel wall (*Figure 3A* and Supplementary material online, *Figure S5A* (gating strategy) and *B*). Total aortic CD11b^+^ cell numbers were not increased, but we noted a shift towards the Ly6G^+^ Ly6C^+^ neutrophils within this population (*Figure 3A*). The quantity of CD90.2^+^ T cells was increased in LysM-IL6^OE^ aortas, whereas no significant change in aortic CD4^+^ or CD8^+^ T cells was observed (see Supplementary material online, *Figure S5C* and *D*). In parallel to the described inflammation in the aortic vessel wall, increased ROS formation in the aortic vasculature was apparent (*Figure 3B*). In line, *Mcp1*, *VCAM1*, *Cxcl1*, *Tnf*, *Nox2*, *Il6*, *STAT3*, *Rorc*, *iNOS*, and *VEGFa* mRNA expression in the aortic vessel wall of LysM-IL-6OE mice was significantly increased (*Figure 3C*). Next to the increased protein expression of the inducible NO synthase (iNOS) in the LysM-IL-6^OE^ aortas contributing to increased vascular ROS formation (*Figure 3D*), ET-1, a potent vasoconstrictor and mediator of vascular dysfunction,^42^ was elevated in protein expression (*Figure 3D*) and histological analysis (*Figure 3E*). Stimulation of HPAECs with IL-6 and sIL-6R resulted in a significantly increased ET-1 expression (*Figure 3F*), suggesting that IL-6 directly triggers ET-1 expression in endothelial cells. This observation, in combination with the reported capacity of ET-1 to drive IL-6 expression in endothelial cells,^43^ may trigger a vascular dysfunction vicious circle. Of note, IL-6 synthesis in pulmonary endothelial cells isolated from LysM-IL-6^OE^ mice was increased compared with controls (see Supplementary material online, *Figure S6A*). Expression of the IL-6 receptor IL-6R was increased in the aortic tissue of LysM-IL6^OE^ mice, further suggesting that IL-6 directly acts on the vessel wall in our experimental mice (see Supplementary material online, *Figure S6B*).

Focusing on the role of ROS in this context, we applied the ROS scavengers SOD and catalase, respectively, over 90 min in the organ bath (see Supplementary material online, *Figure S7*) and found no relevant improving effect. Additionally, application of the ET-1 antagonist bosentan displayed no effect (see Supplementary material online, *Figure S7*). This indicated that both ROS and ET-1 do not function as acute drivers of vascular dysfunction in this mouse model, but rather contribute to the chronic effects of myeloid cell-derived IL-6 on the vasculature.

**Figure 4:**
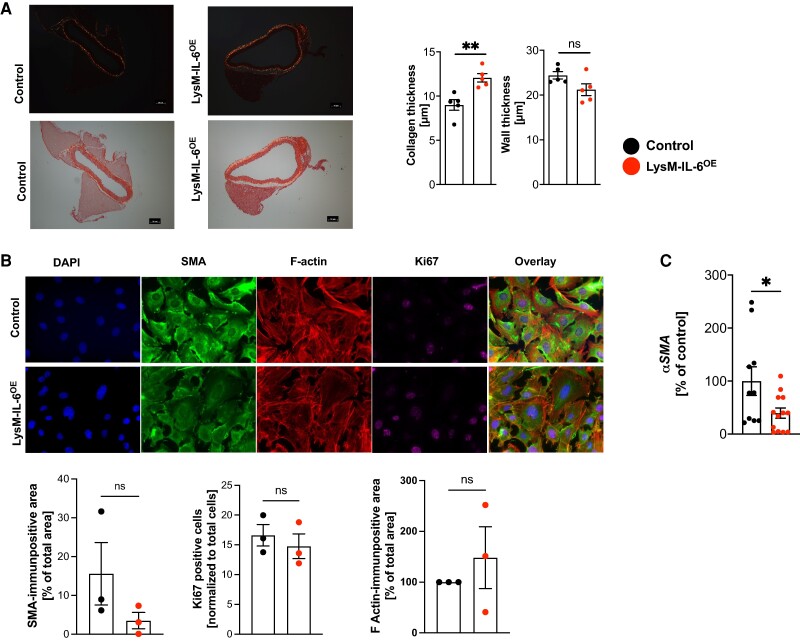
**Interleukin-6-induced vascular dysfunction is associated with an altered vascular smooth muscle cell phenotype and fibrosis formation.** (*A*) Collagen deposition in aortic sections. Sirius Red staining of aortic sections. Left: representative image of aortic sections (images without polarized light are shown below), scale bar = 50 µm. Right: aortic wall thickness and collagen thickness measurement at 10 different points/section with ImageJ software, *n* = 5, unpaired Student’s *t*-test. *P* = 0.0079 (collagen thickness). (*B*) Immunocytochemical analysis of vascular smooth muscle cell phenotype. Vascular smooth muscle cells were isolated from LysM-IL-6^OE^ mice and control mice and cultured. Cultured vascular smooth muscle cell were stained with smooth muscle alpha-actin, F-actin, Ki67, and DAPI (60×). Representative images are shown in the top row. Quantification is shown in the bottom row. *n* = 3 mice per group of two independent experiments. Single images of three biological replicates per mouse were analysed, and the mean values each mouse were compared, unpaired Student’s *t*-test. (*C*) Quantitative real-time PCR analysis for *alpha SMA* in LysM-IL-6^OE^ mice aortas (red) vs. control aortas. Housekeeping gene: *Gapdh*. *n* = 10–12 mice per group, unpaired Student’s *t*-test. *P* = 0.03. Data are presented as mean ± SEM, and *P* values of <0.05 were considered significant and marked by asterisks (*P < 0.05; **P < 0.01; ***P < 0.001).

In regard to the complex long-term vascular effects evoked by myeloid cell-derived IL-6 overexpression on the vasculature, we found that the amount of interstitial collagen thickness in the vascular wall of LysM-IL-6^OE^ mice was moderately increased, whilst the aortic wall thickness remained unchanged (*Figure 4A*). This is in line with the concept that vascular inflammation promotes vascular fibrosis.^44^ To investigate the mechanistic underpinnings, we examined cultured VSMCs isolated from LysM-IL-6^OE^ and control mice by immunocytochemistry (*Figure 4B*): here, no significant differences in proliferation (measured *via* Ki67) were apparent, although the presence of 10% serum in the medium may have masked minor changes. Expression of the mechanical stress marker F-actin did not differ between the VSMCs isolated from LysM-IL-6^OE^ and control mice. However, there was a trend in SMA to be less expressed in LysM-IL-6^OE^ VSMCs compared with controls (*Figure 4B*). This was corroborated in aortic tissue, where we found significantly less SMA mRNA expression in the LysM-IL-6^OE^ compared with control mice (*Figure 4C*). We found increased *matrix metallopeptidase-9* (*Mmp9*) mRNA levels in the LysM-IL-6^OE^ mice (see Supplementary material online, *Figure S8*). The mRNA levels of *Mmp2*, collagen (*Col1a1*, *Col1a2*, *Col3a1*), or vimentin did not differ significantly (see Supplementary material online, *Figure S8*).

### Interleukin-6 produced by bone - marrow-derived cells correlates with vascular dysfunction and systemic inflammation

**Figure 5:**
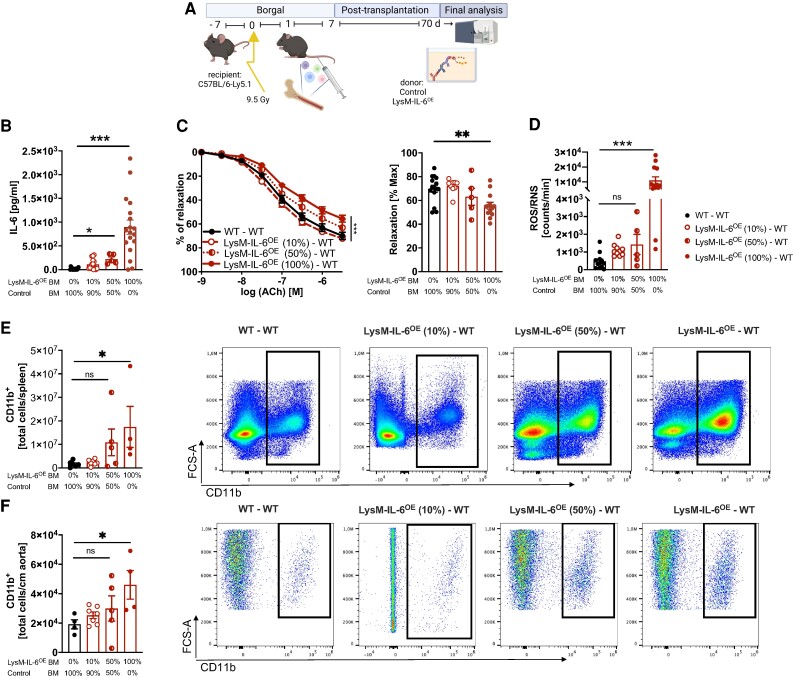
**Bone marrow and blood interleukin-6 levels correlate with systemic and vascular inflammation and dysfunction.** (*A*) Experimental approach of bone marrow transplantation. Schematic representation of the experimental design: different ratios of bone marrow isolated from LysM-IL-6^OE^ mice mixed with bone marrow from control mice (100, 50, and 10%) were transplanted into C57BL6-Ly5.1 mice. The transplantation scenarios included wild-type bone marrow → wild type (black circles), 100% LysM-IL-6^OE^ bone marrow → wild type (red circles completely filled with red colour), and mixed bone marrow consisting of 50% LysM-IL-6^OE^/50% wild type → wild type (half red circles) or 10% LysM-IL-6^OE^/90% wild type → wild type (red unfilled circles). Final analysis was conducted 70 days after bone marrow transfer. (*B*) Interleukin-6 levels in serum were measured by ELISA and Bioplex analysis in the bone marrow chimeric mice. *n* = 12–13, Kruskal–Wallis test with comparison of the mean of each column to the 100% control bone marrow column. *P* = 0.0245 (100% control bone marrow vs. 50% LysM-IL-6^OE^ bone marrow) and *P* < 0.0001 (100% control bone marrow vs. 100% LysM-IL-6^OE^ bone marrow). (*C*) Left: isometric tension studies of isolated aortic rings of bone marrow chimeric mice in response to acetylcholine. *n* = 5–16 mice/group, two-way ANOVA, Bonferroni’s multiple comparison. *P* < 0.0001 (comparison of the endpoint of the aortic relaxation curves of 100% control bone marrow vs. 100% LysM-IL-6^OE^ bone marrow). Right: comparison of the maximal relaxation of the aortic relaxation curves in response to acetylcholine shown on the left-hand side. *P* = 0.003 (comparison of the maximum relaxation of the aortic relaxation curves of 100% control bone marrow vs. 100% LysM-IL-6^OE^ bone marrow). (*D*) Reactive oxygen species/reactive nitrogen species measurement in blood after 20 min stimulation with phorbol 12,13-dibutyrate in bone marrow chimeric mice, *n* = 5–14, Kruskal–Wallis test with comparison of the mean of each column to the 100% control bone marrow column. *P* < 0.001 (100% control bone marrow vs. 100% LysM-IL-6^OE^ bone marrow). (*E*) Flow cytometric analysis of the CD11b^+^ cells in the spleen of bone marrow chimeric mice. *n* = 4–7 mice/group, Kruskal–Wallis test with comparison of the mean of each column to the 100% control bone marrow column. *P* = 0.0134 (100% control bone marrow vs. 100% LysM-IL-6^OE^ bone marrow). (*F*) Flow cytometric analysis of the CD11b^+^ cells in the aorta of bone marrow chimeric mice. *n* = 4–7 mice/group, Kruskal–Wallis test with comparison of the mean of each column to the 100% control bone marrow column. *P* = 0.0215 (100% control bone marrow vs. 100% LysM-IL-6^OE^ bone marrow). Data are presented as mean ± SEM, and *P* values of <0.05 were considered significant and marked by asterisks (*P < 0.05; **P < 0.01; ***P < 0.001).

To elucidate the contribution of myeloid cells derived from the BM, we generated chimeric mice transplanted with different ratios of BM isolated from LysM-IL-6^OE^ mice (100, 50, and 10%) mixed with BM of control mice up to 100% (*Figure 5A*). Interestingly, circulating IL-6 levels in plasma (*Figure 5B*), vascular endothelial dysfunction (*Figure 5C*), and oxidative stress levels in blood (*Figure 5D*) all correlated with the amount of LysM-IL-6^OE^ mice BM transferred and were most significantly pronounced in WT mice reconstituted with 100% LysM-IL-6^OE^ BM cells. Importantly, vascular function in WT mice transplanted with only 10% of LysM-IL-6^OE^ BM was unchanged to controls (*Figure 5C*), indicating a dose-dependent effect of IL-6 on systemic inflammation and vascular dysfunction. The amount of CD11b^+^ myeloid cells invading in the aortic vessel and spleen as markers of the IL-6-driven chronic systemic inflammation also correlated with the transferred amount of LysM-IL-6^OE^ mice BM (*Figure 5E* and *F*).

These findings show that vascular dysfunction and inflammation, as well as systemic inflammation and ROS formation, are driven by BM cell-derived IL-6 in a dose-dependent manner.

## Discussion

Our results highlight the crucial long-term impact of myeloid cell-derived IL-6 on the development of vascular inflammation and dysfunction and underline a clear dose-dependent effect.

For us as clinical scientists based in cardiology, this mouse model was generated with the main aim to decipher the role of myeloid cell-derived IL-6 in CVD. This is of high clinical relevance, as elevated IL-6 levels in patients with CVD (as also seen for example in obesity^45^) seem to predict a poorer outcome,^22,46^ underlining the need for a better understanding of IL-6 in CVD.

The well-established central role of IL-6 in systemic inflammation^47^ was confirmed in our mouse model. Additionally, our mouse model highlights the central role of IL-6 in inflammatory bowel disease (IBD).^15^ This is in line with the observation that patients with IBD have a higher risk of cardiovascular events compared with patients without IBD despite an unchanged incidence of conventional cardiovascular risk factors.^17^ Our mouse model also reflects the cardiovascular comorbidity of patients with IBD. One relevant factor of the cardiovascular comorbidity in IBD might be the chronic systemic inflammation.^17^ The microbiome, which is relevant in CVD development^48^ and IBD,^49^ might represent a further interconnecting factor.

To the best of our knowledge, this is the first report on the chronic effects of IL-6 on the vasculature in a mouse model. Elevated levels of IL-6, activated downstream of the cytokine IL-1β, are known to be associated with an increased rate of adverse cardiovascular events in patients with chronic atherosclerotic disease.^50,51^ The inflammation concept in CAD supports the idea to specifically target the IL-6 receptor pathways to prevent or attenuate CAD.^52^ Interleukin-6 was found in atherosclerotic plaques^53^ and shown to promote atherosclerotic lesion progression in mice.^54^ Recently, it has been demonstrated that treatment approaches of antagonizing IL-6 could reduce various biomarkers of systemic inflammation associated with the atherothrombotic process in patients with high atherosclerotic risk.^55,56^ In contrast, a potentially atheroprotective role of IL-6 has also been reported.^57,58^ This underscores that experimental results on the role of IL-6 in the cardiovascular system must be interpreted carefully.^22^

In our study, we detected that IL-6 induces vascular dysfunction systemically—in both large and small vessels. This was paralleled by an increased ROS formation, recapitulating findings by Wassmann *et al*.^59^ in mice treated with IL-6 over 18 days. In parallel, *Mcp1*, *Vcam1*, *Cxcl1 and 2*, *Tnf*, *iNOS*, *Nox2*, *Stat3*, and *Rorc*, key drivers and relevant components of vascular dysfunction and inflammation, were upregulated in the LysM-IL-6^OE^ aortas. The increased infiltration of myeloid cells into the aortic vessel wall and the increased vascular ROS formation detected in LysM-IL-6^OE^ mice are fully compatible with the activating effect of IL-6 on myeloid cells.^8,60^ In comparison with mice with AngII-induced vascular dysfunction (1 week of AngII treatment), the number of myeloid cells invading into the aortic vessel wall seemed higher in the AngII-treated mice.^61^ This suggests that it is necessary to differentiate between long-term inflammation, as seen in the LysM-IL-6^OE^ mouse model, and the short-term inflammation in the AngII model. However, since ROS scavengers as catalase could not attenuate vascular dysfunction when applied to LysM-IL-6^OE^ aortas *in vitro*, this indicates that not acute exposure to oxidative stress, but rather long-term impact of chronic IL-6-driven inflammation renders the vasculature dysfunctional.

We therefore focussed on these long-term effects: we found a moderately increased fibrosis in the aortic vessel walls of the LysM-IL-6^OE^ mice in line with the reported fibroblast stimulating effect of IL-6.^62^ Although this increase in the interstitial collagen thickness was significant, we were not faced with a major difference in size. This finding may pave the way for long-term IL-6-driven alterations of the vasculature and reflect an ongoing process. In any case, the increased mRNA expression of *MMP-9* is compatible with an increased collagen turnover.^63^ Further, we found an altered phenotype of VSMCs in the form of loss of contractile markers, suggesting a phenotypic switch towards a more synthetic phenotype.^64,65^ We therefore hypothesize that the vessels in the LysM-IL-6^OE^ mice are most likely in the process of an altered collagen formation. Nevertheless, they still provide an increased capacity to constrict upon stimulus. Taken together, these findings conclude that the vascular dysfunction initiated by myeloid cell-derived IL-6 overexpression involves the activation of multiple vascular cell types and release of downstream mediators and finally impacts the vascular collagen and VSMC phenotype.

Vascular dysfunction in LysM-IL-6^OE^ mice was associated with an altered VSMC phenotype but did not evoke blood pressure elevation and systemic hypertension. The nitric oxide release of iNOS, which was upregulated in the LysM-IL-6^OE^ aortas, could potentially blunt a potential blood pressure increase. The increased aortic ET-1 expression in the LysM-IL-6^OE^ mice is strengthened by reports that chronic endothelial ET-1 overexpression is associated with normal blood pressure^66^ (in contrast to short-term endothelial overexpression of ET-1 resulting in hypertension^67^). Interestingly, our data demonstrate IL-6 to be capable of driving vascular ET-1 expression. In total, this suggests that chronic IL-6 expression has the potential to trigger a vicious cycle of vascular dysfunction and inflammation. High expression of the IL-6 receptor in the aortic tissue in LysM-IL-6^OE^ mice suggests that IL-6 can directly impact the vasculature. This assumption is in line with data reported for human Endothelial cells (ECs).^68^ Further analysis has to follow on the exact IL-6 signalling in ECs and VSMCs under chronic IL-6 exposure. Here, the dose–response curve of IL-6 in the cardiovascular system must be kept in mind to determine at which IL-6 quantity chronic IL-6 exposure evokes changes in the cardiovascular system. The BM transfer experiments we performed suggest that the number of IL-6-producing myeloid cells in the BM correlates directly with systemic and vascular inflammation. Further studies on the impact of anti-IL6 treatment in this mouse model—and in a mouse model of vascular dysfunction—are needed.

The reported mouse model, as all genetic mouse models, certainly is not fully physiologic. However, extrapolating murine experimental knowledge from bench to bedside can raise necessary awareness that patients with elevated IL-6 levels (e.g. due to chronic IL-6-driven inflammatory diseases) are at risk to develop vascular inflammation. Since IL-6 levels correlate not only with systemic inflammation but also with vascular dysfunction and inflammation, it must be one treatment goal to keep the underlying inflammatory situation limited.

## Conclusion

Long-term myeloid cell-derived IL-6 expression evokes a chronic systemic inflammatory state with autoimmune colitis as a leading symptom. This is associated with significant vascular inflammation and dysfunction, ROS formation, elevated vascular ET-1 expression, alterations of the VSMCs, and increased collagen formation. Vascular dysfunction and ROS formation were driven by BM cell-derived IL-6 in a directly dose-dependent manner. Based on our findings, we conclude that long-term reduction in systemic IL-6 levels (e.g. by treating the existing autoimmune disease) is essential to reduce the associated vascular phenotype.

Further research focused on the complex impact of central cytokines as IL-6 on vascular regulation in (chronic) inflammatory diseases will lead to a better understanding of the vascular component in inflammatory diseases and new targets amenable for drug therapy.

## Lead author biography


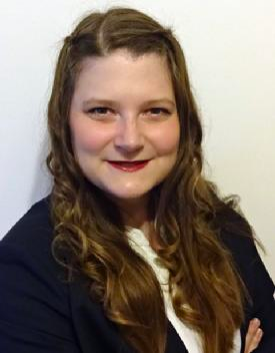


Tanja Knopp studied anthropology in Mainz, Germany, and focused on the impact of interleukin-6 on the vasculature and on the coagulation cascade during her thesis. As a post-doc in Bern, Switzerland, she is now working in the field of thrombosis and haemostasis.


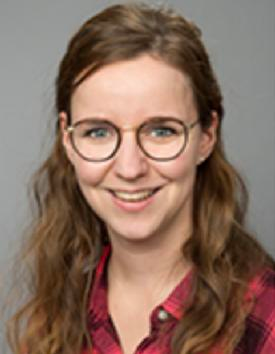


Rebecca Jung studied biology in Mainz, Germany. As a PhD candidate, she concentrated on the role of the cytokines IL-6 and IL-17A in vascular dysfunction development and on the cardiovascular comorbidity in the IL-17A-driven autoimmune skin disease psoriasis. She meanwhile switched from university to industry and continued working in the field of autoimmune diseases.

## Supplementary Material

oeae046_Supplementary_Data

## Data Availability

The data underlying this article will be shared on request to the corresponding author.

## References

[1] Fanola CL, Morrow DA, Cannon CP, Jarolim P, Lukas MA, Bode C, Hochman JS, Goodrich EL, Braunwald E, O'Donoghue ML. Interleukin-6 and the risk of adverse outcomes in patients after an acute coronary syndrome: observations from the SOLID-TIMI 52 (stabilization of plaque using darapladib-thrombolysis in myocardial infarction 52) trial. J Am Heart Assoc 2017;6:e005637. 29066436 10.1161/JAHA.117.005637 PMC5721825

[2] Gager GM, Biesinger B, Hofer F, Winter MP, Hengstenberg C, Jilma B, Eyileten C, Postula M, Lang IM, Siller-Matula JM. Interleukin-6 level is a powerful predictor of long-term cardiovascular mortality in patients with acute coronary syndrome. Vascul Pharmacol 2020;135:106806. 33035661 10.1016/j.vph.2020.106806

[3] Markousis-Mavrogenis G, Tromp J, Ouwerkerk W, Devalaraja M, Anker SD, Cleland JG, Dickstein K, Filippatos GS, van der Harst P, Lang CC, Metra M, Ng LL, Ponikowski P, Samani NJ, Zannad F, Zwinderman AH, Hillege HL, van Veldhuisen DJ, Kakkar R, Voors AA, van der Meer P. The clinical significance of interleukin-6 in heart failure: results from the BIOSTAT-CHF study. Eur J Heart Fail 2019;21:965–973. 31087601 10.1002/ejhf.1482

[4] Wainstein MV, Mossmann M, Araujo GN, Goncalves SC, Gravina GL, Sangalli M, Veadrigo F, Matte R, Reich R, Costa FG, Andrades M, da Silva AMV, Bertoluci MC. Elevated serum interleukin-6 is predictive of coronary artery disease in intermediate risk overweight patients referred for coronary angiography. Diabetol Metab Syndr 2017;9:67. 28878828 10.1186/s13098-017-0266-5 PMC5585915

[5] Held C, White HD, Stewart RAH, Budaj A, Cannon CP, Hochman JS, Koenig W, Siegbahn A, Steg PG, Soffer J, Weaver WD, Östlund O, Wallentin L; STABILITY Investigators. Inflammatory biomarkers interleukin-6 and C-reactive protein and outcomes in stable coronary heart disease: experiences from the STABILITY (stabilization of atherosclerotic plaque by initiation of darapladib therapy) trial. J Am Heart Assoc 2017;6:e005077. 29066452 10.1161/JAHA.116.005077 PMC5721818

[6] Korn T, Bettelli E, Oukka M, Kuchroo VK. IL-17 and Th17 cells. Annu Rev Immunol 2009;27:485–517. 19132915 10.1146/annurev.immunol.021908.132710

[7] Iwakura Y, Ishigame H, Saijo S, Nakae S. Functional specialization of interleukin-17 family members. Immunity 2011;34:149–162. 21349428 10.1016/j.immuni.2011.02.012

[8] Kaplanski G, Marin V, Montero-Julian F, Mantovani A, Farnarier C. IL-6: a regulator of the transition from neutrophil to monocyte recruitment during inflammation. Trends Immunol 2003;24:25–29. 12495721 10.1016/s1471-4906(02)00013-3

[9] Murphy K, Travers P, Walport M. Janeway´s immunobiology. Seventh Edition ed. New York: Garland Science, Taylor and Francis Group, LLC; 2008.

[10] Rose-John S . IL-6 trans-signaling via the soluble IL-6 receptor: importance for the pro-inflammatory activities of IL-6. Int J Biol Sci 2012;8:1237–1247. 23136552 10.7150/ijbs.4989 PMC3491447

[11] Hunter CA, Jones SA. IL-6 as a keystone cytokine in health and disease. Nat Immunol 2015;16:448–457. 25898198 10.1038/ni.3153

[12] Blauvelt A . IL-6 Differs from TNF-alpha: unpredicted clinical effects caused by IL-6 blockade in psoriasis. J Invest Dermatol 2017;137:541–542. 28235443 10.1016/j.jid.2016.11.022

[13] Choy EH, De Benedetti F, Takeuchi T, Hashizume M, John MR, Kishimoto T. Translating IL-6 biology into effective treatments. Nat Rev Rheumatol 2020;16:335–345. 32327746 10.1038/s41584-020-0419-z PMC7178926

[14] McHugh J . Systemic lupus erythematosus: B cell-derived IL-6 promotes disease. Nat Rev Rheumatol 2017;13:633. 10.1038/nrrheum.2017.163 28959044

[15] Schreiber S, Aden K, Bernardes JP, Conrad C, Tran F, Hoper H, Volk V, Mishra N, Blase JI, Nikolaus S, Bethge J, Kühbacher T, Röcken C, Chen M, Cottingham I, Petri N, Rasmussen BB, Lokau J, Lenk L, Garbers C, Feuerhake F, Rose-John S, Waetzig GH, Rosenstiel P. Therapeutic interleukin-6 trans-signaling inhibition by olamkicept (sgp130Fc) in patients with active inflammatory bowel disease. Gastroenterology 2021;160:2354–2366. e11. 33667488 10.1053/j.gastro.2021.02.062

[16] Mehta NN, Azfar RS, Shin DB, Neimann AL, Troxel AB, Gelfand JM. Patients with severe psoriasis are at increased risk of cardiovascular mortality: cohort study using the General Practice Research Database. Eur Heart J 2010;31:1000–1006. 20037179 10.1093/eurheartj/ehp567 PMC2894736

[17] Singh S, Kullo IJ, Pardi DS, Loftus EV Jr. Epidemiology, risk factors and management of cardiovascular diseases in IBD. Nat Rev Gastroenterol Hepatol 2015;12:26–35. 25446727 10.1038/nrgastro.2014.202

[18] Kahlenberg JM, Kaplan MJ. The interplay of inflammation and cardiovascular disease in systemic lupus erythematosus. Arthritis Res Ther 2011;13:203. 21371346 10.1186/ar3264 PMC3157642

[19] Hansildaar R, Vedder D, Baniaamam M, Tausche AK, Gerritsen M, Nurmohamed MT. Cardiovascular risk in inflammatory arthritis: rheumatoid arthritis and gout. Lancet Rheumatol 2021;3:e58–e70. 32904897 10.1016/S2665-9913(20)30221-6 PMC7462628

[20] Durante A, Bronzato S. The increased cardiovascular risk in patients affected by autoimmune diseases: review of the various manifestations. J Clin Med Res 2015;7:379–384. 25883699 10.14740/jocmr2122w PMC4394909

[21] Schrader LI, Kinzenbaw DA, Johnson AW, Faraci FM, Didion SP. IL-6 deficiency protects against angiotensin II induced endothelial dysfunction and hypertrophy. Arterioscler Thromb Vasc Biol 2007;27:2576–2581. 17962626 10.1161/ATVBAHA.107.153080

[22] Ridker PM, Rane M. Interleukin-6 signaling and anti-interleukin-6 therapeutics in cardiovascular disease. Circ Res 2021;128:1728–1746. 33998272 10.1161/CIRCRESAHA.121.319077

[23] Allocca M, Jovani M, Fiorino G, Schreiber S, Danese S. Anti-IL-6 treatment for inflammatory bowel diseases: next cytokine, next target. Curr Drug Targets 2013;14:1508–1521. 24102406 10.2174/13894501113146660224

[24] Clausen BE, Burkhardt C, Reith W, Renkawitz R, Forster I. Conditional gene targeting in macrophages and granulocytes using LysMcre mice. Transgenic Res 1999;8:265–277. 10621974 10.1023/a:1008942828960

[25] Munzel T, Giaid A, Kurz S, Stewart DJ, Harrison DG. Evidence for a role of endothelin 1 and protein kinase C in nitroglycerin tolerance. Proc Natl Acad Sci U S A 1995;92:5244–5248. 7539147 10.1073/pnas.92.11.5244 PMC41885

[26] Lagaud GJ, Randriamboavonjy V, Roul G, Stoclet JC, Andriantsitohaina R. Mechanism of Ca2+release and entry during contraction elicited by norepinephrine in rat resistance arteries. Am J Physiol 1999;276:H300–H308. 9887044 10.1152/ajpheart.1999.276.1.H300

[27] Randriamboavonjy V, Kyselova A, Elgheznawy A, Zukunft S, Wittig I, Fleming I. Calpain 1 cleaves and inactivates prostacyclin synthase in mesenteric arteries from diabetic mice. Basic Res Cardiol 2017;112:10. 28013348 10.1007/s00395-016-0596-8

[28] Daugherty A, Manning MW, Cassis LA. Angiotensin II promotes atherosclerotic lesions and aneurysms in apolipoprotein E-deficient mice. J Clin Invest 2000;105:1605–1612. 10841519 10.1172/JCI7818 PMC300846

[29] Karbach S, Croxford AL, Oelze M, Schuler R, Minwegen D, Wegner J, Koukes L, Yogev N, Nikolaev A, Reißig S, Ullmann A, Knorr M, Waldner M, Neurath MF, Li H, Wu Z, Brochhausen C, Scheller J, Rose-John S, Piotrowski C, Bechmann I, Radsak M, Wild P, Daiber A, von Stebut E, Wenzel P, Waisman A, Münzel T. Interleukin 17 drives vascular inflammation, endothelial dysfunction, and arterial hypertension in psoriasis-like skin disease. Arterioscler Thromb Vasc Biol 2014;34:2658–2668. 25341795 10.1161/ATVBAHA.114.304108

[30] Daiber A, August M, Baldus S, Wendt M, Oelze M, Sydow K, Kleschyov AL, Munzel T. Measurement of NAD(P)H oxidase-derived superoxide with the luminol analogue L-012. Free Radic Biol Med 2004;36:101–111. 14732294 10.1016/j.freeradbiomed.2003.10.012

[31] Oelze M, Daiber A, Brandes RP, Hortmann M, Wenzel P, Hink U, Schulz E, Mollnau H, von Sandersleben A, Kleschyov AL, Mülsch A, Li H, Förstermann U, Münzel T. Nebivolol inhibits superoxide formation by NADPH oxidase and endothelial dysfunction in angiotensin II-treated rats. Hypertension 2006;48:677–684. 16940222 10.1161/01.HYP.0000239207.82326.29

[32] Karbach SH, Schonfelder T, Brandao I, Wilms E, Hormann N, Jackel S, Schüler R, Finger S, Knorr M, Lagrange J, Brandt M, Waisman A, Kossmann S, Schäfer K, Münzel T, Reinhardt C, Wenzel P. Gut Microbiota promote angiotensin II-induced arterial hypertension and vascular dysfunction. J Am Heart Assoc 2016;5:e003698. 27577581 10.1161/JAHA.116.003698 PMC5079031

[33] Tang Y, Reissig S, Glasmacher E, Regen T, Wanke F, Nikolaev A, Gerlach K, Popp V, Karram K, Fantini MC, Schattenberg JM, Galle PR, Neurath MF, Weigmann B, Kurschus FC, Hövelmeyer N, Waisman A. Alternative splice forms of CYLD mediate ubiquitination of SMAD7 to prevent TGFB signaling and promote colitis. Gastroenterology 2019;156:692–707.e7. 30315770 10.1053/j.gastro.2018.10.023

[34] Cossarizza A, Chang HD, Radbruch A, Acs A, Adam D, Adam-Klages S, Agace WW, Aghaeepour N, Akdis M, Allez M, Almeida LN, Alvisi G, Anderson G, Andrä I, Annunziato F, Anselmo A, Bacher P, Baldari CT, Bari S, Barnaba V, Barros-Martins J, Battistini L, Bauer W, Baumgart S, Baumgarth N, Baumjohann D, Baying B, Bebawy M, Becher B, Beisker W, Benes V, Beyaert R, Blanco A, Boardman DA, Bogdan C, Borger JG, Borsellino G, Boulais PE, Bradford JA, Brenner D, Brinkman RR, Brooks AES, Busch DH, Büscher M, Bushnell TP, Calzetti F, Cameron G, Cammarata I, Cao X, Cardell SL, Casola S, Cassatella MA, Cavani A, Celada A, Chatenoud L, Chattopadhyay PK, Chow S, Christakou E, Čičin-Šain L, Clerici M, Colombo FS, Cook L, Cooke A, Cooper AM, Corbett AJ, Cosma A, Cosmi L, Coulie PG, Cumano A, Cvetkovic L, Dang VD, Dang-Heine C, Davey MS, Davies D, De Biasi S, Del Zotto G, Dela Cruz GV, Delacher M, Della Bella S, Dellabona P, Deniz G, Dessing M, Di Santo JP, Diefenbach A, Dieli F, Dolf A, Dörner T, Dress RJ, Dudziak D, Dustin M, Dutertre CA, Ebner F, Eckle SBG, Edinger M, Eede P, Ehrhardt GRA, Eich M, Engel P, Engelhardt B, Erdei A, Esser C, Everts B, Evrard M, Falk CS, Fehniger TA, Felipo-Benavent M, Ferry H, Feuerer M, Filby A, Filkor K, Fillatreau S, Follo M, Förster I, Foster J, Foulds GA, Frehse B, Frenette PS, Frischbutter S, Fritzsche W, Galbraith DW, Gangaev A, Garbi N, Gaudilliere B, Gazzinelli RT, Geginat J, Gerner W, Gherardin NA, Ghoreschi K, Gibellini L, Ginhoux F, Goda K, Godfrey DI, Goettlinger C, González-Navajas JM, Goodyear CS, Gori A, Grogan JL, Grummitt D, Grützkau A, Haftmann C, Hahn J, Hammad H, Hämmerling G, Hansmann L, Hansson G, Harpur CM, Hartmann S, Hauser A, Hauser AE, Haviland DL, Hedley D, Hernández DC, Herrera G, Herrmann M, Hess C, Höfer T, Hoffmann P, Hogquist K, Holland T, Höllt T, Holmdahl R, Hombrink P, Houston JP, Hoyer BF, Huang B, Huang FP, Huber JE, Huehn J, Hundemer M, Hunter CA, Hwang WYK, Iannone A, Ingelfinger F, Ivison SM, Jäck HM, Jani PK, Jávega B, Jonjic S, Kaiser T, Kalina T, Kamradt T, Kaufmann SHE, Keller B, Ketelaars SLC, Khalilnezhad A, Khan S, Kisielow J, Klenerman P, Knopf J, Koay HF, Kobow K, Kolls JK, Kong WT, Kopf M, Korn T, Kriegsmann K, Kristyanto H, Kroneis T, Krueger A, Kühne J, Kukat C, Kunkel D, Kunze-Schumacher H, Kurosaki T, Kurts C, Kvistborg P, Kwok I, Landry J, Lantz O, Lanuti P, LaRosa F, Lehuen A, LeibundGut-Landmann S, Leipold MD, Leung LYT, Levings MK, Lino AC, Liotta F, Litwin V, Liu Y, Ljunggren HG, Lohoff M, Lombardi G, Lopez L, López-Botet M, Lovett-Racke AE, Lubberts E, Luche H, Ludewig B, Lugli E, Lunemann S, Maecker HT, Maggi L, Maguire O, Mair F, Mair KH, Mantovani A, Manz RA, Marshall AJ, Martínez-Romero A, Martrus G, Marventano I, Maslinski W, Matarese G, Mattioli AV, Maueröder C, Mazzoni A, McCluskey J, McGrath M, McGuire HM, McInnes IB, Mei HE, Melchers F, Melzer S, Mielenz D, Miller SD, Mills KHG, Minderman H, Mjösberg J, Moore J, Moran B, Moretta L, Mosmann TR, Müller S, Multhoff G, Muñoz LE, Münz C, Nakayama T, Nasi M, Neumann K, Ng LG, Niedobitek A, Nourshargh S, Núñez G, O'Connor JE, Ochel A, Oja A, Ordonez D, Orfao A, Orlowski-Oliver E, Ouyang W, Oxenius A, Palankar R, Panse I, Pattanapanyasat K, Paulsen M, Pavlinic D, Penter L, Peterson P, Peth C, Petriz J, Piancone F, Pickl WF, Piconese S, Pinti M, Pockley AG, Podolska MJ, Poon Z, Pracht K, Prinz I, Pucillo CEM, Quataert SA, Quatrini L, Quinn KM, Radbruch H, Radstake TRDJ, Rahmig S, Rahn HP, Rajwa B, Ravichandran G, Raz Y, Rebhahn JA, Recktenwald D, Reimer D, Reis e Sousa C, Remmerswaal EBM, Richter L, Rico LG, Riddell A, Rieger AM, Robinson JP, Romagnani C, Rubartelli A, Ruland J, Saalmüller A, Saeys Y, Saito T, Sakaguchi S, Sala-de-Oyanguren F, Samstag Y, Sanderson S, Sandrock I, Santoni A, Sanz RB, Saresella M, Sautes-Fridman C, Sawitzki B, Schadt L, Scheffold A, Scherer HU, Schiemann M, Schildberg FA, Schimisky E, Schlitzer A, Schlosser J, Schmid S, Schmitt S, Schober K, Schraivogel D, Schuh W, Schüler T, Schulte R, Schulz AR, Schulz SR, Scottá C, Scott-Algara D, Sester DP, Shankey TV, Silva-Santos B, Simon AK, Sitnik KM, Sozzani S, Speiser DE, Spidlen J, Stahlberg A, Stall AM, Stanley N, Stark R, Stehle C, Steinmetz T, Stockinger H, Takahama Y, Takeda K, Tan L, Tárnok A, Tiegs G, Toldi G, Tornack J, Traggiai E, Trebak M, Tree TIM, Trotter J, Trowsdale J, Tsoumakidou M, Ulrich H, Urbanczyk S, van de Veen W, van den Broek M, van der Pol E, Van Gassen S, Van Isterdael G, van Lier RAW, Veldhoen M, Vento-Asturias S, Vieira P, Voehringer D, Volk HD, von Borstel A, von Volkmann K, Waisman A, Walker RV, Wallace PK, Wang SA, Wang XM, Ward MD, Ward-Hartstonge KA, Warnatz K, Warnes G, Warth S, Waskow C, Watson JV, Watzl C, Wegener L, Weisenburger T, Wiedemann A, Wienands J, Wilharm A, Wilkinson RJ, Willimsky G, Wing JB, Winkelmann R, Winkler TH, Wirz OF, Wong A, Wurst P, Yang JHM, Yang J, Yazdanbakhsh M, Yu L, Yue A, Zhang H, Zhao Y, Ziegler SM, Zielinski C, Zimmermann J, Zychlinsky A. Guidelines for the use of flow cytometry and cell sorting in immunological studies (second edition). Eur J Immunol 2019;49:1457–1973. 31633216 10.1002/eji.201970107 PMC7350392

[35] Bochenek ML, Rosinus NS, Lankeit M, Hobohm L, Bremmer F, Schutz E, Klok FA, Horke S, Wiedenroth CB, Münzel T, Lang IM, Mayer E, Konstantinides S, Schäfer K. From thrombosis to fibrosis in chronic thromboembolic pulmonary hypertension. Thromb Haemost 2017;117:769–783. 28150849 10.1160/TH16-10-0790

[36] Gogiraju R, Renner L, Bochenek ML, Zifkos K, Molitor M, Danckwardt S, Wenzel P, Münzel T, Konstantinides S, Schäfer K. Arginase-1 deletion in erythrocytes promotes vascular calcification via enhanced GSNOR (S-nitrosoglutathione reductase) expression and NO signaling in smooth muscle cells. Arterioscler Thromb Vasc Biol 2022;42:e291–e310. 36252109 10.1161/ATVBAHA.122.318338

[37] Feldman AT, Wolfe D. Tissue processing and hematoxylin and eosin staining. Methods Mol Biol 2014;1180:31–43. 25015141 10.1007/978-1-4939-1050-2_3

[38] Oelze M, Knorr M, Kroller-Schon S, Kossmann S, Gottschlich A, Rummler R, Schuff A, Daub S, Doppler C, Kleinert H, Gori T, Daiber A, Münzel T. Chronic therapy with isosorbide-5-mononitrate causes endothelial dysfunction, oxidative stress, and a marked increase in vascular endothelin-1 expression. Eur Heart J 2013;34:3206–3216. 22555214 10.1093/eurheartj/ehs100

[39] Livak KJ, Schmittgen TD. Analysis of relative gene expression data using real-time quantitative PCR and the 2(-Delta Delta C(T)) method. Methods 2001;25:402–408. 11846609 10.1006/meth.2001.1262

[40] Bosmann M, Russkamp NF, Ward PA. Fingerprinting of the TLR4-induced acute inflammatory response. Exp Mol Pathol 2012;93:319–323. 22981705 10.1016/j.yexmp.2012.08.006 PMC3518574

[41] Klionsky DJ, Abdel-Aziz AK, Abdelfatah S, Abdellatif M, Abdoli A, Abel S, Abeliovich H, Abildgaard MH, Abudu YP, Acevedo-Arozena A, Adamopoulos IE, Adeli K, Adolph TE, Adornetto A, Aflaki E, Agam G, Agarwal A, Aggarwal BB, Agnello M, Agostinis P, Agrewala JN, Agrotis A, Aguilar PV, Ahmad ST, Ahmed ZM, Ahumada-Castro U, Aits S, Aizawa S, Akkoc Y, Akoumianaki T, Akpinar HA, Al-Abd AM, Al-Akra L, Al-Gharaibeh A, Alaoui-Jamali MA, Alberti S, Alcocer-Gómez E, Alessandri C, Ali M, Alim Al-Bari MA, Aliwaini S, Alizadeh J, Almacellas E, Almasan A, Alonso A, Alonso GD, Altan-Bonnet N, Altieri DC, Álvarez ÉMC, Alves S, Alves da Costa C, Alzaharna MM, Amadio M, Amantini C, Amaral C, Ambrosio S, Amer AO, Ammanathan V, An Z, Andersen SU, Andrabi SA, Andrade-Silva M, Andres AM, Angelini S, Ann D, Anozie UC, Ansari MY, Antas P, Antebi A, Antón Z, Anwar T, Apetoh L, Apostolova N, Araki T, Araki Y, Arasaki K, Araújo WL, Araya J, Arden C, Arévalo MA, Arguelles S, Arias E, Arikkath J, Arimoto H, Ariosa AR, Armstrong-James D, Arnauné-Pelloquin L, Aroca A, Arroyo DS, Arsov I, Artero R, Asaro DML, Aschner M, Ashrafizadeh M, Ashur-Fabian O, Atanasov AG, Au AK, Auberger P, Auner HW, Aurelian L, Autelli R, Avagliano L, Ávalos Y, Aveic S, Aveleira CA, Avin-Wittenberg T, Aydin Y, Ayton S, Ayyadevara S, Azzopardi M, Baba M, Backer JM, Backues SK, Bae DH, Bae ON, Bae SH, Baehrecke EH, Baek A, Baek SH, Baek SH, Bagetta G, Bagniewska-Zadworna A, Bai H, Bai J, Bai X, Bai Y, Bairagi N, Baksi S, Balbi T, Baldari CT, Balduini W, Ballabio A, Ballester M, Balazadeh S, Balzan R, Bandopadhyay R, Banerjee S, Banerjee S, Bánréti Á, Bao Y, Baptista MS, Baracca A, Barbati C, Bargiela A, Barilà D, Barlow PG, Barmada SJ, Barreiro E, Barreto GE, Bartek J, Bartel B, Bartolome A, Barve GR, Basagoudanavar SH, Bassham DC, Bast RC Jr, Basu A, Batoko H, Batten I, Baulieu EE, Baumgarner BL, Bayry J, Beale R, Beau I, Beaumatin F, Bechara LRG, Beck GR Jr, Beers MF, Begun J, Behrends C, Behrens GMN, Bei R, Bejarano E, Bel S, Behl C, Belaid A, Belgareh-Touzé N, Bellarosa C, Belleudi F, Belló Pérez M, Bello-Morales R, Beltran JSO, Beltran S, Benbrook DM, Bendorius M, Benitez BA, Benito-Cuesta I, Bensalem J, Berchtold MW, Berezowska S, Bergamaschi D, Bergami M, Bergmann A, Berliocchi L, Berlioz-Torrent C, Bernard A, Berthoux L, Besirli CG, Besteiro S, Betin VM, Beyaert R, Bezbradica JS, Bhaskar K, Bhatia-Kissova I, Bhattacharya R, Bhattacharya S, Bhattacharyya S, Bhuiyan MS, Bhutia SK, Bi L, Bi X, Biden TJ, Bijian K, Billes VA, Binart N, Bincoletto C, Birgisdottir AB, Bjorkoy G, Blanco G, Blas-Garcia A, Blasiak J, Blomgran R, Blomgren K, Blum JS, Boada-Romero E, Boban M, Boesze-Battaglia K, Boeuf P, Boland B, Bomont P, Bonaldo P, Bonam SR, Bonfili L, Bonifacino JS, Boone BA, Bootman MD, Bordi M, Borner C, Bornhauser BC, Borthakur G, Bosch J, Bose S, Botana LM, Botas J, Boulanger CM, Boulton ME, Bourdenx M, Bourgeois B, Bourke NM, Bousquet G, Boya P, Bozhkov PV, Bozi LHM, Bozkurt TO, Brackney DE, Brandts CH, Braun RJ, Braus GH, Bravo-Sagua R, Bravo-San Pedro JM, Brest P, Bringer MA, Briones-Herrera A, Broaddus VC, Brodersen P, Brodsky JL, Brody SL, Bronson PG, Bronstein JM, Brown CN, Brown RE, Brum PC, Brumell JH, Brunetti-Pierri N, Bruno D, Bryson-Richardson RJ, Bucci C, Buchrieser C, Bueno M, Buitrago-Molina LE, Buraschi S, Buch S, Buchan JR, Buckingham EM, Budak H, Budini M, Bultynck G, Burada F, Burgoyne JR, Burón MI, Bustos V, Büttner S, Butturini E, Byrd A, Cabas I, Cabrera-Benitez S, Cadwell K, Cai J, Cai L, Cai Q, Cairó M, Calbet JA, Caldwell GA, Caldwell KA, Call JA, Calvani R, Calvo AC, Calvo-Rubio Barrera M, Camara NO, Camonis JH, Camougrand N, Campanella M, Campbell EM, Campbell-Valois FX, Campello S, Campesi I, Campos JC, Camuzard O, Cancino J, Candido de Almeida D, Canesi L, Caniggia I, Canonico B, Cantí C, Cao B, Caraglia M, Caramés B, Carchman EH, Cardenal-Muñoz E, Cardenas C, Cardenas L, Cardoso SM, Carew JS, Carle GF, Carleton G, Carloni S, Carmona-Gutierrez D, Carneiro LA, Carnevali O, Carosi JM, Carra S, Carrier A, Carrier L, Carroll B, Carter AB, Carvalho AN, Casanova M, Casas C, Casas J, Cassioli C, Castillo EF, Castillo K, Castillo-Lluva S, Castoldi F, Castori M, Castro AF, Castro-Caldas M, Castro-Hernandez J, Castro-Obregon S, Catz SD, Cavadas C, Cavaliere F, Cavallini G, Cavinato M, Cayuela ML, Cebollada Rica P, Cecarini V, Cecconi F, Cechowska-Pasko M, Cenci S, Ceperuelo-Mallafré V, Cerqueira JJ, Cerutti JM, Cervia D, Cetintas VB, Cetrullo S, Chae HJ, Chagin AS, Chai CY, Chakrabarti G, Chakrabarti O, Chakraborty T, Chakraborty T, Chami M, Chamilos G, Chan DW, Chan EYW, Chan ED, Chan HYE, Chan HH, Chan H, Chan MTV, Chan YS, Chandra PK, Chang CP, Chang C, Chang HC, Chang K, Chao J, Chapman T, Charlet-Berguerand N, Chatterjee S, Chaube SK, Chaudhary A, Chauhan S, Chaum E, Checler F, Cheetham ME, Chen CS, Chen GC, Chen JF, Chen LL, Chen L, Chen L, Chen M, Chen MK, Chen N, Chen Q, Chen RH, Chen S, Chen W, Chen W, Chen XM, Chen XW, Chen X, Chen Y, Chen YG, Chen Y, Chen Y, Chen YJ, Chen YQ, Chen ZS, Chen Z, Chen ZH, Chen ZJ, Chen Z, Cheng H, Cheng J, Cheng SY, Cheng W, Cheng X, Cheng XT, Cheng Y, Cheng Z, Chen Z, Cheong H, Cheong JK, Chernyak BV, Cherry S, Cheung CFR, Cheung CHA, Cheung KH, Chevet E, Chi RJ, Chiang AKS, Chiaradonna F, Chiarelli R, Chiariello M, Chica N, Chiocca S, Chiong M, Chiou SH, Chiramel AI, Chiurchiù V, Cho DH, Choe SK, Choi AMK, Choi ME, Choudhury KR, Chow NS, Chu CT, Chua JP, Chua JJE, Chung H, Chung KP, Chung S, Chung SH, Chung YL, Cianfanelli V, Ciechomska IA, Cifuentes M, Cinque L, Cirak S, Cirone M, Clague MJ, Clarke R, Clementi E, Coccia EM, Codogno P, Cohen E, Cohen MM, Colasanti T, Colasuonno F, Colbert RA, Colell A, Čolić M, Coll NS, Collins MO, Colombo MI, Colón-Ramos DA, Combaret L, Comincini S, Cominetti MR, Consiglio A, Conte A, Conti F, Contu VR, Cookson MR, Coombs KM, Coppens I, Corasaniti MT, Corkery DP, Cordes N, Cortese K, Costa MDC, Costantino S, Costelli P, Coto-Montes A, Crack PJ, Crespo JL, Criollo A, Crippa V, Cristofani R, Csizmadia T, Cuadrado A, Cui B, Cui J, Cui Y, Cui Y, Culetto E, Cumino AC, Cybulsky AV, Czaja MJ, Czuczwar SJ, D'Adamo S, D'Amelio M, D'Arcangelo D, D'Lugos AC, D'Orazi G, da Silva JA, Dafsari HS, Dagda RK, Dagdas Y, Daglia M, Dai X, Dai Y, Dai Y, Dal Col J, Dalhaimer P, Dalla Valle L, Dallenga T, Dalmasso G, Damme M, Dando I, Dantuma NP, Darling AL, Das H, Dasarathy S, Dasari SK, Dash S, Daumke O, Dauphinee AN, Davies JS, Dávila VA, Davis RJ, Davis T, Dayalan Naidu S, De Amicis F, De Bosscher K, De Felice F, De Franceschi L, De Leonibus C, de Mattos Barbosa MG, De Meyer GRY, De Milito A, De Nunzio C, De Palma C, De Santi M, De Virgilio C, De Zio D, Debnath J, DeBosch BJ, Decuypere JP, Deehan MA, Deflorian G, DeGregori J, Dehay B, Del Rio G, Delaney JR, Delbridge LMD, Delorme-Axford E, Delpino MV, Demarchi F, Dembitz V, Demers ND, Deng H, Deng Z, Dengjel J, Dent P, Denton D, DePamphilis ML, Der CJ, Deretic V, Descoteaux A, Devis L, Devkota S, Devuyst O, Dewson G, Dharmasivam M, Dhiman R, di Bernardo D, Di Cristina M, Di Domenico F, Di Fazio P, Di Fonzo A, Di Guardo G, Di Guglielmo GM, Di Leo L, Di Malta C, Di Nardo A, Di Rienzo M, Di Sano F, Diallinas G, Diao J, Diaz-Araya G, Díaz-Laviada I, Dickinson JM, Diederich M, Dieudé M, Dikic I, Ding S, Ding WX, Dini L, Dinić J, Dinic M, Dinkova-Kostova AT, Dionne MS, Distler JHW, Diwan A, Dixon IMC, Djavaheri-Mergny M, Dobrinski I, Dobrovinskaya O, Dobrowolski R, Dobson RCJ, Đokić J, Dokmeci Emre S, Donadelli M, Dong B, Dong X, Dong Z, Dorn Ii GW, Dotsch V, Dou H, Dou J, Dowaidar M, Dridi S, Drucker L, Du A, Du C, Du G, Du HN, Du LL, du Toit A, Duan SB, Duan X, Duarte SP, Dubrovska A, Dunlop EA, Dupont N, Durán RV, Dwarakanath BS, Dyshlovoy SA, Ebrahimi-Fakhari D, Eckhart L, Edelstein CL, Efferth T, Eftekharpour E, Eichinger L, Eid N, Eisenberg T, Eissa NT, Eissa S, Ejarque M, El Andaloussi A, El-Hage N, El-Naggar S, Eleuteri AM, El-Shafey ES, Elgendy M, Eliopoulos AG, Elizalde MM, Elks PM, Elsasser HP, Elsherbiny ES, Emerling BM, Emre NCT, Eng CH, Engedal N, Engelbrecht AM, Engelsen AST, Enserink JM, Escalante R, Esclatine A, Escobar-Henriques M, Eskelinen EL, Espert L, Eusebio MO, Fabrias G, Fabrizi C, Facchiano A, Facchiano F, Fadeel B, Fader C, Faesen AC, Fairlie WD, Falcó A, Falkenburger BH, Fan D, Fan J, Fan Y, Fang EF, Fang Y, Fang Y, Fanto M, Farfel-Becker T, Faure M, Fazeli G, Fedele AO, Feldman AM, Feng D, Feng J, Feng L, Feng Y, Feng Y, Feng W, Fenz Araujo T, Ferguson TA, Fernández ÁF, Fernandez-Checa JC, Fernández-Veledo S, Fernie AR, Ferrante AW Jr, Ferraresi A, Ferrari MF, Ferreira JCB, Ferro-Novick S, Figueras A, Filadi R, Filigheddu N, Filippi-Chiela E, Filomeni G, Fimia GM, Fineschi V, Finetti F, Finkbeiner S, Fisher EA, Fisher PB, Flamigni F, Fliesler SJ, Flo TH, Florance I, Florey O, Florio T, Fodor E, Follo C, Fon EA, Forlino A, Fornai F, Fortini P, Fracassi A, Fraldi A, Franco B, Franco R, Franconi F, Frankel LB, Friedman SL, Fröhlich LF, Frühbeck G, Fuentes JM, Fujiki Y, Fujita N, Fujiwara Y, Fukuda M, Fulda S, Furic L, Furuya N, Fusco C, Gack MU, Gaffke L, Galadari S, Galasso A, Galindo MF, Gallolu Kankanamalage S, Galluzzi L, Galy V, Gammoh N, Gan B, Ganley IG, Gao F, Gao H, Gao M, Gao P, Gao SJ, Gao W, Gao X, Garcera A, Garcia MN, Garcia VE, García-Del Portillo F, Garcia-Escudero V, Garcia-Garcia A, Garcia-Macia M, García-Moreno D, Garcia-Ruiz C, García-Sanz P, Garg AD, Gargini R, Garofalo T, Garry RF, Gassen NC, Gatica D, Ge L, Ge W, Geiss-Friedlander R, Gelfi C, Genschik P, Gentle IE, Gerbino V, Gerhardt C, Germain K, Germain M, Gewirtz DA, Ghasemipour Afshar E, Ghavami S, Ghigo A, Ghosh M, Giamas G, Giampietri C, Giatromanolaki A, Gibson GE, Gibson SB, Ginet V, Giniger E, Giorgi C, Girao H, Girardin SE, Giridharan M, Giuliano S, Giulivi C, Giuriato S, Giustiniani J, Gluschko A, Goder V, Goginashvili A, Golab J, Goldstone DC, Golebiewska A, Gomes LR, Gomez R, Gómez-Sánchez R, Gomez-Puerto MC, Gomez-Sintes R, Gong Q, Goni FM, González-Gallego J, Gonzalez-Hernandez T, Gonzalez-Polo RA, Gonzalez-Reyes JA, González-Rodríguez P, Goping IS, Gorbatyuk MS, Gorbunov NV, Görgülü K, Gorojod RM, Gorski SM, Goruppi S, Gotor C, Gottlieb RA, Gozes I, Gozuacik D, Graef M, Gräler MH, Granatiero V, Grasso D, Gray JP, Green DR, Greenhough A, Gregory SL, Griffin EF, Grinstaff MW, Gros F, Grose C, Gross AS, Gruber F, Grumati P, Grune T, Gu X, Guan JL, Guardia CM, Guda K, Guerra F, Guerri C, Guha P, Guillén C, Gujar S, Gukovskaya A, Gukovsky I, Gunst J, Günther A, Guntur AR, Guo C, Guo C, Guo H, Guo LW, Guo M, Gupta P, Gupta SK, Gupta S, Gupta VB, Gupta V, Gustafsson AB, Gutterman DD, Haapasalo A, Haber JE, Hać A, Hadano S, Hafrén AJ, Haidar M, Hall BS, Halldén G, Hamacher-Brady A, Hamann A, Hamasaki M, Han W, Hansen M, Hanson PI, Hao Z, Harada M, Harhaji-Trajkovic L, Hariharan N, Haroon N, Harris J, Hasegawa T, Hasima Nagoor N, Haspel JA, Haucke V, Hawkins WD, Hay BA, Haynes CM, Hayrabedyan SB, Hays TS, He C, He Q, He RR, He YW, He YY, Heakal Y, Heberle AM, Hejtmancik JF, Helgason GV, Henkel V, Herb M, Hergovich A, Herman-Antosiewicz A, Hernández A, Hernandez C, Hernandez-Diaz S, Hernandez-Gea V, Herpin A, Herreros J, Hervás JH, Hesselson D, Hetz C, Heussler VT, Higuchi Y, Hilfiker S, Hill JA, Hlavacek WS, Ho EA, Ho IHT, Ho PW, Ho SL, Ho WY, Hobbs GA, Hochstrasser M, Hoet PHM, Hofius D, Hofman P, Höhn A, Holmberg CI, Hombrebueno JR, Yi-Ren Hong CH, Hooper LV, Hoppe T, Horos R, Hoshida Y, Hsin IL, Hsu HY, Hu B, Hu D, Hu LF, Hu MC, Hu R, Hu W, Hu YC, Hu ZW, Hua F, Hua J, Hua Y, Huan C, Huang C, Huang C, Huang C, Huang C, Huang H, Huang K, Huang MLH, Huang R, Huang S, Huang T, Huang X, Huang YJ, Huber TB, Hubert V, Hubner CA, Hughes SM, Hughes WE, Humbert M, Hummer G, Hurley JH, Hussain S, Hussain S, Hussey PJ, Hutabarat M, Hwang HY, Hwang S, Ieni A, Ikeda F, Imagawa Y, Imai Y, Imbriano C, Imoto M, Inman DM, Inoki K, Iovanna J, Iozzo RV, Ippolito G, Irazoqui JE, Iribarren P, Ishaq M, Ishikawa M, Ishimwe N, Isidoro C, Ismail N, Issazadeh-Navikas S, Itakura E, Ito D, Ivankovic D, Ivanova S, Iyer AKV, Izquierdo JM, Izumi M, Jäättelä M, Jabir MS, Jackson WT, Jacobo-Herrera N, Jacomin AC, Jacquin E, Jadiya P, Jaeschke H, Jagannath C, Jakobi AJ, Jakobsson J, Janji B, Jansen-Dürr P, Jansson PJ, Jantsch J, Januszewski S, Jassey A, Jean S, Jeltsch-David H, Jendelova P, Jenny A, Jensen TE, Jessen N, Jewell JL, Ji J, Jia L, Jia R, Jiang L, Jiang Q, Jiang R, Jiang T, Jiang X, Jiang Y, Jimenez-Sanchez M, Jin EJ, Jin F, Jin H, Jin L, Jin L, Jin M, Jin S, Jo EK, Joffre C, Johansen T, Johnson GVW, Johnston SA, Jokitalo E, Jolly MK, Joosten LAB, Jordan J, Joseph B, Ju D, Ju JS, Ju J, Juárez E, Judith D, Juhász G, Jun Y, Jung CH, Jung SC, Jung YK, Jungbluth H, Jungverdorben J, Just S, Kaarniranta K, Kaasik A, Kabuta T, Kaganovich D, Kahana A, Kain R, Kajimura S, Kalamvoki M, Kalia M, Kalinowski DS, Kaludercic N, Kalvari I, Kaminska J, Kaminskyy VO, Kanamori H, Kanasaki K, Kang C, Kang R, Kang SS, Kaniyappan S, Kanki T, Kanneganti TD, Kanthasamy AG, Kanthasamy A, Kantorow M, Kapuy O, Karamouzis MV, Karim MR, Karmakar P, Katare RG, Kato M, Kaufmann SHE, Kauppinen A, Kaushal GP, Kaushik S, Kawasaki K, Kazan K, Ke PY, Keating DJ, Keber U, Kehrl JH, Keller KE, Keller CW, Kemper JK, Kenific CM, Kepp O, Kermorgant S, Kern A, Ketteler R, Keulers TG, Khalfin B, Khalil H, Khambu B, Khan SY, Khandelwal VKM, Khandia R, Kho W, Khobrekar NV, Khuansuwan S, Khundadze M, Killackey SA, Kim D, Kim DR, Kim DH, Kim DE, Kim EY, Kim EK, Kim HR, Kim HS, Hyung-Ryong K, Kim JH, Kim JK, Kim JH, Kim J, Kim JH, Kim KI, Kim PK, Kim SJ, Kimball SR, Kimchi A, Kimmelman AC, Kimura T, King MA, Kinghorn KJ, Kinsey CG, Kirkin V, Kirshenbaum LA, Kiselev SL, Kishi S, Kitamoto K, Kitaoka Y, Kitazato K, Kitsis RN, Kittler JT, Kjaerulff O, Klein PS, Klopstock T, Klucken J, Knævelsrud H, Knorr RL, Ko BCB, Ko F, Ko JL, Kobayashi H, Kobayashi S, Koch I, Koch JC, Koenig U, Kögel D, Koh YH, Koike M, Kohlwein SD, Kocaturk NM, Komatsu M, König J, Kono T, Kopp BT, Korcsmaros T, Korkmaz G, Korolchuk VI, Korsnes MS, Koskela A, Kota J, Kotake Y, Kotler ML, Kou Y, Koukourakis MI, Koustas E, Kovacs AL, Kovács T, Koya D, Kozako T, Kraft C, Krainc D, Krämer H, Krasnodembskaya AD, Kretz-Remy C, Kroemer G, Ktistakis NT, Kuchitsu K, Kuenen S, Kuerschner L, Kukar T, Kumar A, Kumar A, Kumar D, Kumar D, Kumar S, Kume S, Kumsta C, Kundu CN, Kundu M, Kunnumakkara AB, Kurgan L, Kutateladze TG, Kutlu O, Kwak S, Kwon HJ, Kwon TK, Kwon YT, Kyrmizi I, La Spada A, Labonté P, Ladoire S, Laface I, Lafont F, Lagace DC, Lahiri V, Lai Z, Laird AS, Lakkaraju A, Lamark T, Lan SH, Landajuela A, Lane DJR, Lane JD, Lang CH, Lange C, Langel Ü, Langer R, Lapaquette P, Laporte J, LaRusso NF, Lastres-Becker I, Lau WCY, Laurie GW, Lavandero S, Law BYK, Law HK, Layfield R, Le W, Le Stunff H, Leary AY, Lebrun JJ, Leck LYW, Leduc-Gaudet JP, Lee C, Lee CP, Lee DH, Lee EB, Lee EF, Lee GM, Lee HJ, Lee HK, Lee JM, Lee JS, Lee JA, Lee JY, Lee JH, Lee M, Lee MG, Lee MJ, Lee MS, Lee SY, Lee SJ, Lee SY, Lee SB, Lee WH, Lee YR, Lee YH, Lee Y, Lefebvre C, Legouis R, Lei YL, Lei Y, Leikin S, Leitinger G, Lemus L, Leng S, Lenoir O, Lenz G, Lenz HJ, Lenzi P, León Y, Leopoldino AM, Leschczyk C, Leskelä S, Letellier E, Leung CT, Leung PS, Leventhal JS, Levine B, Lewis PA, Ley K, Li B, Li DQ, Li J, Li J, Li J, Li K, Li L, Li M, Li M, Li M, Li M, Li M, Li PL, Li MQ, Li Q, Li S, Li T, Li W, Li W, Li X, Li YP, Li Y, Li Z, Li Z, Li Z, Lian J, Liang C, Liang Q, Liang W, Liang Y, Liang Y, Liao G, Liao L, Liao M, Liao YF, Librizzi M, Lie PPY, Lilly MA, Lim HJ, Lima TRR, Limana F, Lin C, Lin CW, Lin DS, Lin FC, Lin JD, Lin KM, Lin KH, Lin LT, Lin PH, Lin Q, Lin S, Lin SJ, Lin W, Lin X, Lin YX, Lin YS, Linden R, Lindner P, Ling SC, Lingor P, Linnemann AK, Liou YC, Lipinski MM, Lipovšek S, Lira VA, Lisiak N, Liton PB, Liu C, Liu CH, Liu CF, Liu CH, Liu F, Liu H, Liu HS, Liu HF, Liu H, Liu J, Liu J, Liu J, Liu L, Liu L, Liu M, Liu Q, Liu W, Liu W, Liu XH, Liu X, Liu X, Liu X, Liu X, Liu Y, Liu Y, Liu Y, Liu Y, Liu Y, Livingston JA, Lizard G, Lizcano JM, Ljubojevic-Holzer S, LLeonart ME, Llobet-Navàs D, Llorente A, Lo CH, Lobato-Márquez D, Long Q, Long YC, Loos B, Loos JA, López MG, López-Doménech G, López-Guerrero JA, López-Jiménez AT, López-Pérez Ó, López-Valero I, Lorenowicz MJ, Lorente M, Lorincz P, Lossi L, Lotersztajn S, Lovat PE, Lovell JF, Lovy A, Lőw P, Lu G, Lu H, Lu JH, Lu JJ, Lu M, Lu S, Luciani A, Lucocq JM, Ludovico P, Luftig MA, Luhr M, Luis-Ravelo D, Lum JJ, Luna-Dulcey L, Lund AH, Lund VK, Lünemann JD, Lüningschrör P, Luo H, Luo R, Luo S, Luo Z, Luparello C, Lüscher B, Luu L, Lyakhovich A, Lyamzaev KG, Lystad AH, Lytvynchuk L, Ma AC, Ma C, Ma M, Ma NF, Ma QH, Ma X, Ma Y, Ma Z, MacDougald OA, Macian F, MacIntosh GC, MacKeigan JP, Macleod KF, Maday S, Madeo F, Madesh M, Madl T, Madrigal-Matute J, Maeda A, Maejima Y, Magarinos M, Mahavadi P, Maiani E, Maiese K, Maiti P, Maiuri MC, Majello B, Major MB, Makareeva E, Malik F, Mallilankaraman K, Malorni W, Maloyan A, Mammadova N, Man GCW, Manai F, Mancias JD, Mandelkow EM, Mandell MA, Manfredi AA, Manjili MH, Manjithaya R, Manque P, Manshian BB, Manzano R, Manzoni C, Mao K, Marchese C, Marchetti S, Marconi AM, Marcucci F, Mardente S, Mareninova OA, Margeta M, Mari M, Marinelli S, Marinelli O, Mariño G, Mariotto S, Marshall RS, Marten MR, Martens S, Martin APJ, Martin KR, Martin S, Martin S, Martín-Segura A, Martín-Acebes MA, Martin-Burriel I, Martin-Rincon M, Martin-Sanz P, Martina JA, Martinet W, Martinez A, Martinez A, Martinez J, Martinez Velazquez M, Martinez-Lopez N, Martinez-Vicente M, Martins DO, Martins JO, Martins WK, Martins-Marques T, Marzetti E, Masaldan S, Masclaux-Daubresse C, Mashek DG, Massa V, Massieu L, Masson GR, Masuelli L, Masyuk AI, Masyuk TV, Matarrese P, Matheu A, Matoba S, Matsuzaki S, Mattar P, Matte A, Mattoscio D, Mauriz JL, Mauthe M, Mauvezin C, Maverakis E, Maycotte P, Mayer J, Mazzoccoli G, Mazzoni C, Mazzulli JR, McCarty N, McDonald C, McGill MR, McKenna SL, McLaughlin B, McLoughlin F, McNiven MA, McWilliams TG, Mechta-Grigoriou F, Medeiros TC, Medina DL, Megeney LA, Megyeri K, Mehrpour M, Mehta JL, Meijer AJ, Meijer AH, Mejlvang J, Meléndez A, Melk A, Memisoglu G, Mendes AF, Meng D, Meng F, Meng T, Menna-Barreto R, Menon MB, Mercer C, Mercier AE, Mergny JL, Merighi A, Merkley SD, Merla G, Meske V, Mestre AC, Metur SP, Meyer C, Meyer H, Mi W, Mialet-Perez J, Miao J, Micale L, Miki Y, Milan E, Milczarek M, Miller DL, Miller SI, Miller S, Millward SW, Milosevic I, Minina EA, Mirzaei H, Mirzaei HR, Mirzaei M, Mishra A, Mishra N, Mishra PK, Misirkic Marjanovic M, Misasi R, Misra A, Misso G, Mitchell C, Mitou G, Miura T, Miyamoto S, Miyazaki M, Miyazaki M, Miyazaki T, Miyazawa K, Mizushima N, Mogensen TH, Mograbi B, Mohammadinejad R, Mohamud Y, Mohanty A, Mohapatra S, Möhlmann T, Mohmmed A, Moles A, Moley KH, Molinari M, Mollace V, Møller AB, Mollereau B, Mollinedo F, Montagna C, Monteiro MJ, Montella A, Montes LR, Montico B, Mony VK, Monzio Compagnoni G, Moore MN, Moosavi MA, Mora AL, Mora M, Morales-Alamo D, Moratalla R, Moreira PI, Morelli E, Moreno S, Moreno-Blas D, Moresi V, Morga B, Morgan AH, Morin F, Morishita H, Moritz OL, Moriyama M, Moriyasu Y, Morleo M, Morselli E, Moruno-Manchon JF, Moscat J, Mostowy S, Motori E, Moura AF, Moustaid-Moussa N, Mrakovcic M, Muciño-Hernández G, Mukherjee A, Mukhopadhyay S, Mulcahy Levy JM, Mulero V, Muller S, Münch C, Munjal A, Munoz-Canoves P, Muñoz-Galdeano T, Münz C, Murakawa T, Muratori C, Murphy BM, Murphy JP, Murthy A, Myöhänen TT, Mysorekar IU, Mytych J, Nabavi SM, Nabissi M, Nagy P, Nah J, Nahimana A, Nakagawa I, Nakamura K, Nakatogawa H, Nandi SS, Nanjundan M, Nanni M, Napolitano G, Nardacci R, Narita M, Nassif M, Nathan I, Natsumeda M, Naude RJ, Naumann C, Naveiras O, Navid F, Nawrocki ST, Nazarko TY, Nazio F, Negoita F, Neill T, Neisch AL, Neri LM, Netea MG, Neubert P, Neufeld TP, Neumann D, Neutzner A, Newton PT, Ney PA, Nezis IP, Ng CCW, Ng TB, Nguyen HTT, Nguyen LT, Ni HM, Ní Cheallaigh C, Ni Z, Nicolao MC, Nicoli F, Nieto-Diaz M, Nilsson P, Ning S, Niranjan R, Nishimune H, Niso-Santano M, Nixon RA, Nobili A, Nobrega C, Noda T, Nogueira-Recalde U, Nolan TM, Nombela I, Novak I, Novoa B, Nozawa T, Nukina N, Nussbaum-Krammer C, Nylandsted J, O'Donovan TR, O'Leary SM, O'Rourke EJ, O'Sullivan MP, O'Sullivan TE, Oddo S, Oehme I, Ogawa M, Ogier-Denis E, Ogmundsdottir MH, Ogretmen B, Oh GT, Oh SH, Oh YJ, Ohama T, Ohashi Y, Ohmuraya M, Oikonomou V, Ojha R, Okamoto K, Okazawa H, Oku M, Oliván S, Oliveira JMA, Ollmann M, Olzmann JA, Omari S, Omary MB, Önal G, Ondrej M, Ong SB, Ong SG, Onnis A, Orellana JA, Orellana-Muñoz S, Ortega-Villaizan MDM, Ortiz-Gonzalez XR, Ortona E, Osiewacz HD, Osman AK, Osta R, Otegui MS, Otsu K, Ott C, Ottobrini L, Ou JJ, Outeiro TF, Oynebraten I, Ozturk M, Pagès G, Pahari S, Pajares M, Pajvani UB, Pal R, Paladino S, Pallet N, Palmieri M, Palmisano G, Palumbo C, Pampaloni F, Pan L, Pan Q, Pan W, Pan X, Panasyuk G, Pandey R, Pandey UB, Pandya V, Paneni F, Pang SY, Panzarini E, Papademetrio DL, Papaleo E, Papinski D, Papp D, Park EC, Park HT, Park JM, Park JI, Park JT, Park J, Park SC, Park SY, Parola AH, Parys JB, Pasquier A, Pasquier B, Passos JF, Pastore N, Patel HH, Patschan D, Pattingre S, Pedraza-Alva G, Pedraza-Chaverri J, Pedrozo Z, Pei G, Pei J, Peled-Zehavi H, Pellegrini JM, Pelletier J, Peñalva MA, Peng D, Peng Y, Penna F, Pennuto M, Pentimalli F, Pereira CM, Pereira GJS, Pereira LC, Pereira de Almeida L, Perera ND, Pérez-Lara Á, Perez-Oliva AB, Pérez-Pérez ME, Periyasamy P, Perl A, Perrotta C, Perrotta I, Pestell RG, Petersen M, Petrache I, Petrovski G, Pfirrmann T, Pfister AS, Philips JA, Pi H, Picca A, Pickrell AM, Picot S, Pierantoni GM, Pierdominici M, Pierre P, Pierrefite-Carle V, Pierzynowska K, Pietrocola F, Pietruczuk M, Pignata C, Pimentel-Muiños FX, Pinar M, Pinheiro RO, Pinkas-Kramarski R, Pinton P, Pircs K, Piya S, Pizzo P, Plantinga TS, Platta HW, Plaza-Zabala A, Plomann M, Plotnikov EY, Plun-Favreau H, Pluta R, Pocock R, Pöggeler S, Pohl C, Poirot M, Poletti A, Ponpuak M, Popelka H, Popova B, Porta H, Porte Alcon S, Portilla-Fernandez E, Post M, Potts MB, Poulton J, Powers T, Prahlad V, Prajsnar TK, Praticò D, Prencipe R, Priault M, Proikas-Cezanne T, Promponas VJ, Proud CG, Puertollano R, Puglielli L, Pulinilkunnil T, Puri D, Puri R, Puyal J, Qi X, Qi Y, Qian W, Qiang L, Qiu Y, Quadrilatero J, Quarleri J, Raben N, Rabinowich H, Ragona D, Ragusa MJ, Rahimi N, Rahmati M, Raia V, Raimundo N, Rajasekaran NS, Ramachandra Rao S, Rami A, Ramírez-Pardo I, Ramsden DB, Randow F, Rangarajan PN, Ranieri D, Rao H, Rao L, Rao R, Rathore S, Ratnayaka JA, Ratovitski EA, Ravanan P, Ravegnini G, Ray SK, Razani B, Rebecca V, Reggiori F, Régnier-Vigouroux A, Reichert AS, Reigada D, Reiling JH, Rein T, Reipert S, Rekha RS, Ren H, Ren J, Ren W, Renault T, Renga G, Reue K, Rewitz K, Ribeiro de Andrade Ramos B, Riazuddin SA, Ribeiro-Rodrigues TM, Ricci JE, Ricci R, Riccio V, Richardson DR, Rikihisa Y, Risbud MV, Risueño RM, Ritis K, Rizza S, Rizzuto R, Roberts HC, Roberts LD, Robinson KJ, Roccheri MC, Rocchi S, Rodney GG, Rodrigues T, Rodrigues Silva VR, Rodriguez A, Rodriguez-Barrueco R, Rodriguez-Henche N, Rodriguez-Rocha H, Roelofs J, Rogers RS, Rogov VV, Rojo AI, Rolka K, Romanello V, Romani L, Romano A, Romano PS, Romeo-Guitart D, Romero LC, Romero M, Roney JC, Rongo C, Roperto S, Rosenfeldt MT, Rosenstiel P, Rosenwald AG, Roth KA, Roth L, Roth S, Rouschop KMA, Roussel BD, Roux S, Rovere-Querini P, Roy A, Rozieres A, Ruano D, Rubinsztein DC, Rubtsova MP, Ruckdeschel K, Ruckenstuhl C, Rudolf E, Rudolf R, Ruggieri A, Ruparelia AA, Rusmini P, Russell RR, Russo GL, Russo M, Russo R, Ryabaya OO, Ryan KM, Ryu KY, Sabater-Arcis M, Sachdev U, Sacher M, Sachse C, Sadhu A, Sadoshima J, Safren N, Saftig P, Sagona AP, Sahay G, Sahebkar A, Sahin M, Sahin O, Sahni S, Saito N, Saito S, Saito T, Sakai R, Sakai Y, Sakamaki JI, Saksela K, Salazar G, Salazar-Degracia A, Salekdeh GH, Saluja AK, Sampaio-Marques B, Sanchez MC, Sanchez-Alcazar JA, Sanchez-Vera V, Sancho-Shimizu V, Sanderson JT, Sandri M, Santaguida S, Santambrogio L, Santana MM, Santoni G, Sanz A, Sanz P, Saran S, Sardiello M, Sargeant TJ, Sarin A, Sarkar C, Sarkar S, Sarrias MR, Sarkar S, Sarmah DT, Sarparanta J, Sathyanarayan A, Sathyanarayanan R, Scaglione KM, Scatozza F, Schaefer L, Schafer ZT, Schaible UE, Schapira AHV, Scharl M, Schatzl HM, Schein CH, Scheper W, Scheuring D, Schiaffino MV, Schiappacassi M, Schindl R, Schlattner U, Schmidt O, Schmitt R, Schmidt SD, Schmitz I, Schmukler E, Schneider A, Schneider BE, Schober R, Schoijet AC, Schott MB, Schramm M, Schröder B, Schuh K, Schüller C, Schulze RJ, Schürmanns L, Schwamborn JC, Schwarten M, Scialo F, Sciarretta S, Scott MJ, Scotto KW, Scovassi AI, Scrima A, Scrivo A, Sebastian D, Sebti S, Sedej S, Segatori L, Segev N, Seglen PO, Seiliez I, Seki E, Selleck SB, Sellke FW, Selsby JT, Sendtner M, Senturk S, Seranova E, Sergi C, Serra-Moreno R, Sesaki H, Settembre C, Setty SRG, Sgarbi G, Sha O, Shacka JJ, Shah JA, Shang D, Shao C, Shao F, Sharbati S, Sharkey LM, Sharma D, Sharma G, Sharma K, Sharma P, Sharma S, Shen HM, Shen H, Shen J, Shen M, Shen W, Shen Z, Sheng R, Sheng Z, Sheng ZH, Shi J, Shi X, Shi YH, Shiba-Fukushima K, Shieh JJ, Shimada Y, Shimizu S, Shimozawa M, Shintani T, Shoemaker CJ, Shojaei S, Shoji I, Shravage BV, Shridhar V, Shu CW, Shu HB, Shui K, Shukla AK, Shutt TE, Sica V, Siddiqui A, Sierra A, Sierra-Torre V, Signorelli S, Sil P, Silva BJA, Silva JD, Silva-Pavez E, Silvente-Poirot S, Simmonds RE, Simon AK, Simon HU, Simons M, Singh A, Singh LP, Singh R, Singh SV, Singh SK, Singh SB, Singh S, Singh SP, Sinha D, Sinha RA, Sinha S, Sirko A, Sirohi K, Sivridis EL, Skendros P, Skirycz A, Slaninová I, Smaili SS, Smertenko A, Smith MD, Soenen SJ, Sohn EJ, Sok SPM, Solaini G, Soldati T, Soleimanpour SA, Soler RM, Solovchenko A, Somarelli JA, Sonawane A, Song F, Song HK, Song JX, Song K, Song Z, Soria LR, Sorice M, Soukas AA, Soukup SF, Sousa D, Sousa N, Spagnuolo PA, Spector SA, Srinivas Bharath MM, St Clair D, Stagni V, Staiano L, Stalnecker CA, Stankov MV, Stathopulos PB, Stefan K, Stefan SM, Stefanis L, Steffan JS, Steinkasserer A, Stenmark H, Sterneckert J, Stevens C, Stoka V, Storch S, Stork B, Strappazzon F, Strohecker AM, Stupack DG, Su H, Su LY, Su L, Suarez-Fontes AM, Subauste CS, Subbian S, Subirada PV, Sudhandiran G, Sue CM, Sui X, Summers C, Sun G, Sun J, Sun K, Sun MX, Sun Q, Sun Y, Sun Z, Sunahara KKS, Sundberg E, Susztak K, Sutovsky P, Suzuki H, Sweeney G, Symons JD, Sze SCW, Szewczyk NJ, Tabęcka-Łonczynska A, Tabolacci C, Tacke F, Taegtmeyer H, Tafani M, Tagaya M, Tai H, Tait SWG, Takahashi Y, Takats S, Talwar P, Tam C, Tam SY, Tampellini D, Tamura A, Tan CT, Tan EK, Tan YQ, Tanaka M, Tanaka M, Tang D, Tang J, Tang TS, Tanida I, Tao Z, Taouis M, Tatenhorst L, Tavernarakis N, Taylor A, Taylor GA, Taylor JM, Tchetina E, Tee AR, Tegeder I, Teis D, Teixeira N, Teixeira-Clerc F, Tekirdag KA, Tencomnao T, Tenreiro S, Tepikin AV, Testillano PS, Tettamanti G, Tharaux PL, Thedieck K, Thekkinghat AA, Thellung S, Thinwa JW, Thirumalaikumar VP, Thomas SM, Thomes PG, Thorburn A, Thukral L, Thum T, Thumm M, Tian L, Tichy A, Till A, Timmerman V, Titorenko VI, Todi SV, Todorova K, Toivonen JM, Tomaipitinca L, Tomar D, Tomas-Zapico C, Tomić S, Tong BC, Tong C, Tong X, Tooze SA, Torgersen ML, Torii S, Torres-López L, Torriglia A, Towers CG, Towns R, Toyokuni S, Trajkovic V, Tramontano D, Tran QG, Travassos LH, Trelford CB, Tremel S, Trougakos IP, Tsao BP, Tschan MP, Tse HF, Tse TF, Tsugawa H, Tsvetkov AS, Tumbarello DA, Tumtas Y, Tuñón MJ, Turcotte S, Turk B, Turk V, Turner BJ, Tuxworth RI, Tyler JK, Tyutereva EV, Uchiyama Y, Ugun-Klusek A, Uhlig HH, Ułamek-Kozioł M, Ulasov IV, Umekawa M, Ungermann C, Unno R, Urbe S, Uribe-Carretero E, Üstün S, Uversky VN, Vaccari T, Vaccaro MI, Vahsen BF, Vakifahmetoglu-Norberg H, Valdor R, Valente MJ, Valko A, Vallee RB, Valverde AM, Van den Berghe G, van der Veen S, Van Kaer L, van Loosdregt J, van Wijk SJL, Vandenberghe W, Vanhorebeek I, Vannier-Santos MA, Vannini N, Vanrell MC, Vantaggiato C, Varano G, Varela-Nieto I, Varga M, Vasconcelos MH, Vats S, Vavvas DG, Vega-Naredo I, Vega-Rubin-de-Celis S, Velasco G, Velázquez AP, Vellai T, Vellenga E, Velotti F, Verdier M, Verginis P, Vergne I, Verkade P, Verma M, Verstreken P, Vervliet T, Vervoorts J, Vessoni AT, Victor VM, Vidal M, Vidoni C, Vieira OV, Vierstra RD, Viganó S, Vihinen H, Vijayan V, Vila M, Vilar M, Villalba JM, Villalobo A, Villarejo-Zori B, Villarroya F, Villarroya J, Vincent O, Vindis C, Viret C, Viscomi MT, Visnjic D, Vitale I, Vocadlo DJ, Voitsekhovskaja OV, Volonté C, Volta M, Vomero M, Von Haefen C, Vooijs MA, Voos W, Vucicevic L, Wade-Martins R, Waguri S, Waite KA, Wakatsuki S, Walker DW, Walker MJ, Walker SA, Walter J, Wandosell FG, Wang B, Wang CY, Wang C, Wang C, Wang C, Wang CY, Wang D, Wang F, Wang F, Wang F, Wang G, Wang H, Wang H, Wang H, Wang HG, Wang J, Wang J, Wang J, Wang J, Wang K, Wang L, Wang L, Wang MH, Wang M, Wang N, Wang P, Wang P, Wang P, Wang P, Wang QJ, Wang Q, Wang QK, Wang QA, Wang WT, Wang W, Wang X, Wang X, Wang Y, Wang Y, Wang Y, Wang YY, Wang Y, Wang Y, Wang Y, Wang Y, Wang Z, Wang Z, Wang Z, Warnes G, Warnsmann V, Watada H, Watanabe E, Watchon M, Wawrzyńska A, Weaver TE, Wegrzyn G, Wehman AM, Wei H, Wei L, Wei T, Wei Y, Weiergräber OH, Weihl CC, Weindl G, Weiskirchen R, Wells A, Wen RH, Wen X, Werner A, Weykopf B, Wheatley SP, Whitton JL, Whitworth AJ, Wiktorska K, Wildenberg ME, Wileman T, Wilkinson S, Willbold D, Williams B, Williams RSB, Williams RL, Williamson PR, Wilson RA, Winner B, Winsor NJ, Witkin SS, Wodrich H, Woehlbier U, Wollert T, Wong E, Wong JH, Wong RW, Wong VKW, Wong WW, Wu AG, Wu C, Wu J, Wu J, Wu KK, Wu M, Wu SY, Wu S, Wu SY, Wu S, Wu WKK, Wu X, Wu X, Wu YW, Wu Y, Xavier RJ, Xia H, Xia L, Xia Z, Xiang G, Xiang J, Xiang M, Xiang W, Xiao B, Xiao G, Xiao H, Xiao HT, Xiao J, Xiao L, Xiao S, Xiao Y, Xie B, Xie CM, Xie M, Xie Y, Xie Z, Xie Z, Xilouri M, Xu C, Xu E, Xu H, Xu J, Xu J, Xu L, Xu WW, Xu X, Xue Y, Yakhine-Diop SMS, Yamaguchi M, Yamaguchi O, Yamamoto A, Yamashina S, Yan S, Yan SJ, Yan Z, Yanagi Y, Yang C, Yang DS, Yang H, Yang HT, Yang H, Yang JM, Yang J, Yang J, Yang L, Yang L, Yang M, Yang PM, Yang Q, Yang S, Yang S, Yang SF, Yang W, Yang WY, Yang X, Yang X, Yang Y, Yang Y, Yao H, Yao S, Yao X, Yao YG, Yao YM, Yasui T, Yazdankhah M, Yen PM, Yi C, Yin XM, Yin Y, Yin Z, Yin Z, Ying M, Ying Z, Yip CK, Yiu SPT, Yoo YH, Yoshida K, Yoshii SR, Yoshimori T, Yousefi B, Yu B, Yu H, Yu J, Yu J, Yu L, Yu ML, Yu SW, Yu VC, Yu WH, Yu Z, Yu Z, Yuan J, Yuan LQ, Yuan S, Yuan SF, Yuan Y, Yuan Z, Yue J, Yue Z, Yun J, Yung RL, Zacks DN, Zaffagnini G, Zambelli VO, Zanella I, Zang QS, Zanivan S, Zappavigna S, Zaragoza P, Zarbalis KS, Zarebkohan A, Zarrouk A, Zeitlin SO, Zeng J, Zeng JD, Žerovnik E, Zhan L, Zhang B, Zhang DD, Zhang H, Zhang H, Zhang H, Zhang H, Zhang H, Zhang H, Zhang H, Zhang HL, Zhang J, Zhang J, Zhang JP, Zhang KYB, Zhang LW, Zhang L, Zhang L, Zhang L, Zhang L, Zhang M, Zhang P, Zhang S, Zhang W, Zhang X, Zhang XW, Zhang X, Zhang X, Zhang X, Zhang X, Zhang XD, Zhang Y, Zhang Y, Zhang Y, Zhang YD, Zhang Y, Zhang YY, Zhang Y, Zhang Z, Zhang Z, Zhang Z, Zhang Z, Zhang Z, Zhang Z, Zhao H, Zhao L, Zhao S, Zhao T, Zhao XF, Zhao Y, Zhao Y, Zhao Y, Zhao Y, Zheng G, Zheng K, Zheng L, Zheng S, Zheng XL, Zheng Y, Zheng ZG, Zhivotovsky B, Zhong Q, Zhou A, Zhou B, Zhou C, Zhou G, Zhou H, Zhou H, Zhou H, Zhou J, Zhou J, Zhou J, Zhou J, Zhou K, Zhou R, Zhou XJ, Zhou Y, Zhou Y, Zhou Y, Zhou ZY, Zhou Z, Zhu B, Zhu C, Zhu GQ, Zhu H, Zhu H, Zhu H, Zhu WG, Zhu Y, Zhu Y, Zhuang H, Zhuang X, Zientara-Rytter K, Zimmermann CM, Ziviani E, Zoladek T, Zong WX, Zorov DB, Zorzano A, Zou W, Zou Z, Zou Z, Zuryn S, Zwerschke W, Brand-Saberi B, Dong XC, Kenchappa CS, Li Z, Lin Y, Oshima S, Rong Y, Sluimer JC, Stallings CL, Tong CK. Guidelines for the use and interpretation of assays for monitoring autophagy (4th edition). Autophagy 2021;17:1–382. 33634751 10.1080/15548627.2020.1797280 PMC7996087

[42] Schiffrin EL . Does endothelin-1 raise or lower blood pressure in humans? Nephron 2018;139:47–50. 10.1159/000487346 29448245

[43] Stankova J, Rola-Pleszczynski M, D'Orleans-Juste P. Endothelin 1 and thrombin synergistically stimulate IL-6 mRNA expression and protein production in human umbilical vein endothelial cells. J Cardiovasc Pharmacol 1995;26:S505–S507. 8587460

[44] Zhou HY, Sui H, Zhao YJ, Qian HJ, Yang N, Liu L, Guan Q, Zhou Y, Lin HL, Wang DP. The impact of inflammatory immune reactions of the vascular niche on organ fibrosis. Front Pharmacol 2021;12:750509. 34776968 10.3389/fphar.2021.750509 PMC8585779

[45] Kopp HP, Kopp CW, Festa A, Krzyzanowska K, Kriwanek S, Minar E, Roka R, Schernthaner G. Impact of weight loss on inflammatory proteins and their association with the insulin resistance syndrome in morbidly obese patients. Arterioscler Thromb Vasc Biol 2003;23:1042–1047. 12714437 10.1161/01.ATV.0000073313.16135.21

[46] Mossmann M, Wainstein MV, Mariani S, Machado GP, de Araujo GN, Andrades M, Gonçalves SC, Bertoluci MC. Increased serum IL-6 is predictive of long-term cardiovascular events in high-risk patients submitted to coronary angiography: an observational study. Diabetol Metab Syndr 2022;14:125. 36028849 10.1186/s13098-022-00891-0 PMC9419425

[47] Rose-John S, Jenkins BJ, Garbers C, Moll JM, Scheller J. Targeting IL-6 trans-signalling: past, present and future prospects. Nat Rev Immunol 2023;23:666–681. 37069261 10.1038/s41577-023-00856-y PMC10108826

[48] Tang WH, Kitai T, Hazen SL. Gut Microbiota in cardiovascular health and disease. Circ Res 2017;120:1183–1196. 28360349 10.1161/CIRCRESAHA.117.309715 PMC5390330

[49] Gubatan J, Boye TL, Temby M, Sojwal RS, Holman DR, Sinha SR, Rogalla SR, Nielsen OH. Gut microbiome in inflammatory bowel disease: role in pathogenesis, dietary modulation, and colitis-associated colon cancer. Microorganisms 2022;10:1371. 35889090 10.3390/microorganisms10071371 PMC9316834

[50] Ridker PM, Everett BM, Thuren T, MacFadyen JG, Chang WH, Ballantyne C, Fonseca F, Nicolau J, Koenig W, Anker SD, Kastelein JJP, Cornel JH, Pais P, Pella D, Genest J, Cifkova R, Lorenzatti A, Forster T, Kobalava Z, Vida-Simiti L, Flather M, Shimokawa H, Ogawa H, Dellborg M, Rossi PRF, Troquay RPT, Libby P, Glynn RJ; CANTOS Trial Group. Antiinflammatory therapy with canakinumab for atherosclerotic disease. N Engl J Med 2017;377:1119–1131. 28845751 10.1056/NEJMoa1707914

[51] Ridker PM, MacFadyen JG, Thuren T, Libby P. Residual inflammatory risk associated with interleukin-18 and interleukin-6 after successful interleukin-1beta inhibition with canakinumab: further rationale for the development of targeted anti-cytokine therapies for the treatment of atherothrombosis. Eur Heart J 2020;41:2153–2163. 31504417 10.1093/eurheartj/ehz542

[52] IL6R Genetics Consortium Emerging Risk Factors Collaboration; Sarwar N, Butterworth AS, Freitag DF, Gregson J, Willeit P, Gorman DN, Gao P, Saleheen D, Rendon A, Nelson CP, Braund PS, Hall AS, Chasman DI, Tybjærg-Hansen A, Chambers JC, Benjamin EJ, Franks PW, Clarke R, Wilde AA, Trip MD, Steri M, Witteman JC, Qi L, van der Schoot CE, de Faire U, Erdmann J, Stringham HM, Koenig W, Rader DJ, Melzer D, Reich D, Psaty BM, Kleber ME, Panagiotakos DB, Willeit J, Wennberg P, Woodward M, Adamovic S, Rimm EB, Meade TW, Gillum RF, Shaffer JA, Hofman A, Onat A, Sundström J, Wassertheil-Smoller S, Mellström D, Gallacher J, Cushman M, Tracy RP, Kauhanen J, Karlsson M, Salonen JT, Wilhelmsen L, Amouyel P, Cantin B, Best LG, Ben-Shlomo Y, Manson JE, Davey-Smith G, de Bakker PI, O'Donnell CJ, Wilson JF, Wilson AG, Assimes TL, Jansson JO, Ohlsson C, Tivesten Å, Ljunggren Ö, Reilly MP, Hamsten A, Ingelsson E, Cambien F, Hung J, Thomas GN, Boehnke M, Schunkert H, Asselbergs FW, Kastelein JJ, Gudnason V, Salomaa V, Harris TB, Kooner JS, Allin KH, Nordestgaard BG, Hopewell JC, Goodall AH, Ridker PM, Hólm H, Watkins H, Ouwehand WH, Samani NJ, Kaptoge S, Di Angelantonio E, Harari O, Danesh J. Interleukin-6 receptor pathways in coronary heart disease: a collaborative meta-analysis of 82 studies. Lancet 2012;379:1205–1213. 22421339 10.1016/S0140-6736(11)61931-4 PMC3316940

[53] Schieffer B, Schieffer E, Hilfiker-Kleiner D, Hilfiker A, Kovanen PT, Kaartinen M, Nussberger J, Harringer W, Drexler H. Expression of angiotensin II and interleukin 6 in human coronary atherosclerotic plaques: potential implications for inflammation and plaque instability. Circulation 2000;101:1372–1378. 10736279 10.1161/01.cir.101.12.1372

[54] Huber SA, Sakkinen P, Conze D, Hardin N, Tracy R. Interleukin-6 exacerbates early atherosclerosis in mice. Arterioscler Thromb Vasc Biol 1999;19:2364–2367. 10521365 10.1161/01.atv.19.10.2364

[55] Fernandez-Ruiz I . Promising anti-IL-6 therapy for atherosclerosis. Nat Rev Cardiol 2021;18:544. 10.1038/s41569-021-00575-8 34040184

[56] Ridker PM, Devalaraja M, Baeres FMM, Engelmann MDM, Hovingh GK, Ivkovic M, Lo L, Kling D, Pergola P, Raj D, Libby P, Davidson M. IL-6 inhibition with ziltivekimab in patients at high atherosclerotic risk (RESCUE): a double-blind, randomised, placebo-controlled, phase 2 trial. Lancet 2021;397:2060–2069. 34015342 10.1016/S0140-6736(21)00520-1

[57] Madan M, Bishayi B, Hoge M, Amar S. Atheroprotective role of interleukin-6 in diet- and/or pathogen-associated atherosclerosis using an ApoE heterozygote murine model. Atherosclerosis 2008;197:504–514. 17412346 10.1016/j.atherosclerosis.2007.02.023 PMC2430020

[58] Schieffer B, Selle T, Hilfiker A, Hilfiker-Kleiner D, Grote K, Tietge UJ, Trautwein C, Luchtefeld M, Schmittkamp C, Heeneman Sa, Daemen MJAP, Drexler H. Impact of interleukin-6 on plaque development and morphology in experimental atherosclerosis. Circulation 2004;110:3493–3500. 15557373 10.1161/01.CIR.0000148135.08582.97

[59] Wassmann S, Stumpf M, Strehlow K, Schmid A, Schieffer B, Bohm M, Nickenig G. Interleukin-6 induces oxidative stress and endothelial dysfunction by overexpression of the angiotensin II type 1 receptor. Circ Res 2004;94:534–541. 14699015 10.1161/01.RES.0000115557.25127.8D

[60] Fielding CA, McLoughlin RM, McLeod L, Colmont CS, Najdovska M, Grail D, Ernst M, Jones SA, Topley N, Jenkins BJ. IL-6 regulates neutrophil trafficking during acute inflammation via STAT3. J Immunol 2008;181:2189–2195. 18641358 10.4049/jimmunol.181.3.2189

[61] Wenzel P, Knorr M, Kossmann S, Stratmann J, Hausding M, Schuhmacher S, Karbach SH, Schwenk M, Yogev N, Schulz E, Oelze M, Grabbe S, Jonuleit H, Becker C, Daiber A, Waisman A, Münzel T. Lysozyme M-positive monocytes mediate angiotensin II-induced arterial hypertension and vascular dysfunction. Circulation 2011;124:1370–1381. 21875910 10.1161/CIRCULATIONAHA.111.034470

[62] Didion SP . Cellular and oxidative mechanisms associated with interleukin-6 signaling in the vasculature. Int J Mol Sci 2017;18:2563. 29186034 10.3390/ijms18122563 PMC5751166

[63] Yabluchanskiy A, Ma Y, Iyer RP, Hall ME, Lindsey ML. Matrix metalloproteinase-9: many shades of function in cardiovascular disease. Physiology (Bethesda) 2013;28:391–403. 24186934 10.1152/physiol.00029.2013 PMC3858212

[64] Gomez D, Owens GK. Smooth muscle cell phenotypic switching in atherosclerosis. Cardiovasc Res 2012;95:156–164. 22406749 10.1093/cvr/cvs115 PMC3388816

[65] Baumer Y, McCurdy S, Alcala M, Mehta N, Lee BH, Ginsberg MH, Boisvert WA. CD98 regulates vascular smooth muscle cell proliferation in atherosclerosis. Atherosclerosis 2017;256:105–114. 28012647 10.1016/j.atherosclerosis.2016.11.017 PMC5276722

[66] Rautureau Y, Coelho SC, Fraulob-Aquino JC, Huo KG, Rehman A, Offermanns S, Paradis P, Schiffrin EL. Inducible human endothelin-1 overexpression in endothelium raises blood pressure via endothelin type A receptors. Hypertension 2015;66:347–355. 26101346 10.1161/HYPERTENSIONAHA.115.05168

[67] Hocher B, Thone-Reineke C, Rohmeiss P, Schmager F, Slowinski T, Burst V, van der Woude F, Bauer C, Theuring F. Endothelin-1 transgenic mice develop glomerulosclerosis, interstitial fibrosis, and renal cysts but not hypertension. J Clin Invest 1997;99:1380–1389. 9077548 10.1172/JCI119297 PMC507954

[68] Montgomery A, Tam F, Gursche C, Cheneval C, Besler K, Enns W, Manku S, Rey K, Hanson PJ, Rose-John S, McManus BM, Choy JC. Overlapping and distinct biological effects of IL-6 classic and trans-signaling in vascular endothelial cells. Am J Physiol Cell Physiol 2021;320:C554–C565. 33471622 10.1152/ajpcell.00323.2020

