# Supplementary material for: Myeloid cell-derived interleukin-6 induces vascular dysfunction and vascular and systemic inflammation
Source: Eur Heart J Open. 2024 Jun 12;4(4):oeae046. doi: 10.1093/ehjopen/oeae046 (PMC11250217; doi:10.1093/ehjopen/oeae046)
Supplement: oeae046_Supplementary_Data [file oeae046_supplementary_data.zip › 2024-04-28 Suppl. Material Karbach.docx]

**Myeloid cell derived Interleukin-6 induces vascular dysfunction and inflammation**

Tanja Knopp^1,2^*, Rebecca Jung^1,2^*, Johannes Wild^1,2,5^, Magdalena L. Bochenek^1,2,5^, Panagiotis Efentakis^11^, Annika Lehmann^1,2^, Tabea Bieler^1,2^, **Venkat Garlapati^2^,** Cindy Richter^9,10^, Michael Molitor^1,2,5^, Katharina Perius^2^, Stefanie Finger^2^, Jérémy Lagrange^2^, Iman Ghasemi^2^, Konstantinos Zifkos^2^, Katharina S. Kommoss^13^, Joumana Masri^3^, Sonja Reißig^3^, Voahanginirina Randriamboavonjy^7^, Thomas Wunderlich^6^, Nadine Hövelmeyer^3,4^, Alexander N.R. Weber^12^, Ilgiz A. Mufazalov^3^, Markus Bosmann^2,4,8^, Ingo Bechmann^9^, Ingrid Fleming^5,7^, Matthias Oelze^1^, Andreas Daiber^1^, Thomas Münzel^1,2,5^, Katrin Schäfer^1,5^, Philip Wenzel^1,2,5^, Ari Waisman^3,4*^, Susanne Karbach^1,2,5*^

**Supplementary Figure Legends**

Supplementary Figure 1: Inflammatory cells in the blood and spleen of mice overexpressing IL-6 in myeloid cells compared to control mice.

A. Gating strategy for myeloid cells in blood and spleen (here shown with spleen cells): We gated on the viable, B220 negative and CD90.2 negative cells and then on the CD11b positive and F4/80 positive myeloid cells to the further distinguish between Ly6G positive and Ly6C positive cells.

B. Quantification and representative flow cytometry plots of CD11b^+^ cells in the blood of LysM-IL-6^OE^ mice (red) compared to control mice (black). Pre-gated on viable cells which were B220 negative and CD90.2 negative. P = 0.0076.

C. Quantification and representative flow cytometry plots of Ly6G^+^Ly6C^+^ cells and Ly6G^-^Ly6C^+^ cells in the blood of LysM-IL-6^OE^ mice (red) compared to control mice (black) blood. Pre-gated on viable, B220^-^, CD90.2^-^, CD11b^+^ cells. P = 0.006 (Ly6G^+^Ly6C^+^).

D. Quantification and representative flow cytometry plots of CD90.2^+^ cells in the blood of LysM-IL-6^OE^ mice (red) compared to control mice (black). Pre-gated on viable, B220^-^ cells.

B-D: n = 9 – 15, unpaired Student`s t-test.

E. Quantification of the CD90.2^+^ cells in the spleen of LysM-IL-6^OE^ (red) compared to control mice (black) (analysis of total numbers (left) and percentage of living cells (right)). Pre-gated on viable, B220^-^ cells. n = 3 - 8, Mann-Whitney test. P = 0.012 (comparison of CD90.2^+^ percentage values).

Data are presented as mean ± SEM and *p* values of <0.05 were considered significant and marked by asterisks (**p* < 0.05; ***p* < 0.01; ****p* < 0.001).

**Supplementary Figure 2: Signs of systemic inflammation in LysM-IL-6^OE^ mice compared to control mice.**

H&E stainings of A. the lungs, B. the liver, C. the spleen, D. the kidneys and E. the brain of LysM-IL-6^OE^ mice and control mice, representative pictures are shown of n = 4 mice per group.

**Supplementary Figure 3: Endothelial dysfunction LysM-IL-6^OE^ mice is not accompanied by an increase in blood pressure.**

A. No difference in aortic relaxation in response to Glyceryl trinitrate (GTN) (smooth muscle cell dependent relaxation): Left: Aortic relaxation curves of LysM-IL-6^OE^ (red) versus control mice (black). Right: Maximal relaxation analyzed with unpaired Student´s t-test, n = 5 - 11.

B. Systolic blood pressure in LysM-IL-6^OE^ (red) versus control mice (black) measured by the tail-cuff method. Mann-Whitney and unpaired Student´s t-test; n = 10 - 12.

C. No difference in the mesenteric relaxation in response to sodium nitroprusside (SNP) (mesenteric arteries had been precontracted with Phe), unpaired Student´s t-test, n = 8.

Data are presented as mean ± SEM and *p* values of <0.05 were considered significant and marked by asterisks (**p* < 0.05; ***p* < 0.01; ****p* < 0.001).

**Supplementary Figure 4: No differences in (left and right ventricular) cardiac function of LysM-IL-6^OE^ mice in comparison to control mice.**

A. H&E stainings of the heart of LysM-IL-6^OE^ mice and control mice, representative pictures are shown of n = 4 mice per group.

B. Representative pictures of the M-mode image and B-mode image of the left ventricle obtained in parasternal long-axis (PLAX) of LysM-IL-6^OE^ and control mice obtained by high-frequency ultrasound echocardiography. n = 3. Lower row: Statistical analysis of the left ventricular anterior wall (LVAW) thickness, LV posterior wall (LVPW) thickness and the LV diameter (systolic and diastolic, measured in M-mode). Unpaired Student´s t-test.

C. Statistical analysis of measurement of left ventricular (LV) mass, stroke volume, cardiac output and LV with ejection fraction (LV-EF) measured by high-frequency ultrasound echocardiography obtained in parasternal long-axis (PLAX), n = 8, Student’s unpaired t-test or Mann-Whitney t-test.

D. Representative echocardiographic pictures in short-axis (SAX) and apical 4-chamber (4C) views with statistical analysis for the end-diastolic midventricular right ventricular internal diameter (RV-ID) and in SAX pulmonary valve velocity time integral (PV-VTI). n = 3, Mann-Whitney t-test.

Data are presented as mean ± SEM and *p* values of <0.05 were considered significant and marked by asterisks (**p* < 0.05; ***p* < 0.01; ****p* < 0.001).

**Supplementary Figure 5: Further characterization of vascular inflammation in LysM-IL-6^OE^ mice compared to control mice.**

A. Gating strategy for macrophages in the aortic vessel wall: We gated on the viable, CD45 positive and CD90.2 negative cells and then on the CD11b positive cells. Coming from the CD11b positive cells, we visualized the CX3CR1 positive and the CD115 positive macrophages.

B. Flow cytometric analysis of the CXCR1^+^ and the CD115^+^ CD11b^+^ macrophages in the aortic vessel wall of LysM-IL-6^OE^ mice compared to control mice. Pre-gating on living, CD45.2^+^ and B220^-^ cells and CD11b^+^ cells. n = 4 - 5 mice per group. Mann-Whitney test.

C. Flow cytometric analysis of the aortic CD90.2^+^ T cells in the aortic vessel wall of LysM-IL-6^OE^ mice compared to control mice. Pre-gating on living, CD45.2^+^ and B220^-^ cells, n = 3 - 7, Mann-Whitney test. P = 0.0333.

D. Flow cytometric analysis of the aortic CD4^+^ and CD8^+^ T cells in the aortic vessel wall of LysM-IL-6^OE^ mice compared to control mice. Pre-gating on living, CD45.2^+^ and CD90.2^+^ and B220^-^ cells, n = 3 - 7, Mann-Whitney test.

Data are presented as mean ± SEM and *p* values of <0.05 were considered significant and marked by asterisks (**p* < 0.05; ***p* < 0.01; ****p* < 0.001).

**Supplementary Figure 6: IL-6 is detectable in the supernatants of endothelial cells of LysM-IL-6^OE^ mice and the IL-6R alpha is present in the LysM-IL-6^OE^ aortas.**

A. After isolation of pulmonary endothelial cells from LysM-IL-6^OE^ mice and control mice, these cells were cultivated. IL-6 in the supernatants was measured after 24h by ELISA. Mann-Whitney test. P = 0.0003.

B. ELISA of (soluble) IL-6Ra in the aorta of LysM-IL-6^OE^ (red) and control mice (black). n = 3-8 mice per group. Concentration in the aorta was calculated per mg of aortic tissue. Mann-Whitney test. P = 0.0159.

Data are presented as mean ± SEM and *p* values of < 0.05 were considered significant and marked by asterisks (**p* < 0.05; ***p* < 0.01; ****p* < 0.001).

**Supplementary Figure 7: Short-term antioxidant treatment does not reduce vascular dysfunction in LysM-IL-6^OE^ aortas.**

ACh-induced relaxation was measured in the aortas of control and LysM-IL-6^OE^ mice with and without previous SOD (2000 U/ml) or Catalase (2000 U/ml) or ET-1 antagonist bosentan (10 µM) incubation over 1.5 hours at 37°C, respectively (n = 3-5 aortic rings of 3 - 4 mice per group). Two-way ANOVA Test with multiple comparison. Results of the statistical analysis of the endpoints are partially given in the figure. P (Control versus LysM-IL-6^OE^) = 0.0062; P (Control versus LysM-IL-6^OE^ plus SOD) = 0.0002; P (Control versus LysM-IL-6^OE^ plus Catalase) < 0.0001; P (Control versus LysM-IL-6^OE^ plus Bosentan) = 0.0129; P (LysM-IL-6^OE^ versus LysM-IL-6^OE^ plus Catalase) = 0.322.

Data are presented as mean ± SEM and *p* values of < 0.05 were considered significant and marked by asterisks (**p* < 0.05; ***p* < 0.01; ****p* < 0.001).

**Supplementary Figure 8: Analysis of fibrosis markers in the aortas of LysM-IL-6^OE^ mice compared to control mice.**

A.-F. Quantitative rt-PCR analysis for *Col1α1* (A), *Col2α1* (B), *Col3α1* (C), *Vimentin* (D), *MMP2* (E) and *MMP9* (F) in LysM-IL-6^OE^ mice aortas (red) versus control aortas. *Tbp* was used as housekeeping gene except for *Col3*α*1 and Vimentin* where *Gapdh* was used as housekeeping gene. N = 4 - 11 mice per group, either unpaired Student´s t-test or Mann-Whitney test. P = 0.04 (*MMP9*).

Data are presented as mean ± SEM and *p* values of < 0.05 were considered significant and marked by asterisks (**p* < 0.05; ***p* < 0.01; ****p* < 0.001).
