# Supplementary figures and images for: Myeloid cell-derived interleukin-6 induces vascular dysfunction and vascular and systemic inflammation
Source: Eur Heart J Open. 2024 Jun 12;4(4):oeae046. doi: 10.1093/ehjopen/oeae046 (PMC11250217; doi:10.1093/ehjopen/oeae046)

# Supplementary Figure 1

A

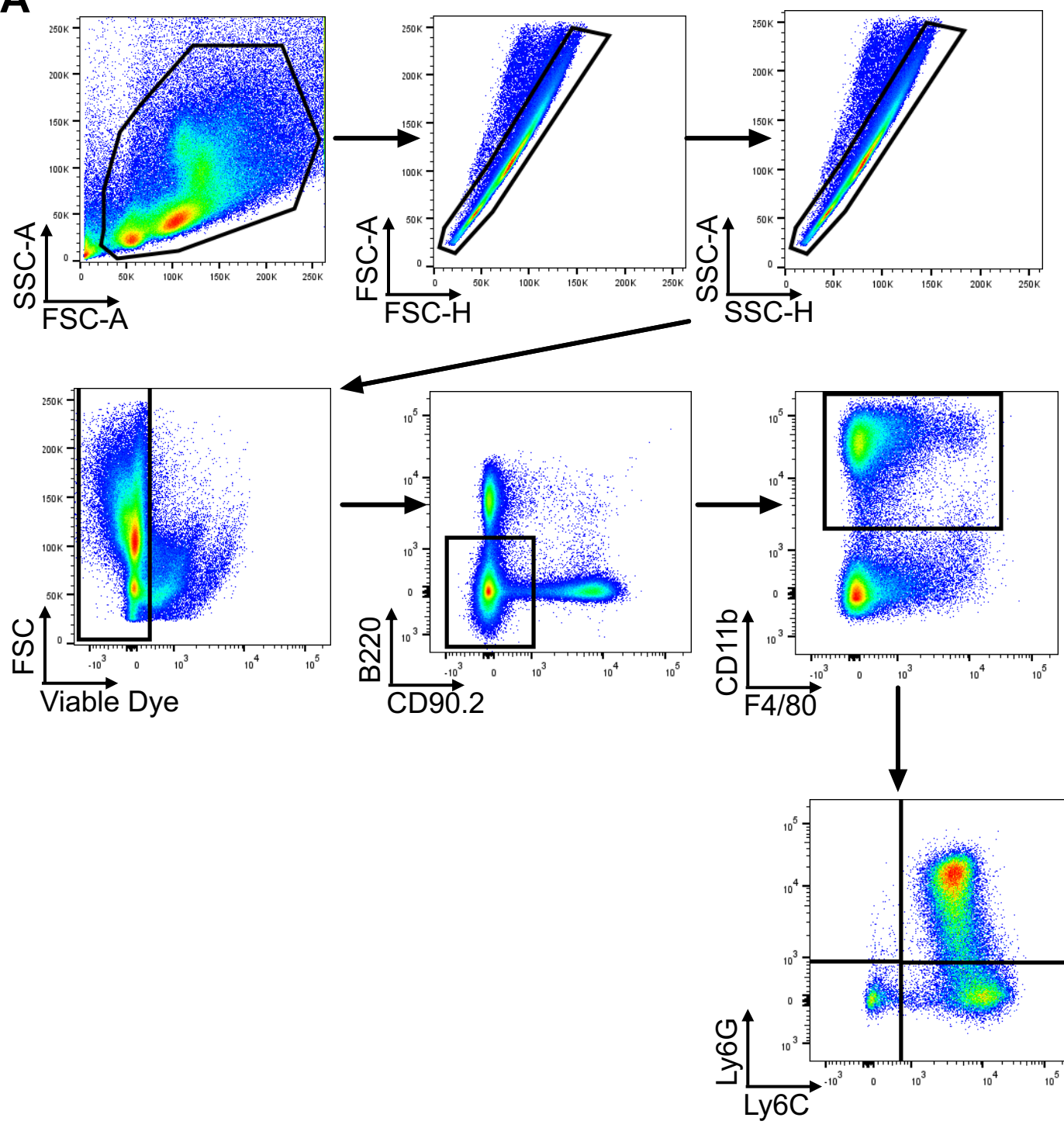

## B Blood

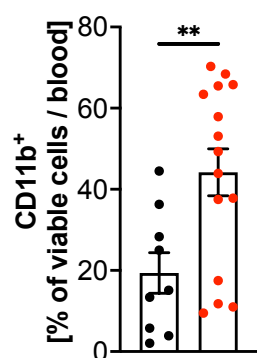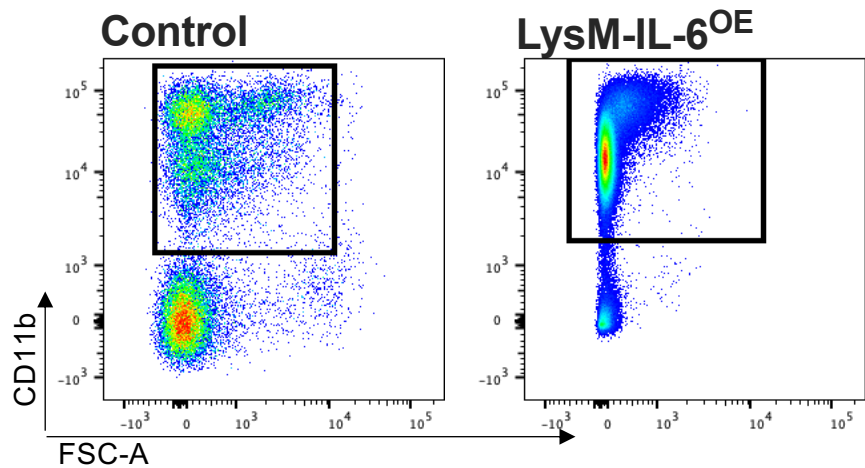

## C

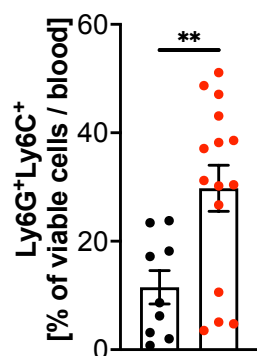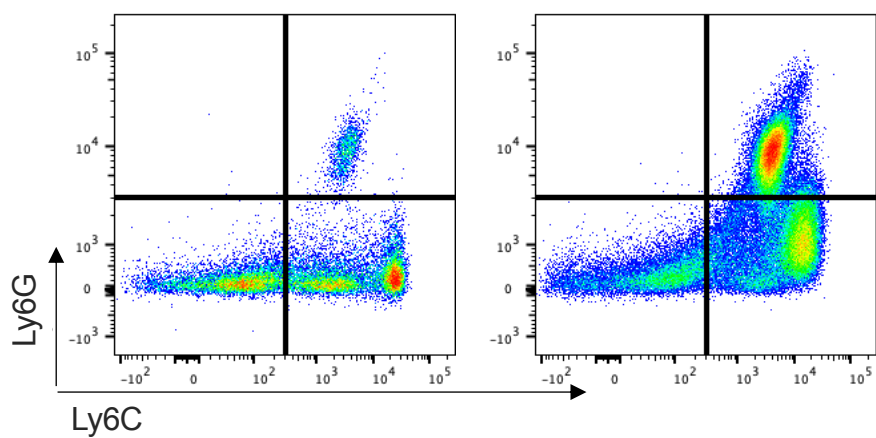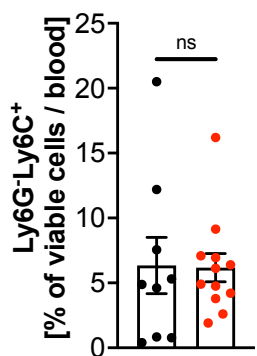

## D

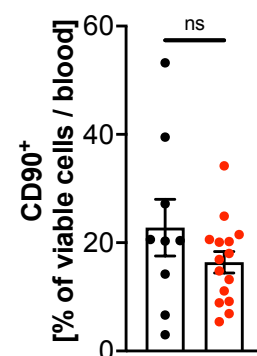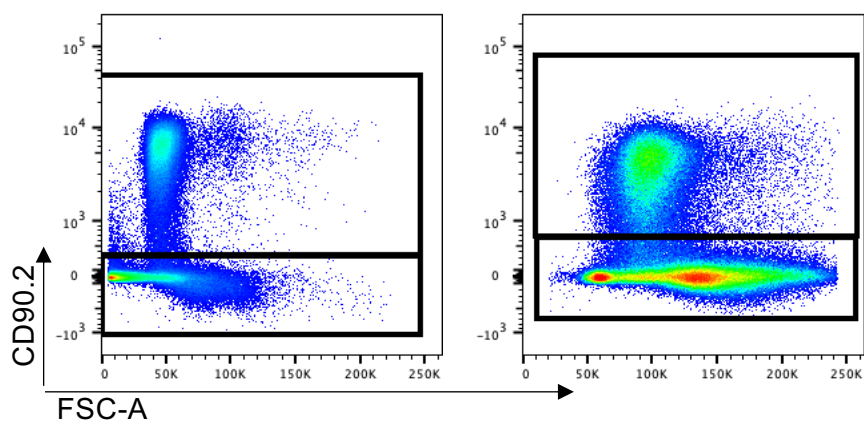

## E Spleen

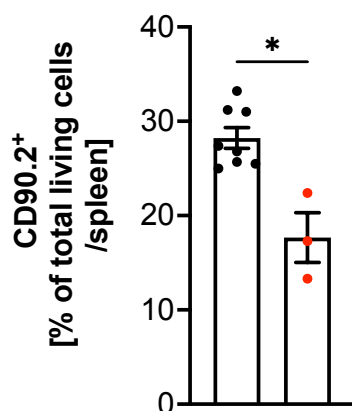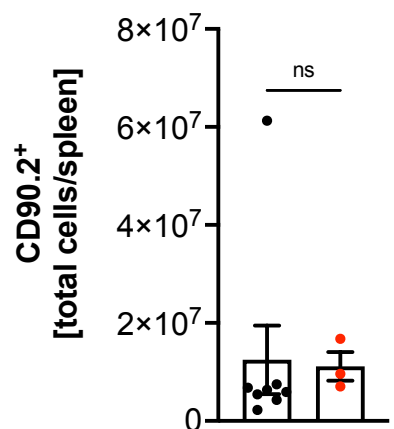

Supplement: oeae046_Supplementary_Data [file oeae046_supplementary_data.zip › 2024-04-28 Suppl.Fig.1 kombiniert.pdf]

# Supplementary Figure 2

## A Lungs

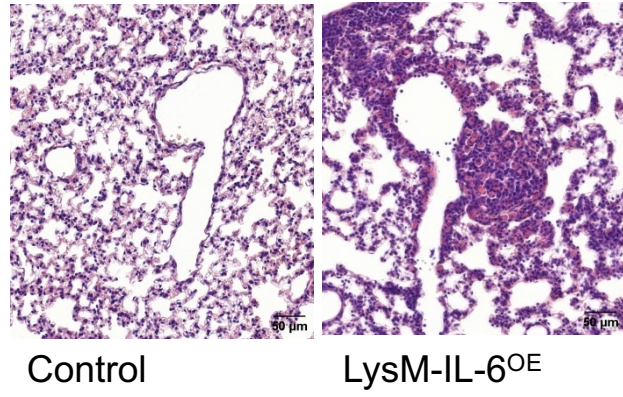

## C Spleen

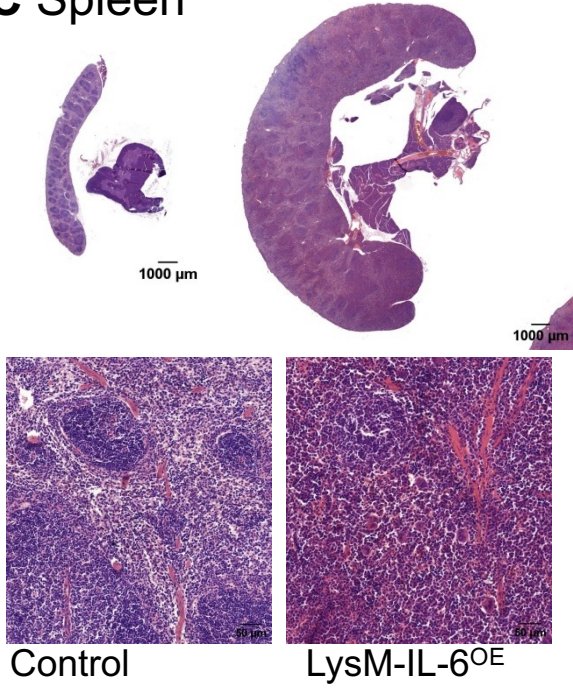

## B Liver

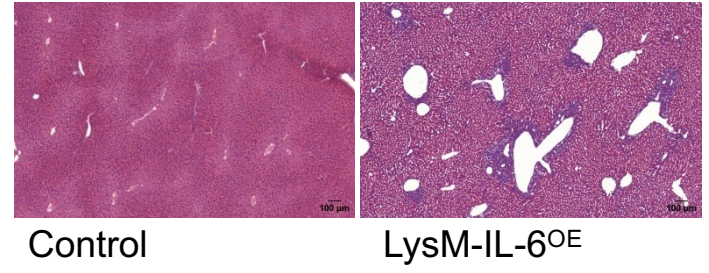

## D Kidneys

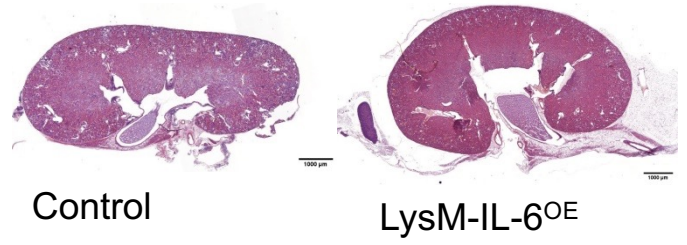

## E Brain

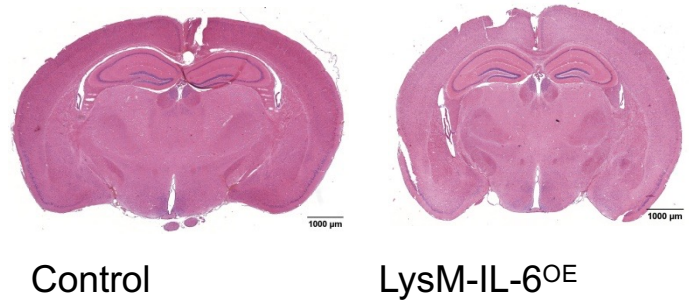

Supplement: oeae046_Supplementary_Data [file oeae046_supplementary_data.zip › 2024-04-28 Suppl.Fig.2.pdf]

**Supplementary Figure 3**

**A**

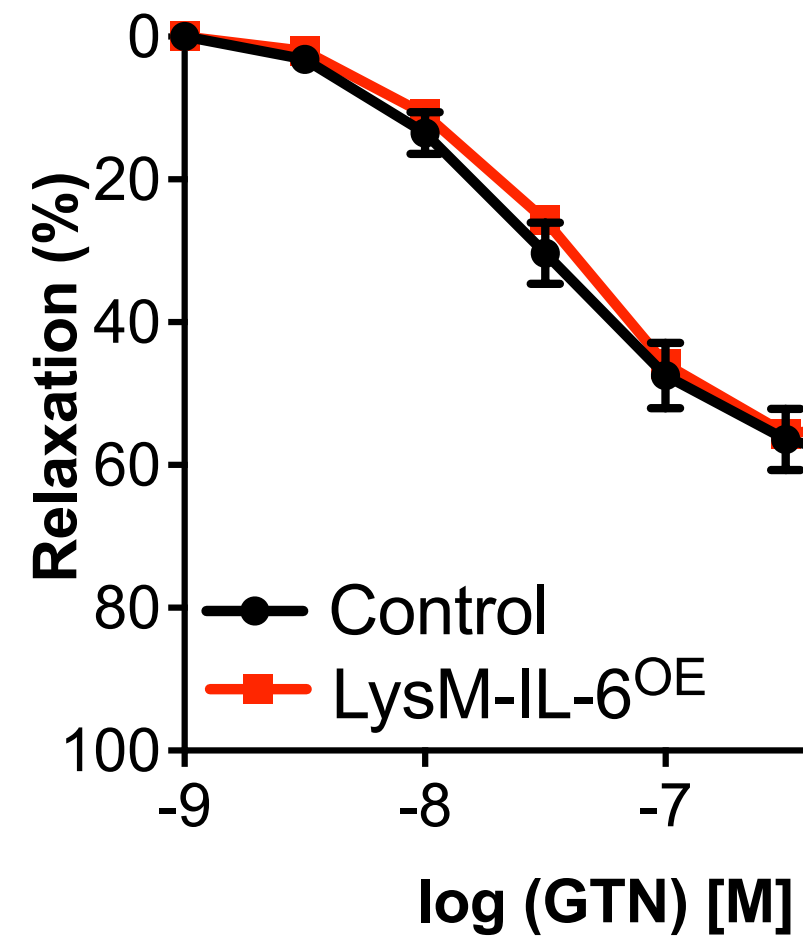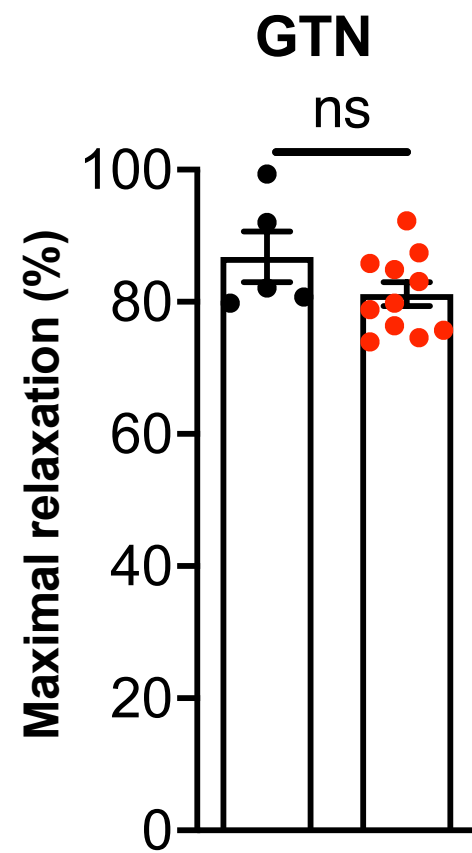

**B**

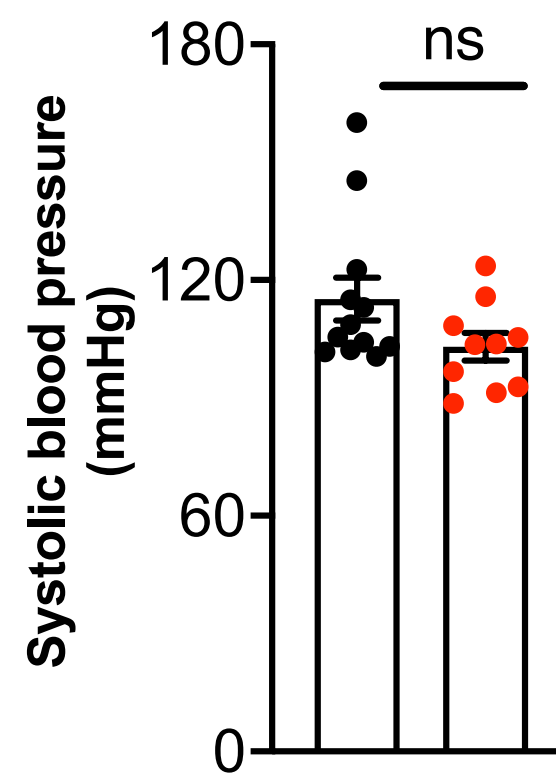

**C**

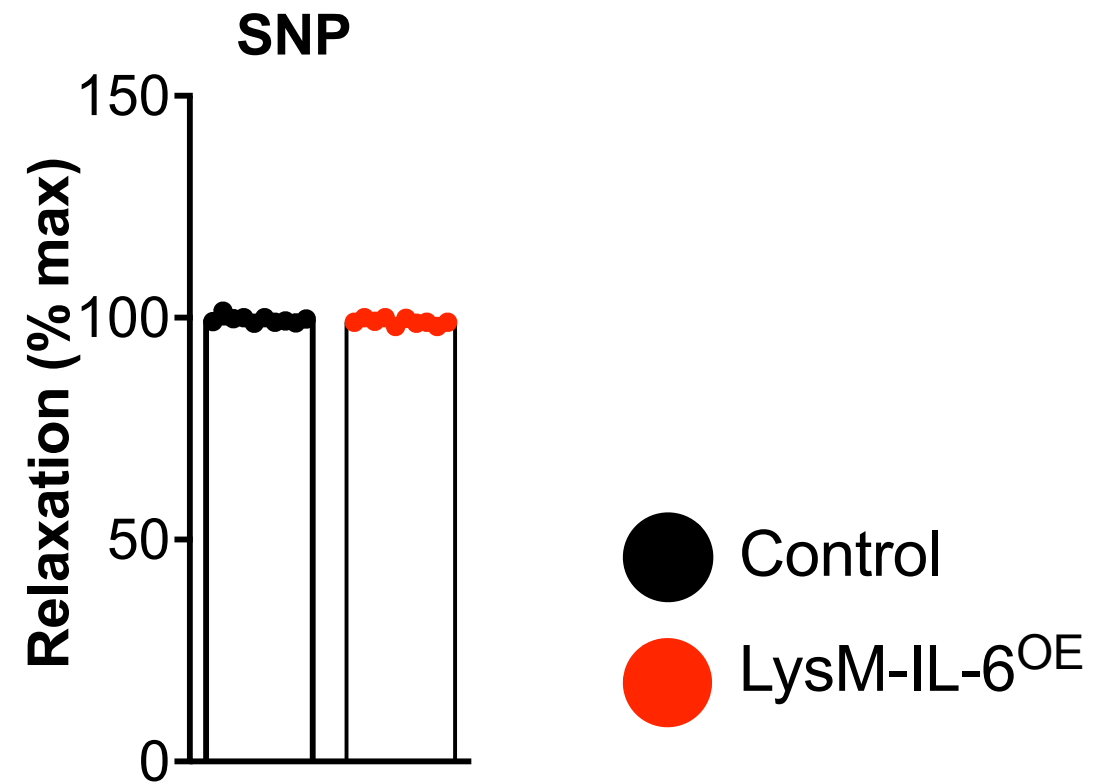

● Control  
● LysM-IL-6<sup>OE</sup>

Supplement: oeae046_Supplementary_Data [file oeae046_supplementary_data.zip › 2024-04-28 Suppl.Fig.3.pdf]

Supplementary Figure 4

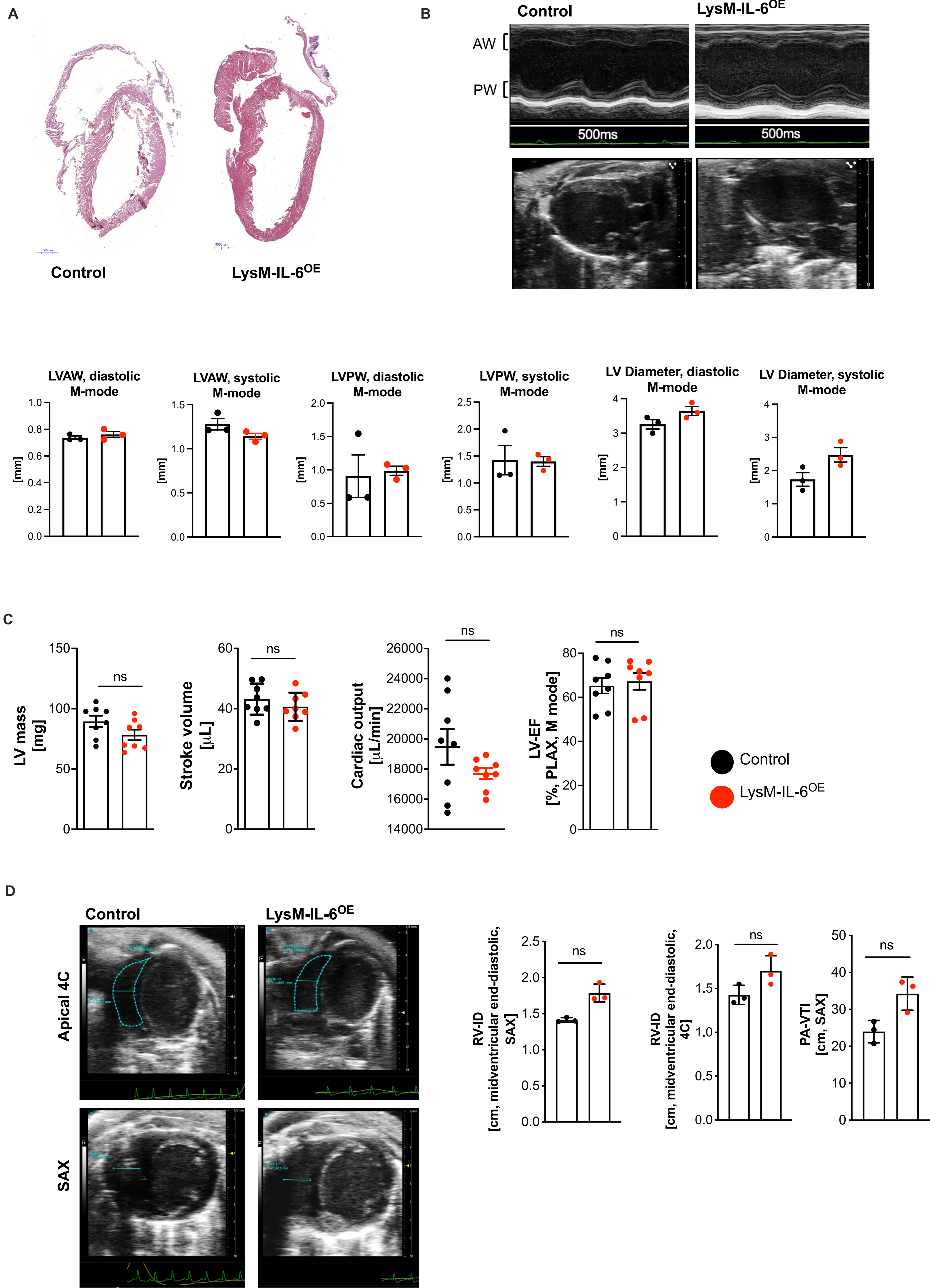

Supplement: oeae046_Supplementary_Data [file oeae046_supplementary_data.zip › 2024-04-28 Suppl.Fig.4.pdf]

Supplementary Figure 5

A

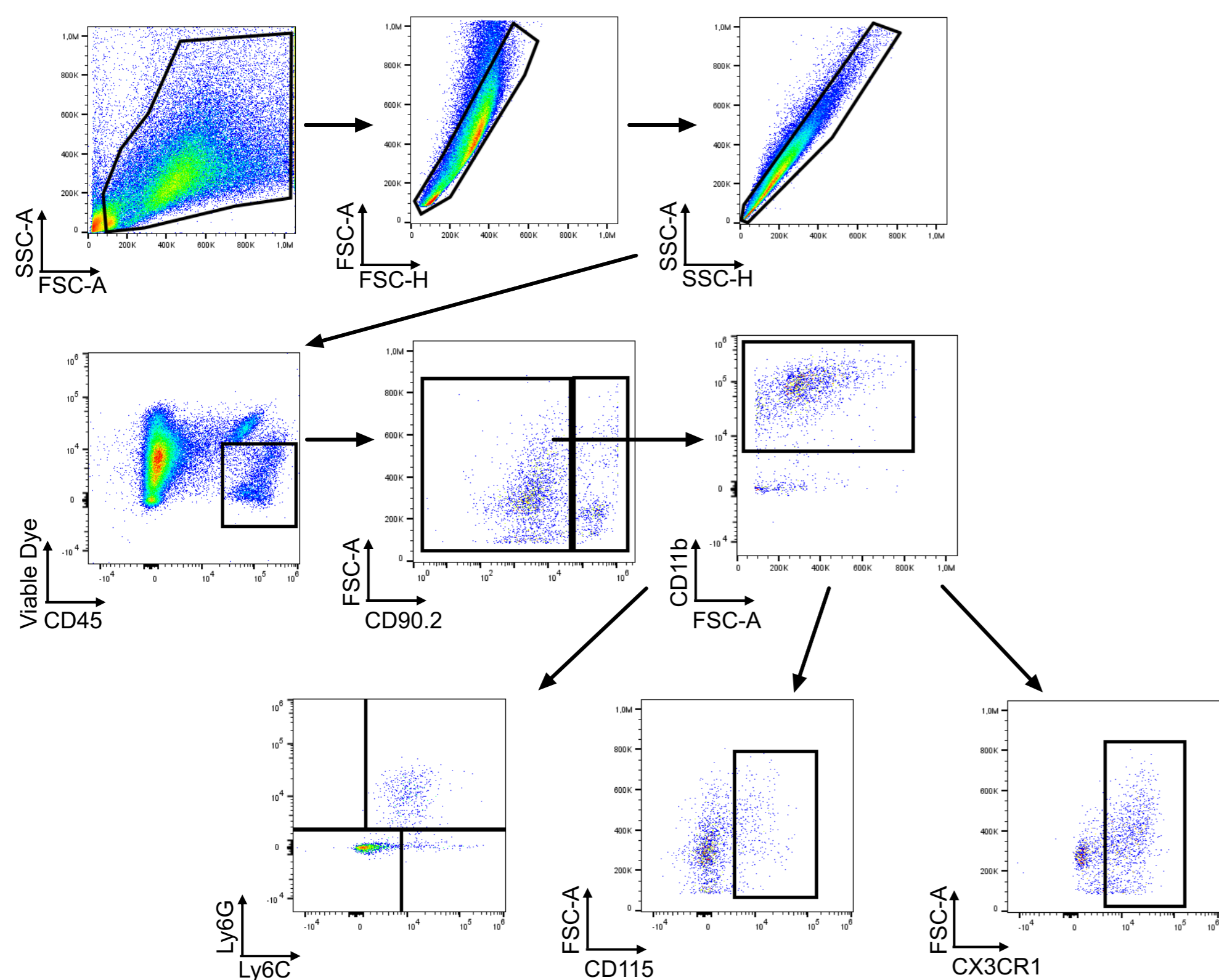

B

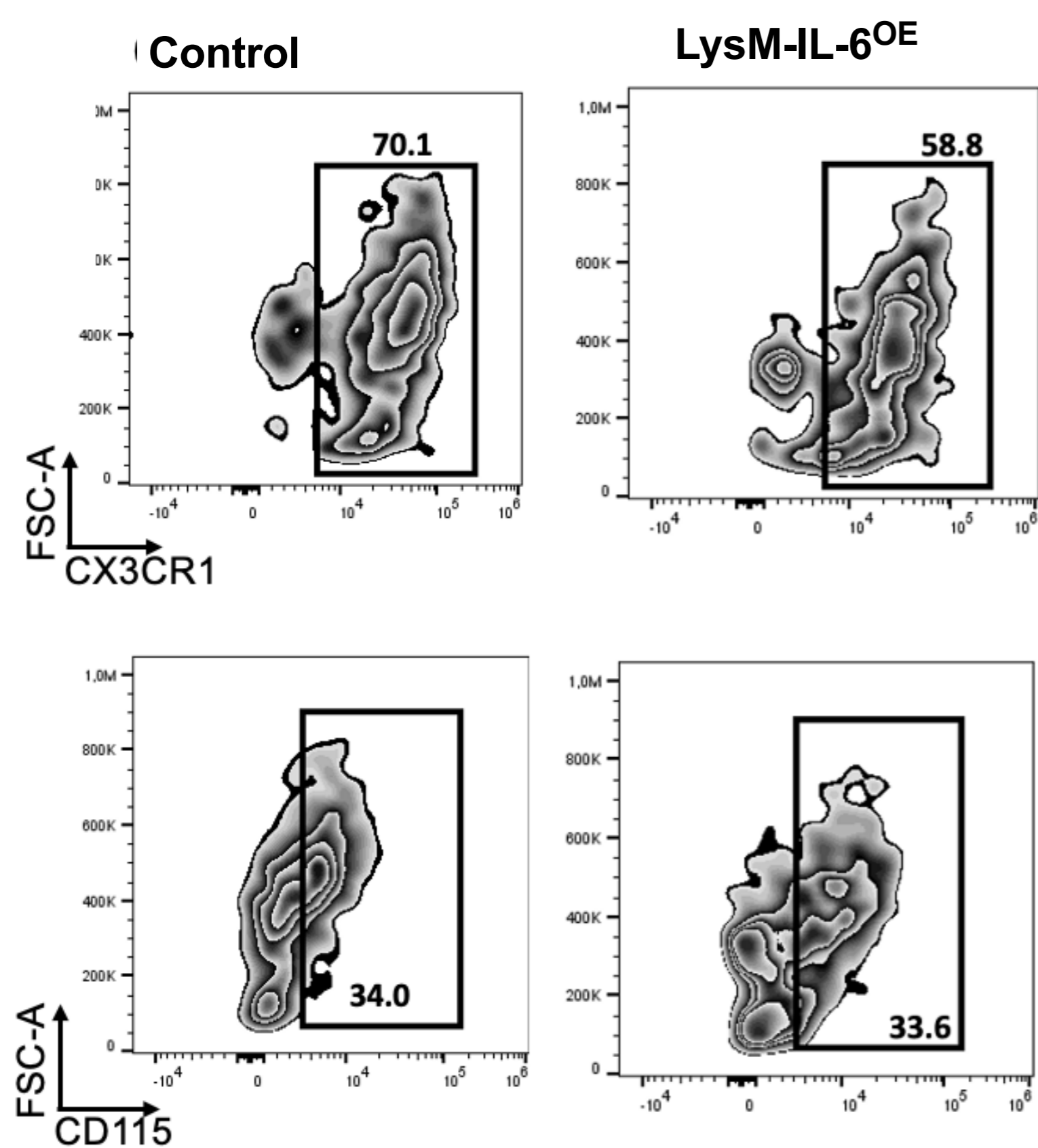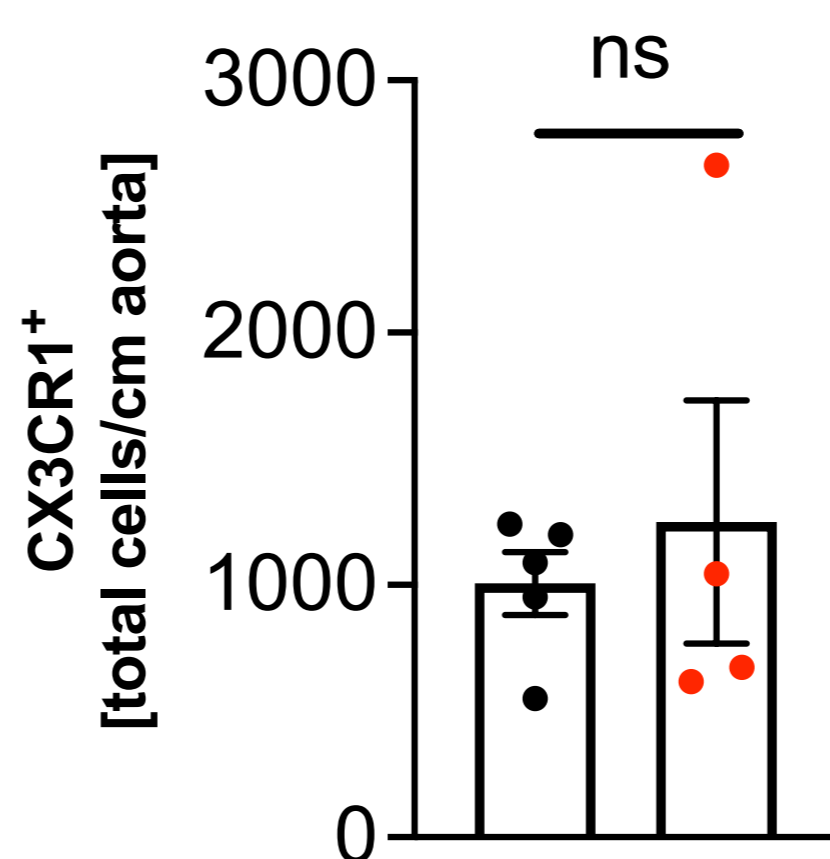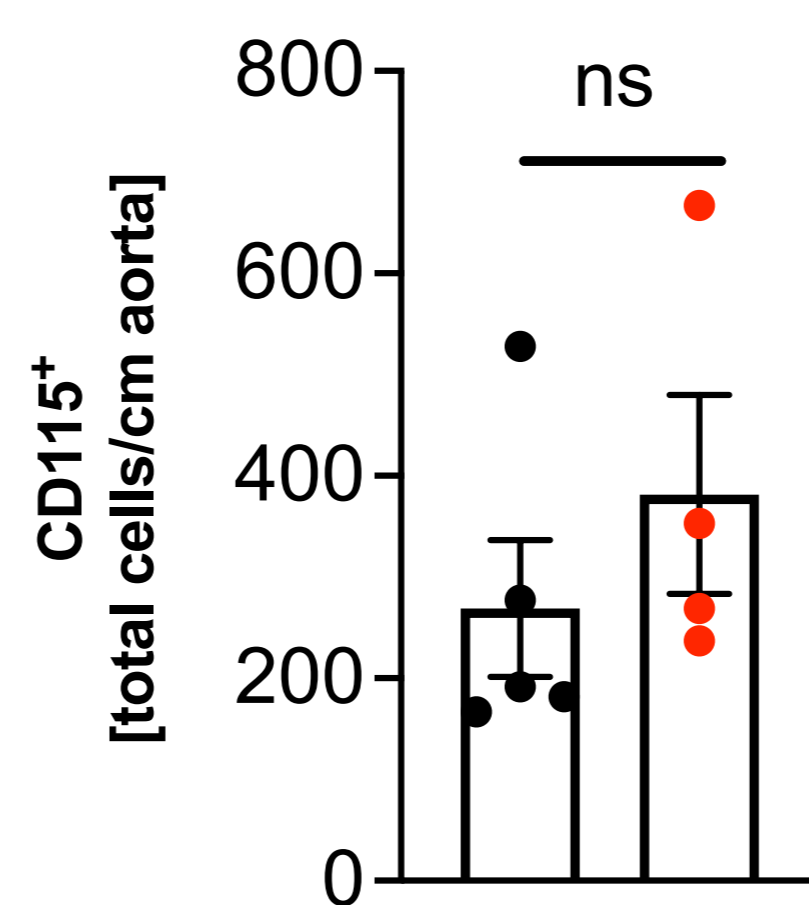

C

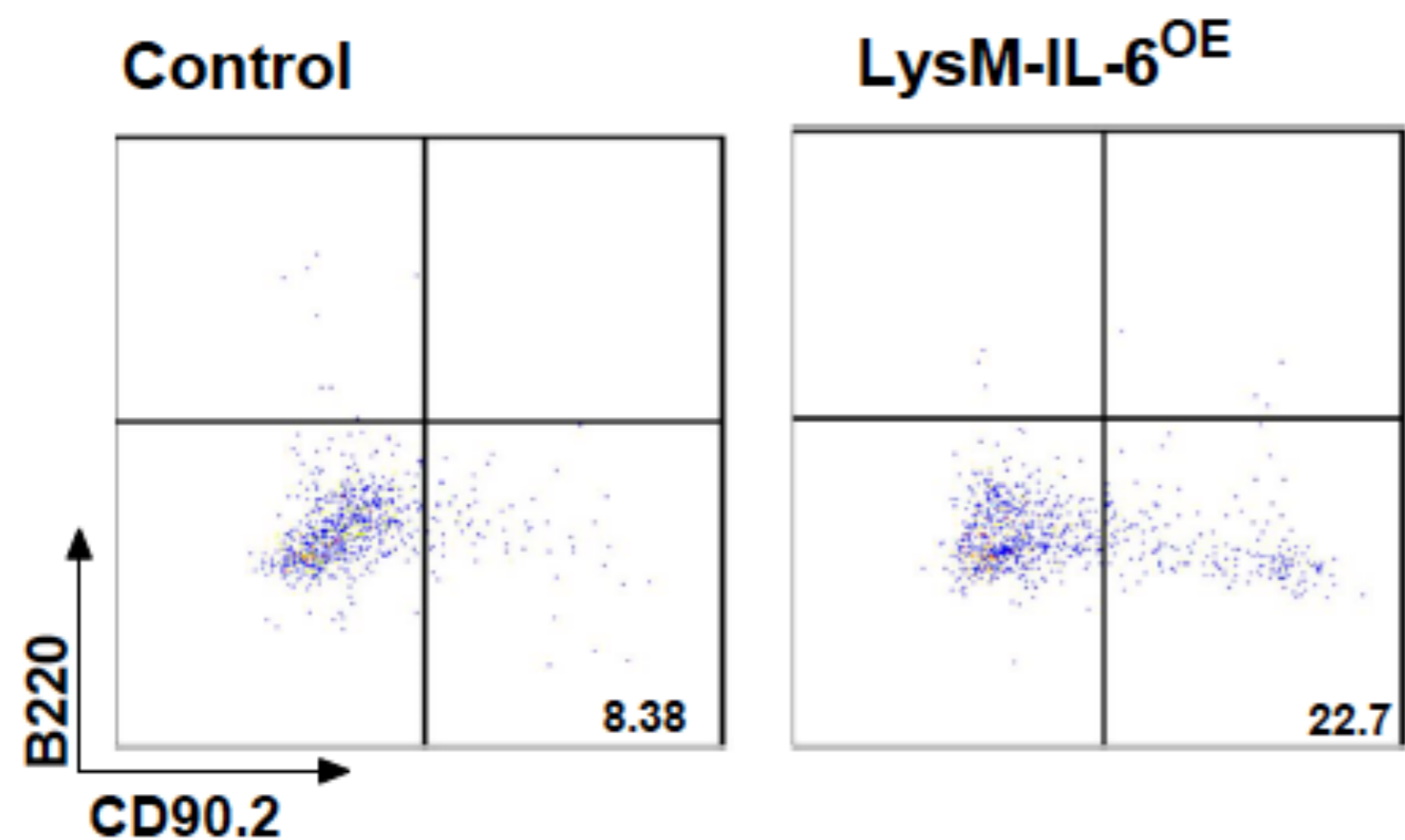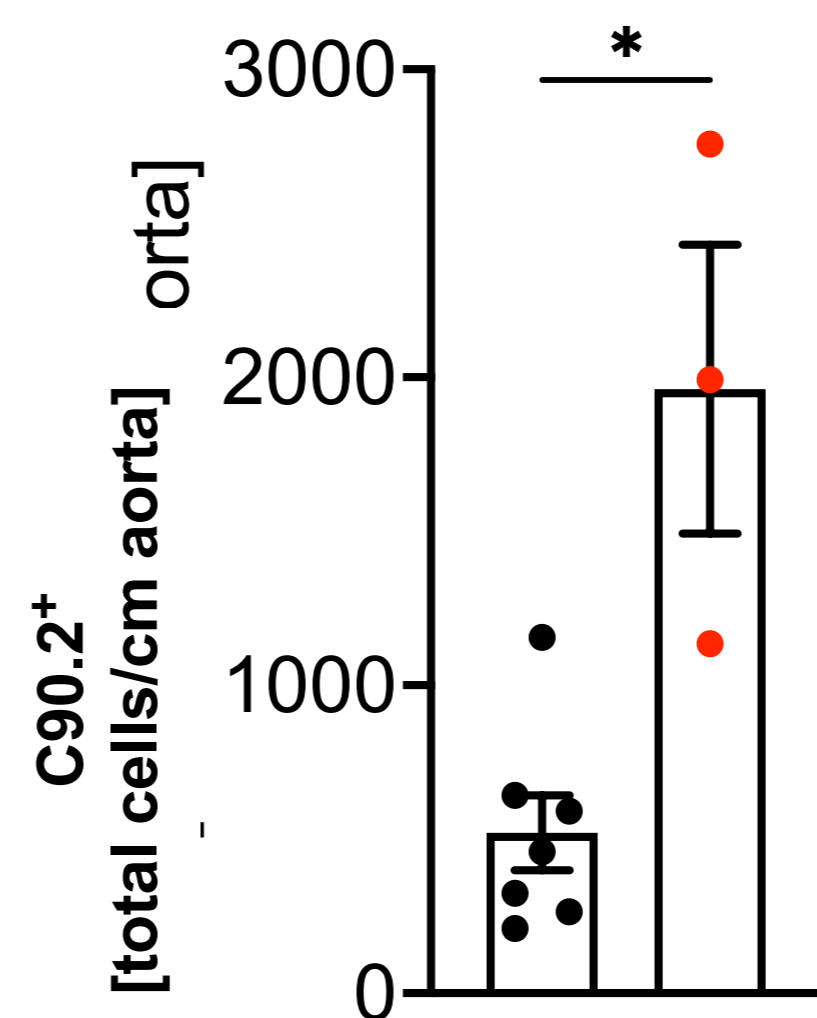

D

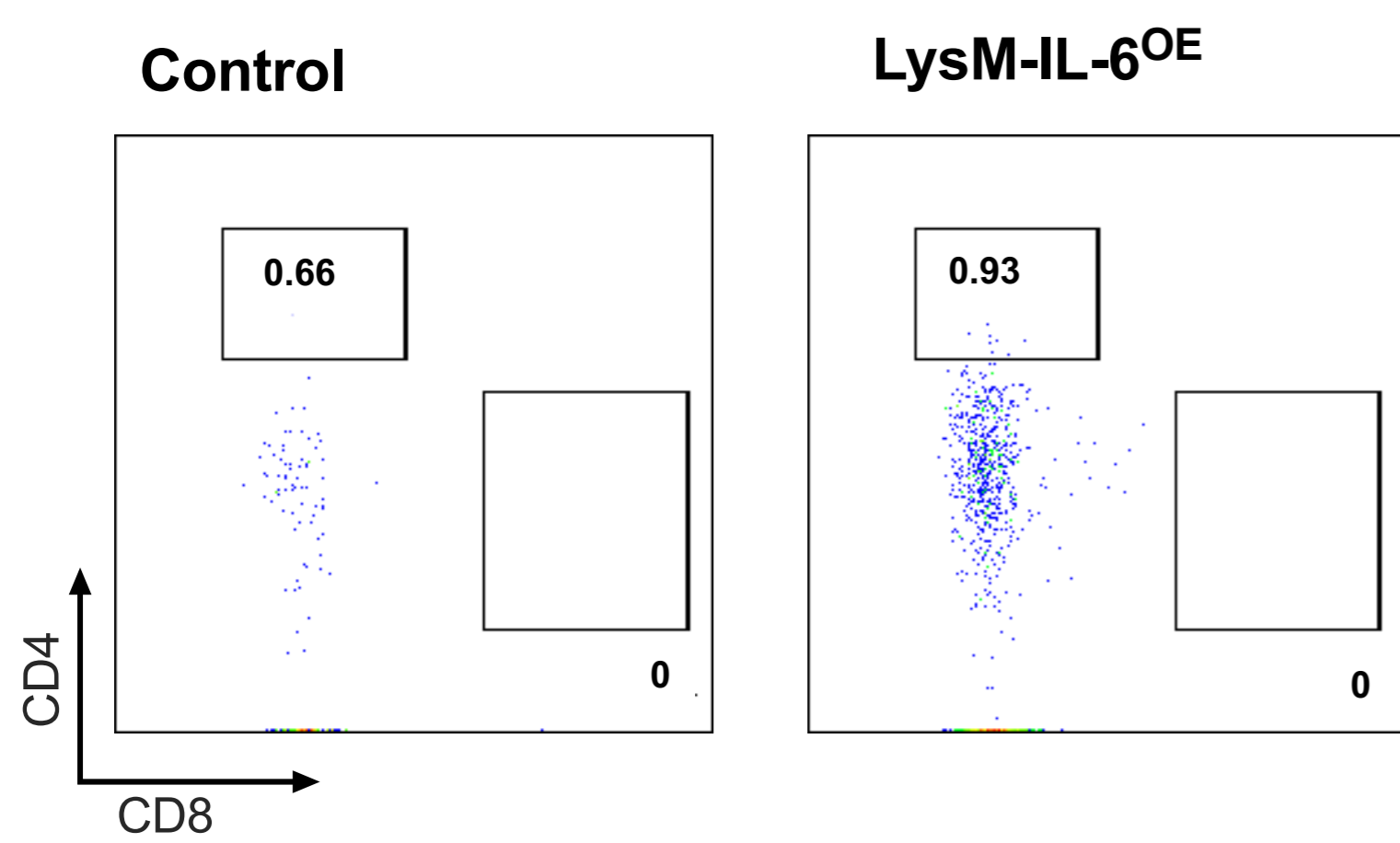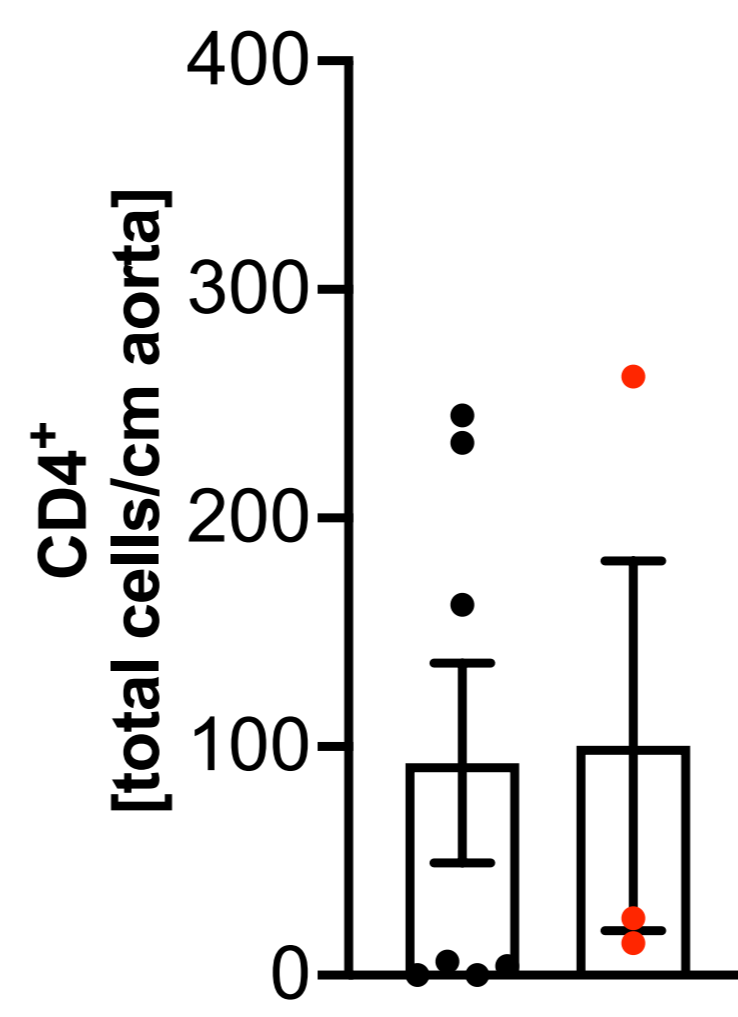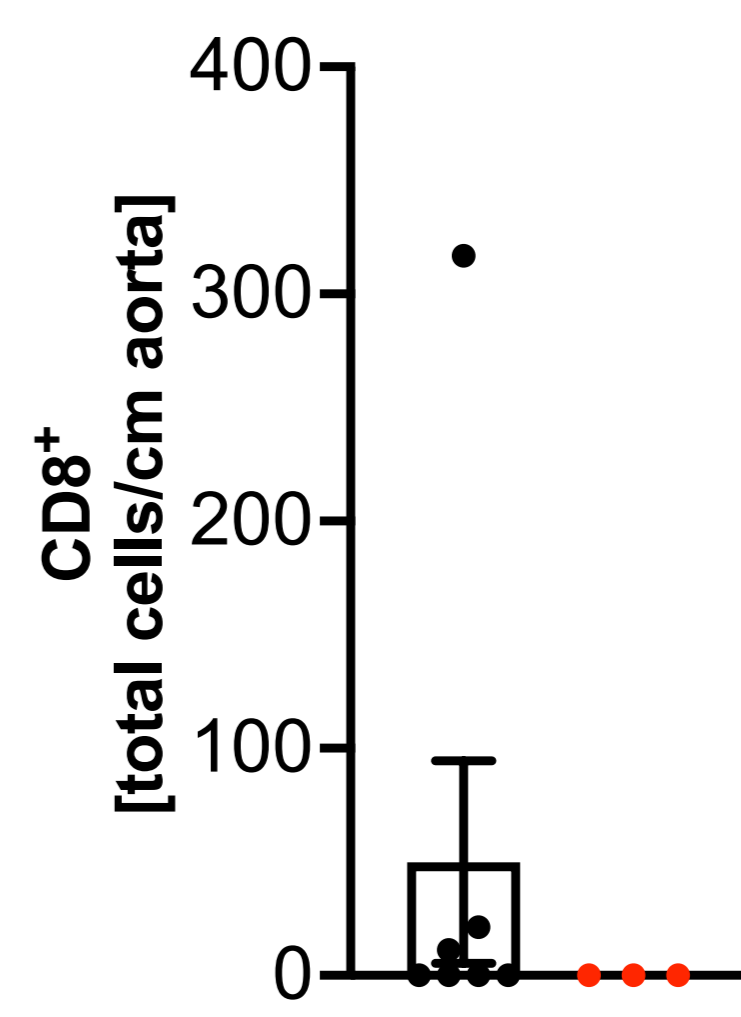

Supplement: oeae046_Supplementary_Data [file oeae046_supplementary_data.zip › 2024-04-28 Suppl.Fig.5.pdf]

## Supplementary Figure 6

# A

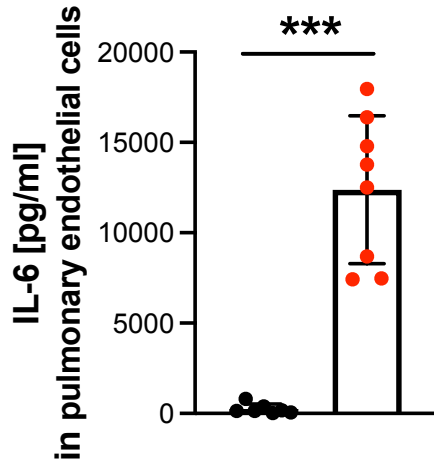

# B

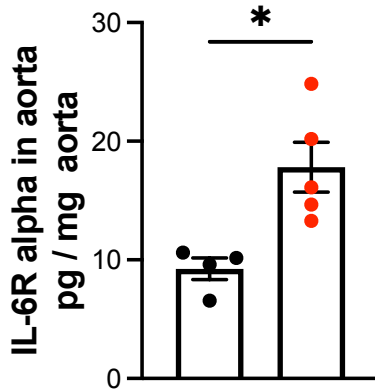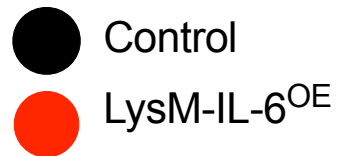

Supplement: oeae046_Supplementary_Data [file oeae046_supplementary_data.zip › 2024-04-28 Suppl.Fig.6.pdf]

# Supplementary Figure 7

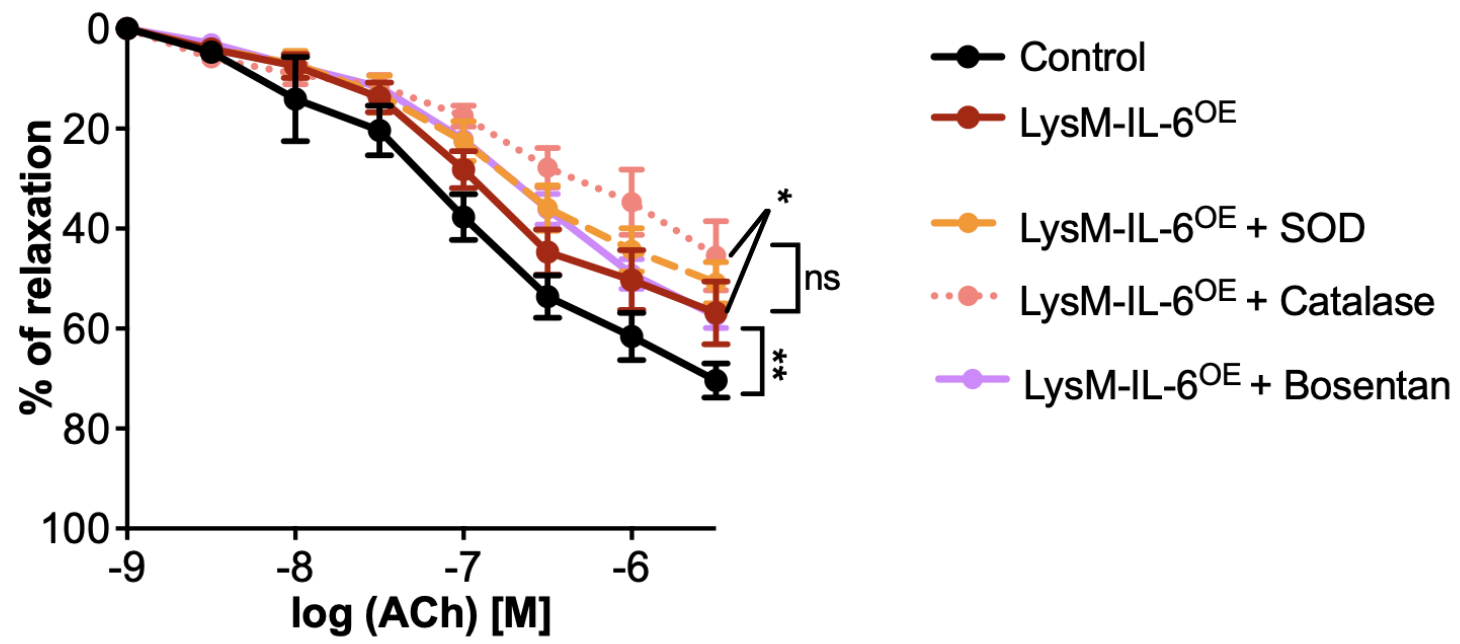

Supplement: oeae046_Supplementary_Data [file oeae046_supplementary_data.zip › 2024-04-28 Suppl.Fig.7.pdf]

# Supplementary Figure 8

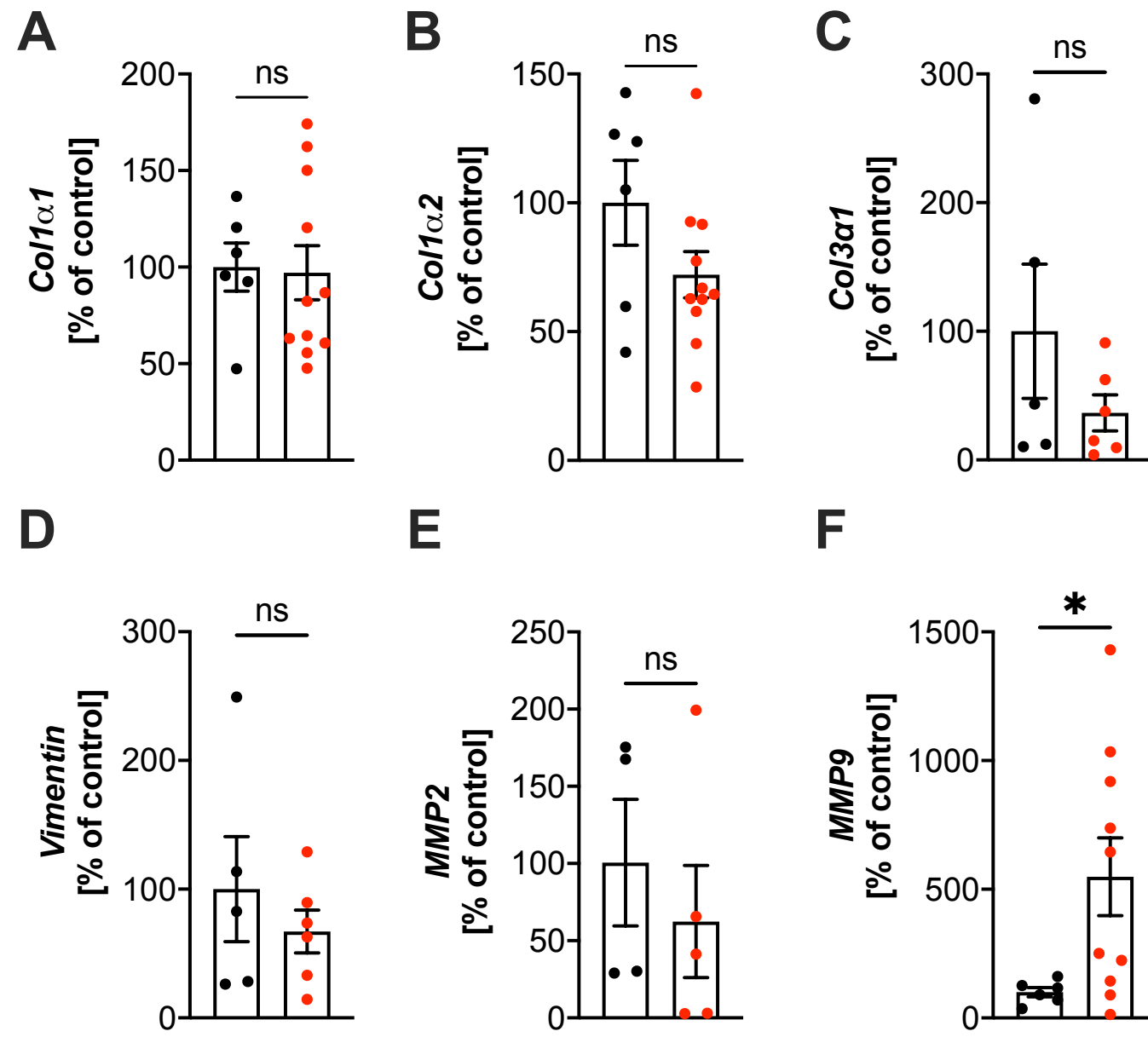

Supplement: oeae046_Supplementary_Data [file oeae046_supplementary_data.zip › 2024-04-28 Suppl.Fig.8.pdf]
